# Antineoplastic indole-containing compounds with potential VEGFR inhibitory properties†

**DOI:** 10.1039/d3ra08962b

**Published:** 2024-02-14

**Authors:** Dalia R. Aboshouk, M. Adel Youssef, Mohamed S. Bekheit, Ahmed R. Hamed, Adel S. Girgis

**Affiliations:** a Department of Pesticide Chemistry, National Research Centre Dokki Giza 12622 Egypt girgisas10@yahoo.com as.girgis@nrc.sci.eg; b Department of Chemistry, Faculty of Science, Helwan University Helwan Egypt; c Chemistry of Medicinal Plants Department, National Research Centre Dokki Giza 12622 Egypt

## Abstract

Cancer is one of the most significant health challenges worldwide. Various techniques, tools and therapeutics/materials have been developed in the last few decades for the treatment of cancer, together with great interest, funding and efforts from the scientific society. However, all the reported studies and efforts seem insufficient to combat the various types of cancer, especially the advanced ones. The overexpression of tyrosine kinases is associated with cancer proliferation and/or metastasis. VEGF, an important category of tyrosine kinases, and its receptors (VEGFR) are hyper-activated in different cancers. Accordingly, they are known as important factors in the angiogenesis of different tumors and are considered in the development of effective therapeutic approaches for controlling many types of cancer. In this case, targeted therapeutic approaches are preferable to the traditional non-selective approaches to minimize the side effects and drawbacks associated with treatment. Several indole-containing compounds have been identified as effective agents against VEGFR. Herein, we present a summary of the recent indolyl analogs reported within the last decade (2012–2023) with potential antineoplastic and VEGFR inhibitory properties. The most important drugs, natural products, synthesized potent compounds and promising hits/leads are highlighted. Indoles functionalized and conjugated with various heterocycles beside spiroindoles are also considered.

## Abstract 2

VEGF, an important category of tyrosine kinases, and its receptors (VEGFR) are hyper-activated in different cancers. The recently reported indolyl analogs with potential antineoplastic and VEGFR inhibitory properties are highlighted.

## Graphical abstract

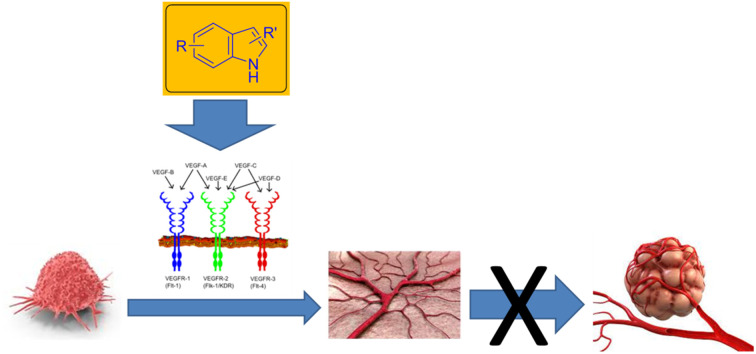

## 1. Introduction

Cancer is one of the most significant health challenges worldwide. It is the second most fatal disease globally after cardiovascular disorders.^1^ About 19–20 million people are diagnosed with different cancer types every year and many of them lose their life.^2^ Although many techniques, tools and therapeutics/materials have been developed in the last few decades, none of them are effective in controlling the various types of cancer at different stages, especially the advanced ones.^3^ To date, this challenge is still a significant task despite the great interest, funding and efforts from the entire scientific society (including research institutes and pharmaceutical companies).^4^ Recently, progress has been achieved in cancer chemotherapy due to the efforts devoted to developing selective molecular therapeutics. Although traditional non-selective therapeutics are still employed clinically, their drawbacks/side effects and poor survival rates are major problems, limiting their applications. Additionally, the early detection of this disease is challenging, although it is the most appropriate opportunity for curing it.^5,6^

To date, 90 protein tyrosine kinases have been identified among the known 518 kinases.^7^ Tyrosine kinases are capable of many diverse cellular functions including growth, proliferation, differentiation and death.^8^ Tyrosine kinases can catalyze the phosphorylation of tyrosine utilizing the adenosine triphosphate (ATP) molecule, which can be classified into receptor and non-receptor types. The receptor category include trans-membrane, extracellular and intracellular, whereas the non-receptors are intracellular.^9^ The overexpression of tyrosine kinases is associated with cancer proliferation and/or metastasis, indicating their importance as cancer chemotheraputics.^10,11^

Angiogenesis is an essential biological process for the formation/extension of new blood capillaries from the vessels present in the vascular system. Thus, it is an essential process for growth, menstruation, embryonic development, wound healing, functional repair and many pathological disorders including cancer. Furthermore, it is one of the main functions for delivering vital supplies including nutrients and oxygen to cells and removal of waste. Angiogenesis is also a critical process for tumor progression and metastasis. Many growth factors (VEGF: vascular endothelial growth factor, EGF: epidermal growth factor, and FGF: fibroblast growth factor) can stimulate angiogenesis.^12–15^ VEGF can be categorized into different classes (VEGF-A, B, C, and D), which can bind to diverse tyrosine kinase receptors (VEGFR-1, 2, and 3). Notably, VEGF and its receptors are hyper-activated in different cancers, and thus considered an important target for combating this disease. VEGFR-2 is the most well-known factor for angiogenesis of various solid tumors (colon, breast, ovary, lung, skin, renal, head, neck, lymphoma, *etc.*). Several drugs with inhibitory VEGFR-2 activity have been recognized and clinically approved as anticancer drugs.^12,13,16^ Anti-angiogenic active agents reduce the vascular permeability and enhance the extravasation of the therapeutic small molecules.^17^ However, some adverse effects have been reported to be associated with anti-VEGFR therapeutics including dermatologic disorders (skin rash, depigmentation and mucositis), painful hand-foot skin reactions and pruritus.^18^

Accordingly, multi-targeted inhibitory drugs/candidates have become a recent trend in cancer chemotherapy, attracting significant attention and research interest. Interest in this strategy is attributed to the multifactorial nature of many cancer types.^7^ Additionally, carcinoma cell initiation and proliferation involve various receptors and signaling pathways. Moreover, multi-target inhibitors can overcome cancer cell resistance, which is an advantage for multi-targeted treatment compared to single-targeted treatment or cocktail of multi-component drugs.^7,19^

**Fig. 1:**
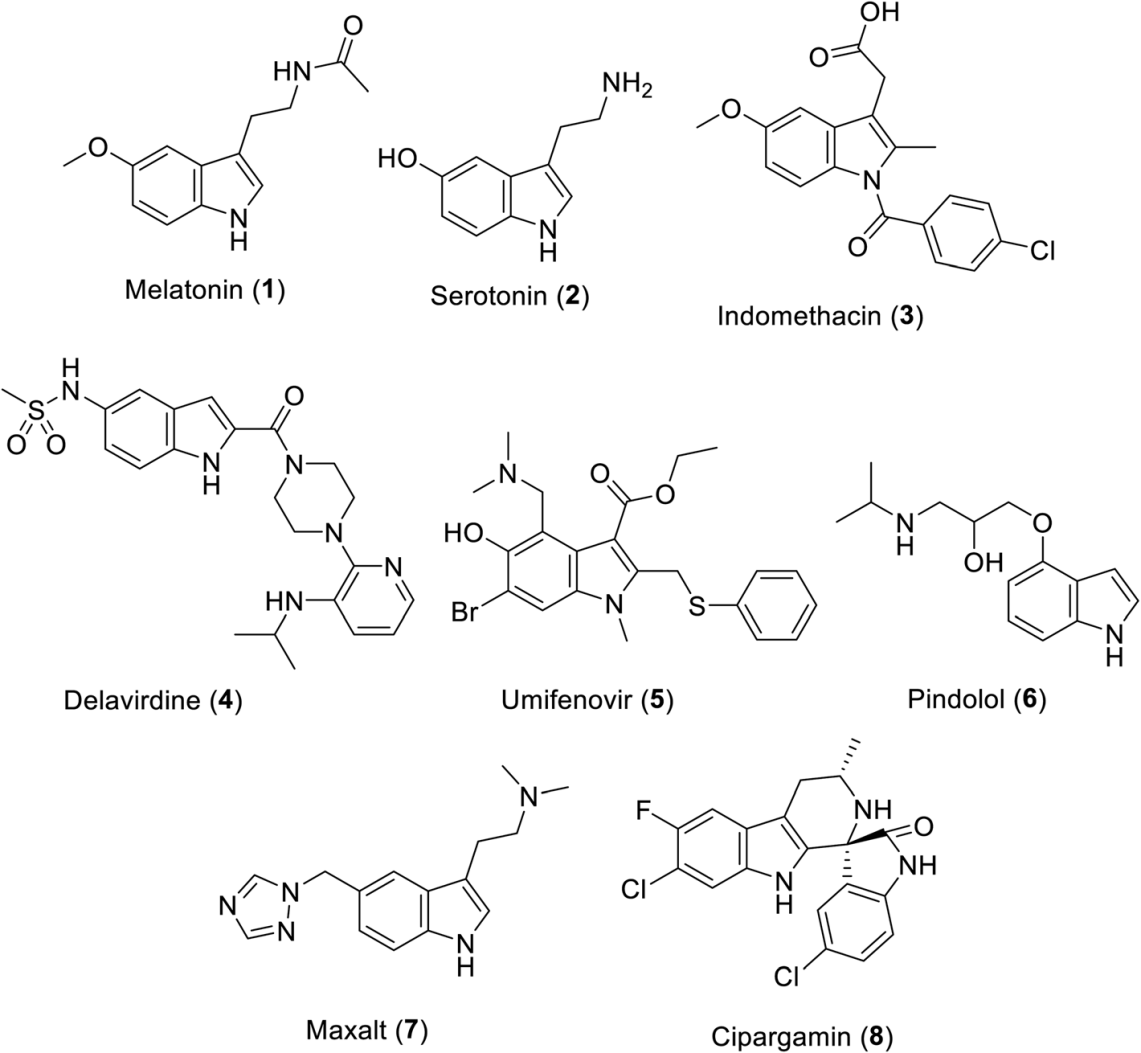
Clinically approved indole-containing drugs (1–7) and cipargamin (8).

The indolyl scaffold occupies a unique position among the diverse alkaloids due to the wide range of bio-properties of its natural and synthesized analogs. Many natural alkaloids are well known, among which melatonin 1 is a natural hormone biosynthesized in the dark by the pineal gland^20^ (Fig. 1). Recently, clinical trials supported its positive impact on the prevention or treatment COVID-19 infection upon being administered alone or in combination with other therapeutics.^21–24^ Serotonin 2 is a neurotransmitter that controls many human functions such as mood, appetite, sleep and social behavior.^25^

Many indole-containing drugs have been approved and in clinical use for a long time, among which indomethacin 3 is a famous non-steroidal anti-inflammatory drug (approved by the FDA “Food and Drug Administration” since 1965)^26^ with inhibitory properties against cyclooxygenase (COX), an enzyme controlling the formation of prostaglandin from arachidonic acid.^27^ Delavirdine 4 (FDA approval in 1997) is an anti-HIV (human immunodeficiency virus) drug.^28,29^ Umifenovir 5 (approved in Russia and China) is an anti-influenza drug repurposed for the treatment of SARS-CoV-2.^30–33^ Pindolol 6 (antihypertensive drug, non-cardioselective β-blocker, FDA approval in 1982)^34^ and Maxalt 7 (antimigraine agent, FDA approval in 1998)^35^ are also indole analogs. Cipargamin 8 exhibits high efficacy against protein synthesis in *Plasmodium falciparum*. In addition, it has entered pre-clinical trial investigations as a potential antimalarial drug.^36^

The current study aims to describe and highlight the indole-containing compounds with potential anti-VEGFR properties. Specifically, the relevant keywords were input in different search engines such as Scopus, ScienceDirect and PubMed. The recent advances in this subject (last decade, 2023–2012) with the greatest diversification revealing promising bio-properties will be discussed.

## 2. Indole-containing drugs and potent agents

### 2.1. Sunitinib

**Fig. 2:**
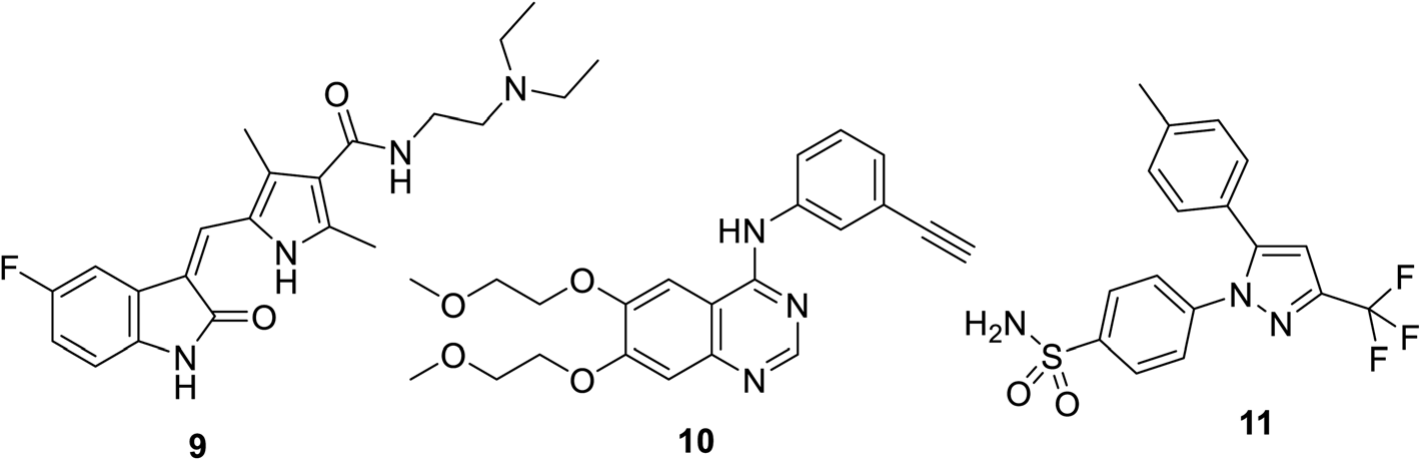
Sunitinib (sutent) 9 multi-targeted tyrosine kinase inhibitor, erlotinib 10 (EGFR inhibitor) and celecoxib 11 (COX-inhibitor).

Sunitinib (Sutent) 9 (Fig. 2) is an oral antitumor multi-targeted tyrosine kinase inhibitor (VEGFR-1,-2, -3; PDGFR-α, -β: platelet-derived growth-factor receptor; and c-kit: stem cell factor receptor) against FLT3, which is an FMS-like tyrosine kinase inhibitor with potent anti-angiogenesis properties.^16,37–39^ It is clinically approved against advanced renal and imatinib-resistant gastrointestinal (FDA approval in 2006) and pancreatic cancers (FDA approval in 2011). It has also been given FDA approval (2017) for adult adjuvant treatment at high risk of renal cancer.^40,41^ However, due to the side effects/drawbacks (diarrhea, fatigue, hypertension, hematologic toxicities, cardiotoxic effects and hand-foot syndrome)^39,42–44^ observed during clinical administration, many studies proposed the combination of sunitinib with another chemotherapeutic agent or radiation. This approach can reduce the unintended side effects and enhance the efficacy of the drug.^42^

Also, sunitinib can be useful in the treatment of melanoma, which was supported by pre-clinical studies revealing the initiation of tumor hypoxia in melanoma xenografts.^16^ An obvious objective response by solid tumors to sunitinib including metastatic breast, colon, neuroendocrine and non-small cell lung cancers (NSCLC) was reported.^39,42,45^

A synergistic effect was observed upon the combination of sunitinib with erlotinib 10 (EGFR: epidermal growth factor receptor, inhibitor) for the treatment of NSCLC A549 xenograft mice^46^ (Fig. 2). Pre-clinical model studies supported that the combination of PRX177561 (CXC4, chemokine receptor type 4 antagonist) with sunitinib enhanced the therapeutic efficacy (reducing the tumor proliferation and extending the disease-free survival) against glioblastoma (brain cancer).^47,48^*In vivo* studies supported that a COX-2 (cyclooxygenase-2) inhibitor (celecoxib 11) can enhance the activity of sunitinib in mice bearing human renal cancer xenografts *via* the observation of delay in tumor progression.^49^

### 2.2. Nintedanib

**Fig. 3:**
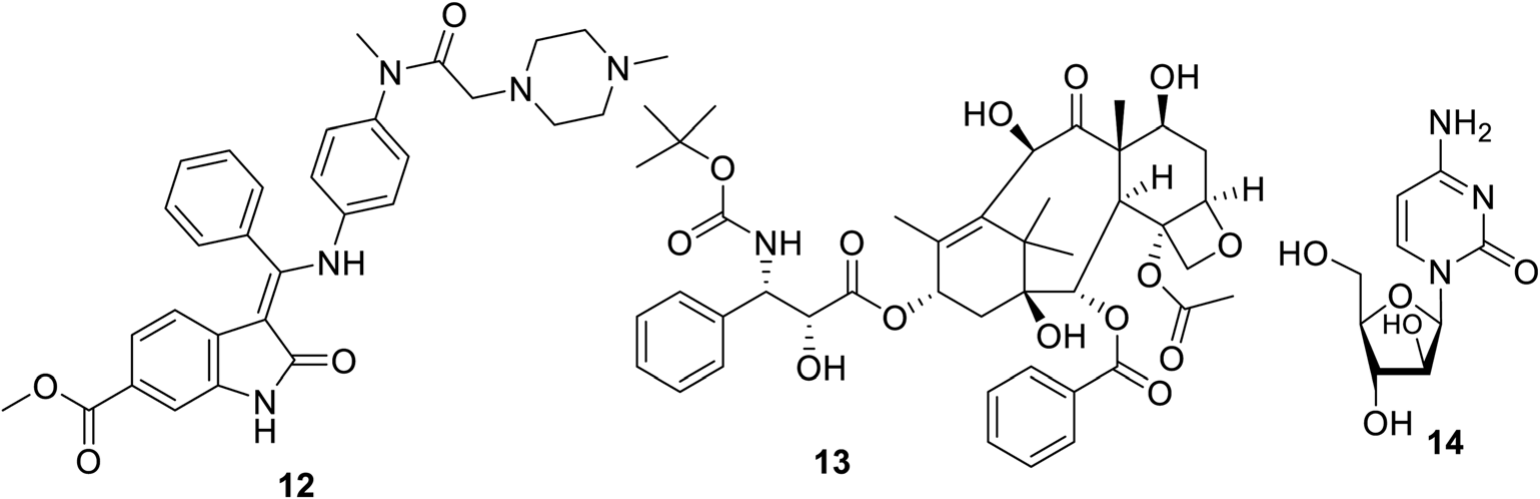
Nintedanib 12 (multi-targeted) tyrosine kinase inhibitor, docetaxel 13 and cytarabine 14.

**Fig. 4:**
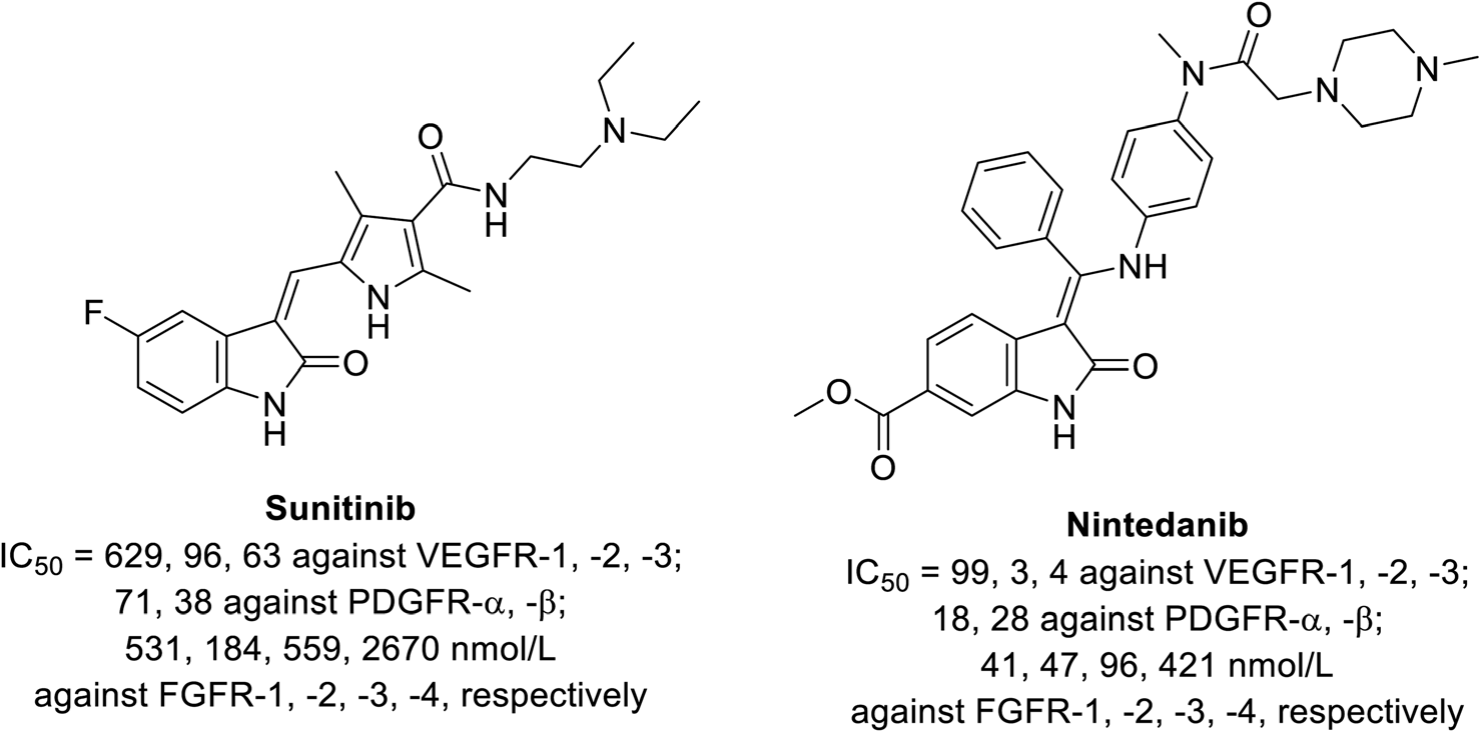
Inhibitory properties of sunitinib and nintedanib against tyrosine kinases (VEGFR-1, -2, -3; PDGFR-α, -β and FGFR-1, -2, -3, -4).^52^

Nintedanib 12 (Ofev and Vargatef) is also an oral multi-targeted tyrosine kinase inhibitor (VEGFR-1, -2, -3; PDGFR-α, -β and FGFR-1, -2, -3, -4: fibroblast growth-factor receptor)^50–54^ (Fig. 3 and 4). The FDA approved its use for the treatment of idiopathic lung fibrosis (2014), systemic sclerosis-associated interstitial lung disease (2019) and NSCLC (in combination with docetaxel 13).^50,52–56^ However, the most notable side effects are diarrhea and increase in alanine and aspartate aminotransferase associated with the clinical administration of nintedanib.^54^

Many studies explained the promising efficacy of nintedanib against different types of cancer. The delay of the proliferation and growth arrest of a mouse prostate (TRAMP: transgenic adenocarcinoma of the mouse prostate) model by nintedanib indicates its promising properties against prostate cancer.^57,58^ Its clinical trial (phase III) also support its promising properties against ovarian cancer.^59,60^ A compensatory role was reported for nintedanib towards metastatic colon cancer.^61^ Meanwhile, clinical trial observation (20 patients, 200 mg twice per day) revealed that there was no considerable effect on salivary gland cancer except in controlling the rate of the disease.^62^ A phase I clinical study (13 elderly patients) also showed its efficacy towards myeloid leukemic cells, especially when used with cytarabine 14.^63^

### 2.3. Anlotinib

**Fig. 5:**
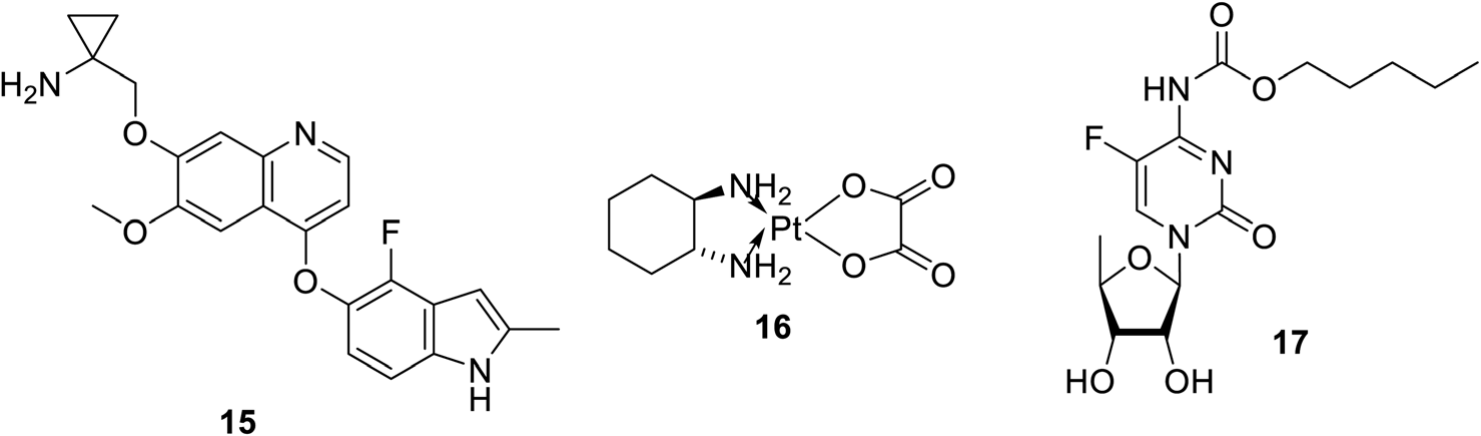
Anlotinib 15 (a multi-targeted tyrosine kinase inhibitor), oxaliplatin 16 capecitabine 17.

Anlotinib 15 (Fig. 5) is an oral multi-targeted tyrosine kinase inhibitor (VEGFR, PDGFR, FGFR and c-kit) approved in China for NSCLC.^64–69^ Hypertension and gastrointestinal problems are the most significant side effects associated with its administration.^65^ Finger print loss for about two months was observed during a case study on its treatment of lung cancer.^65^ It has also been reported that anlotinib is capable of inhibiting lymphangiogenesis and lymphatic metastasis, which is probably due to the suppression of VEGFR-3 phosphorylation.^70^ Antiproliferation properties were also observed against colon cancer cells (HCT-116 and LOVO).^71^ Moreover, anlotinib showed promising properties against thyroid and metastatic renal cell cancers.^68,72^ A phase II clinical study supported the possibility for the therapeutic utilization of anlotinib in combination with oxaliplatin 16 and capecitabine 17 to treat patients with metastatic colon cancer.^73^

### 2.4. Surufatinib (HMPL-012, sulfatinib)

**Fig. 6:**
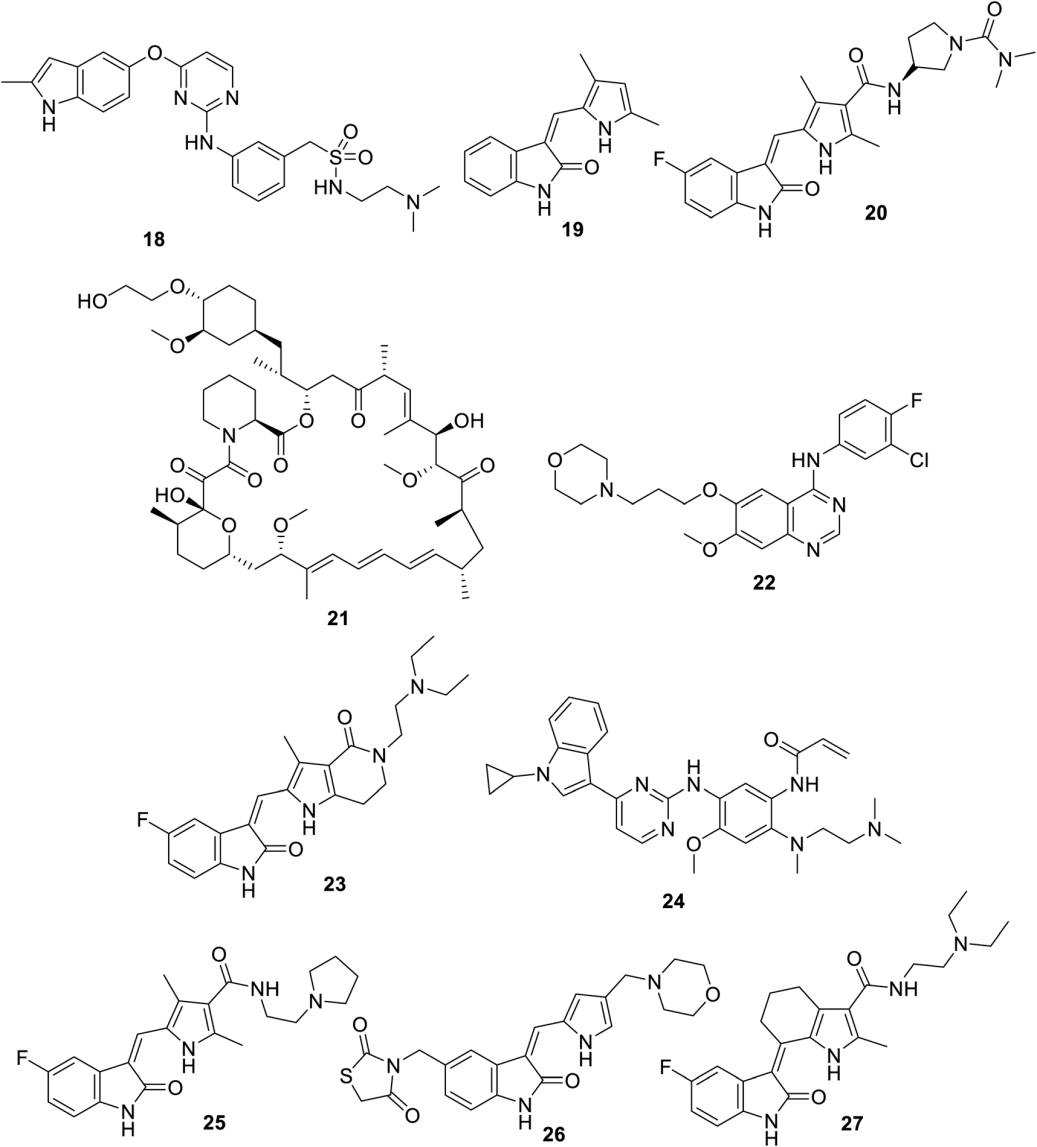
Chemical structures of surufatinib 18, SU5416 (semaxanib) 19, vorolanib (CM082) 20, everolimus 21, gefitinib (Iressa) 22, famitinib 23, almonertinib (HS-10296) 24, toceranib (Palladia, Zoetis) 25, S49076 26 and SIM010603 27.

Surufatinib (Sulanda) 18 (Fig. 6) is an oral multi-kinase inhibitor with dual function against angiogenesis (VEGFR-1, -2, -3 and FGFR) and tumor immune evasion (CSF-1R: colony stimulating factor-1 receptor). It is approved (in China, 2020) for the treatment of extrapancreatic neuroendocrine tumor (NET).^74^ However, it has been filed with the FDA for approval for the treatment of advanced NET (2021).^75^ Hypertension and proteinuria are the most severe adverse effects reported with the administration of Sulanda.^76^ A phase II clinical study adopting 39 patients (300 mg, 28 day cycles, once daily) supported its possibility for moderate biliary tract cancer.^77^

### 2.5. SU5416 (semaxanib)

SU5416 (semaxanib) 19 (Fig. 6) is a human solid tumor antiangiogenic VEGFR inhibitor under investigation.^78–80^ Its antiproliferation of murine cardiac endothelial cells was reported.^80^ Also, pathophysiological effects in respiratory disorders were mentioned, which was supported by the lipopolysaccharide-induced acute lung injury in mice *via* restrain/modulate vascular permeability.^79^

### 2.6. Vorolanib

Vorolanib (CM082) 20 (Fig. 6) is an oral VEGFR and PDGFR inhibitor.^81^ A phase I study (22 patients) exhibited promising results for the combination of vorolanib with everolimus 21 towards renal and neuroendocrine cancers.^82^ It has also mentioned that vorolanib enhances the antiproliferation and apoptosis properties of gefitinib (Iressa) 22 (EGFR: epidermal growth factor receptor, inhibitor) towards NSCLC cell lines (HCC827 and H3255). This was explained by the strong inhibitory properties of the combined therapeutics on STAT3 phosphorylation compared to that of the mono-therapeutic.^83^

### 2.7. Famitinib

Famitinib 23 (Fig. 6) is an oral VEGFR-2 and -3 inhibitor used in trial studies against colon and renal cancers.^84,85^ A promising synergistic effect was observed against NSCLC upon the combination of famitinib with almonertinib (HS-10296, EGFR inhibitor) 24.^85^

### 2.8. Toceranib

Toceranib (Palladia, Zoetis) 25 (Fig. 6) is a multi-targeted tyrosine kinase inhibitor (VEGFR-2, PDGFRs and c-Kit) used as a phosphate salt for the treatment of bone cancer (canine osteosarcoma) in veterinary medicine (dogs). Cell growth inhibition of toceranib phosphate on canine osteosarcoma (Penny and Wall) *in vitro* was reported.^86^

### 2.9. S49076

S49076 26 (Fig. 6) is a VEGF and HIF-1-α (hypoxia-inducible factor 1-alpha) inhibitor.^87^ The inhibition of VEGF and HIF-1 expression can explain the mode of action of S49076 in ovarian cancer cells.^87^ The administration of S49076 as a monotherapeutic resulted in the arrest of colon bevacizumab-resistant tumor growth. Moreover, the combination of S49076 and bevacizumab (Avastin, anti-vascular endothelial growth factor antibody) showed total growth inhibition of colon cancer xenograft models.^88^ A phase I study (103 patients) also revealed its effect on solid tumors (colon, lung, mesothelioma and uveal melanoma) upon oral administration.^89^

### 2.10. SIM010603

SIM010603 27 (Fig. 6) is an oral multi-targeted tyrosine kinase inhibitor (VEGFR-2, -3; PDGFR-β and stem cell factor receptor “c-kit”).^90^ Antiproliferation properties were reported against NCI-H460 (human lung), LLC-SW44 (Lewis lung) and MDA-MB-435 (breast) cancer cells in addition to the inhibition of xenograft tumor growth models and angiogenesis in mice.^90^ Adverse effects including gastrointestinal, pancreatic and skeletal toxicities, bronchopneumonia and cardiovascular dysfunction were mentioned in the toxicological studies of SIM010603 in rats and dogs (0–20 and 0–10 mg kg^−1^ per day oral administration, respectively, for 28 followed by 14 recovery days). However, no mortality rates were recorded for dogs receiving 10 mg kg^−1^.^91^

## 3. Natural indole-containing compounds

**Fig. 7:**
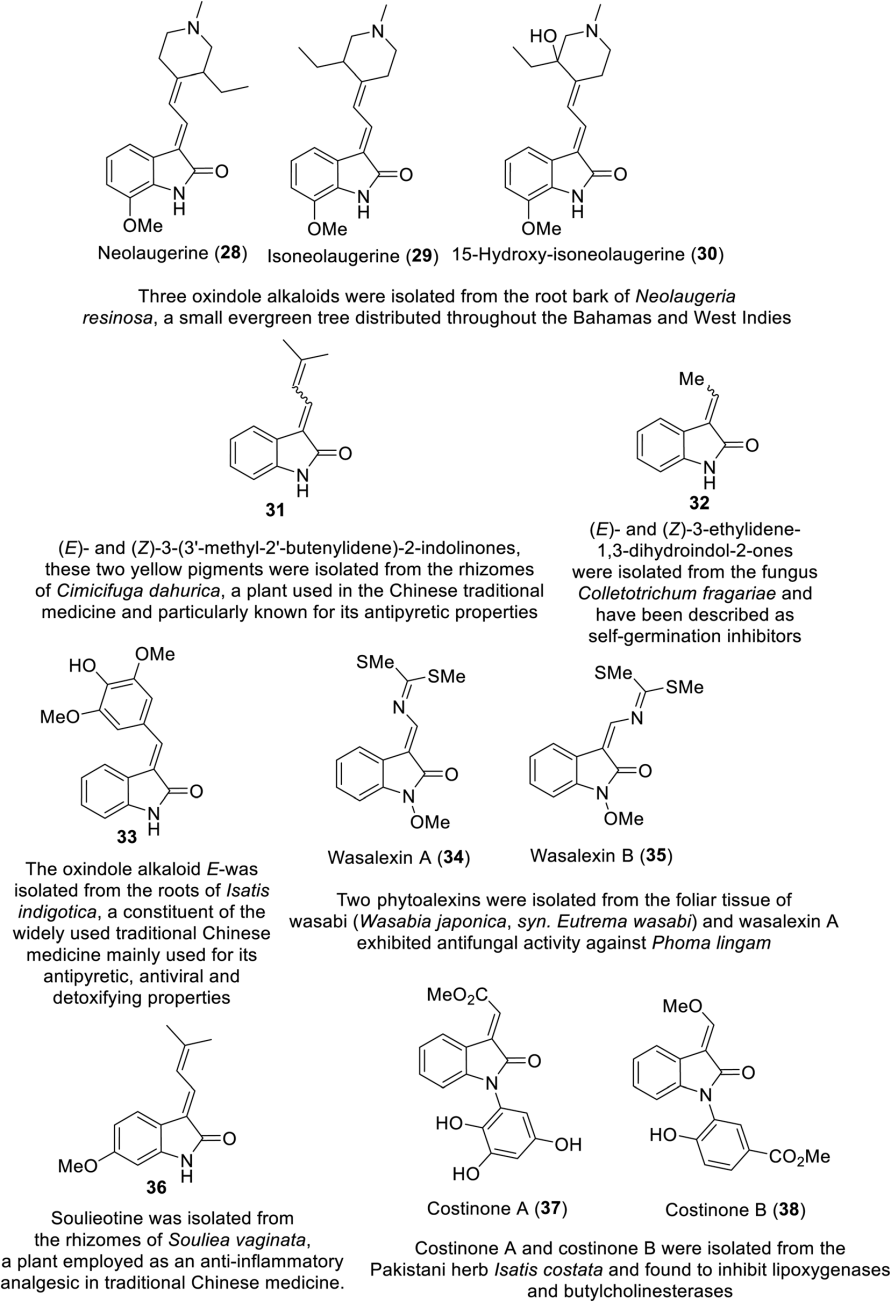
Natural indole-containing compounds 28–38 with potential bio-properties.

**Fig. 8:**
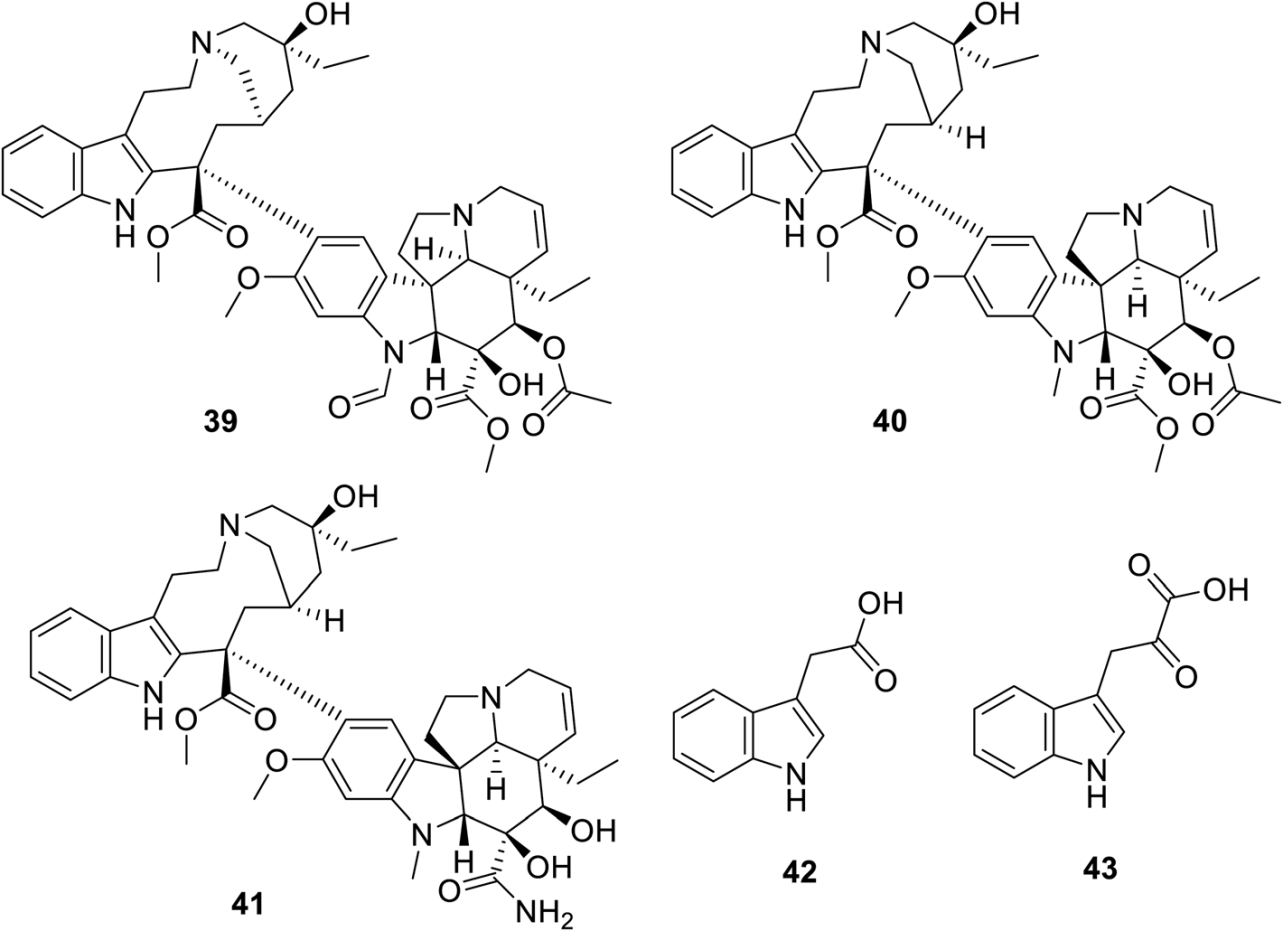
Chemical structures of vincristine 39, vinblastine 40, vindesine 41, 3-indole acetic acid 42 and 3-indole pyruvic acid 43.

Many natural indolyl derivatives have been identified to possess considerable bio-properties including 28–38, as summarized in Fig. 7.^92^ Vincristine 39 (Fig. 8) was isolated from *Vinca rosea* and approved by the FDA (since 1963) for the treatment of Hodgkin's disease, non-Hodgkin's lymphoma and neuroblastoma.^93–95^ Vinblastine (Velban) 40 is also a *Vinca* alkaloid approved by the FDA (since 1965) for the treatment of Hodgkin's disease, lymphoma, and testicular and breast cancers.^95–97^ Vindesine (Eldisine) 41 is a *Vinca* alkaloid with efficacy against acute lymphocytic leukemia.^95,98^ 3-Indole acetic acid 42, which is present in wine, and 3-indole pyruvic acid 43 (human metabolite) exhibited inhibitory properties against VEGFR-2 with IC_50_ = 0.9704 and 1.037 mM, respectively^99^ (Fig. 8).

## 4. Synthesized indole-containing compounds

### 4.1. Indolecarboxamide

**Scheme 1:**
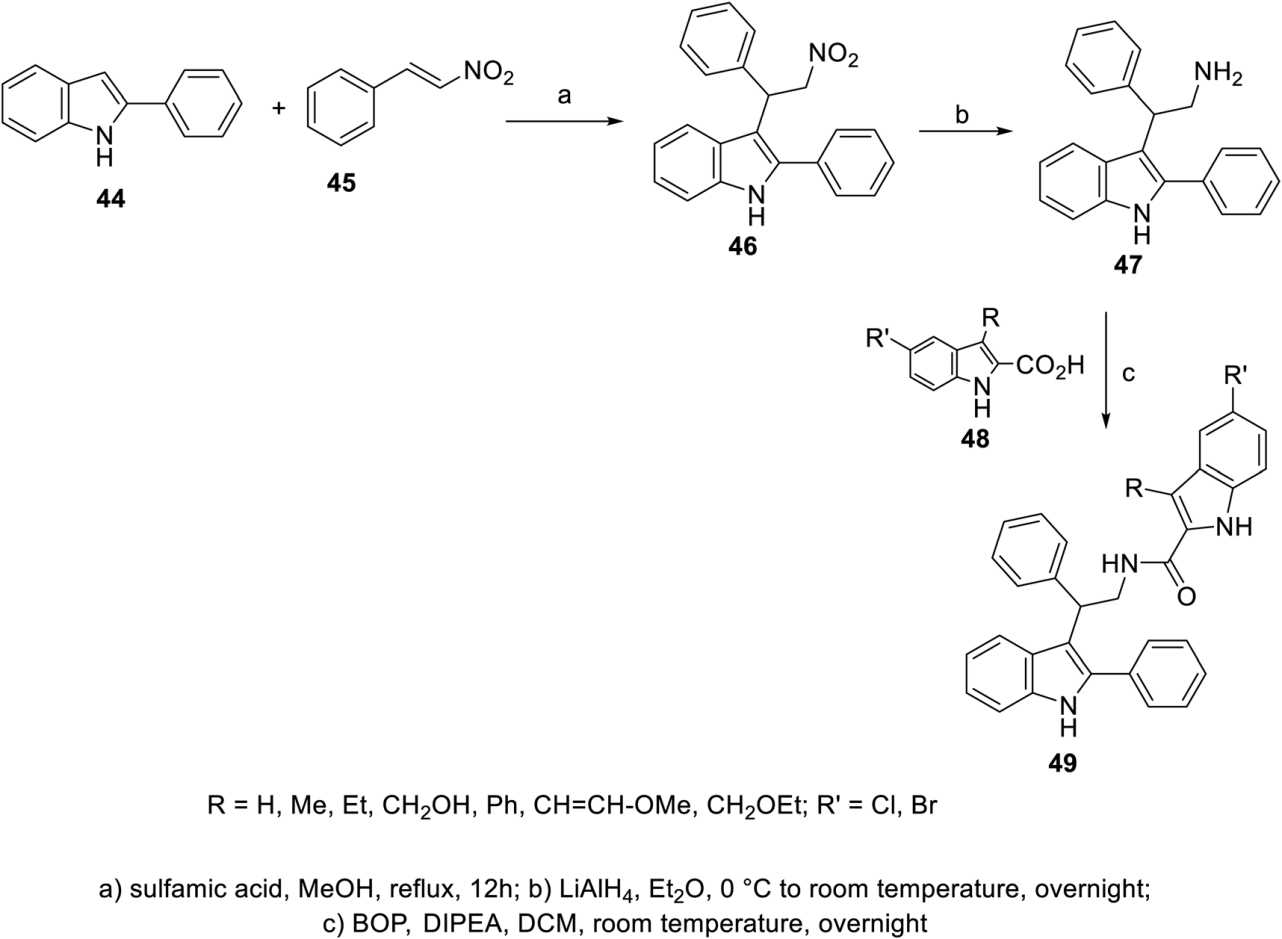
Synthesis of indole-2-carboxamides 49.

A variety of indole-2-carboxamides 49 was synthesized through the reaction of 2-(1*H*-indol-3-yl)ethan-1-amine 47 with 2-indolecarboxylic acids 48 in DCM (dichloromethane) containing BOP (benzotriazol-1-yloxytris(dimethylamino)phosphonium hexafluorophosphate) and DIPEA (diisopropyl ethylamine). Compound 47 was obtained through the reduction of nitro analog 46 by LiAlH_4_ in Et_2_O (at 0 °C to room temperature) under an inert (nitrogen) atmosphere. The latter 46 was synthesized by the addition reaction of 2-phenylindole 44 to β-nitrostyrene 45 in refluxing MeOH containing sulfamic acid^100^ (Scheme 1). Greater antiproliferation properties against a variety of human tumor cell lines [MTT: 3-(4,5-dimethylthiazol-2-yl)-2,5-diphenyltetrazolium bromide assay, Panc-1 (pancreatic), MCF7 (breast), HT-29 (colon) and A-549 (epithelial)] were revealed (IC_50_, nM ± SEM “standard error mean”) for some of the prepared 2-indolecarboxamides 49 compared to that of erlotinib. Also, mild VEGFR-2 inhibitory properties were observed by some of the derivatives of 49 relative to that of sorafenib. The most considerable agent was 49e (R = CH_2_OH, R′ = Cl; IC_50_ = 44 ± 4, 46 ± 4, 45 ± 4, and 42 ± 4 nM against Panc-1, MCF7, HT-29 and A-549, respectively; IC_50_ = 1.10 ± 0.08 nM against VEGFR-2) (ESI Fig. S1†). Based on the observed antiproliferation properties, it was concluded that the function/group at the 3-position of the indole-2-carboxamide is the dominant factor, with the bio-properties following the order of H > methoxyvinyl > ethoxymethyl > hydroxymethyl > phenyl.^100^

**Scheme 2:**
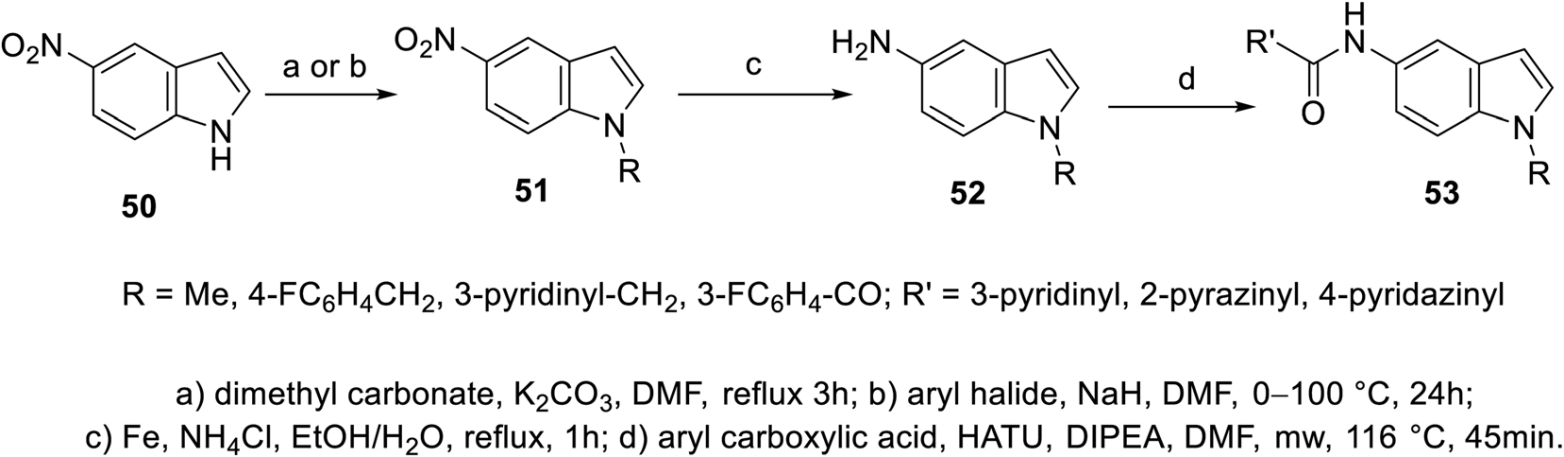
Synthesis of 5-indolecarboxamides 53.

A set of 5-indolecarboxamides 53 was obtained through the reaction of the corresponding amino analogs 52 with the appropriate carboxylic acid in the presence of Hünig's base (*N*,*N*-diisopropylethylamine “Hünig's base”, DIPEA) using HATU (hexafluorophosphate azabenzotriazole tetramethyl uronium, coupling agent). 5-Aminoindoles 52 were prepared through the reduction of nitro analogs 51 (Fe, NH_4_Cl, EtOH/H_2_O). The latter 51 was obtained through the alkylation of 5-nitroindole 50 ^101,102^ (Scheme 2). Weak VEGFR-2, CDK-1/cyclin B (cyclin-dependent kinase 1), and HER-2 (human epidermal growth factor receptor 2) properties were exhibited by most of the synthesized agents 53 at 10 μM. However, compound 53l [R = CO(3-FC_6_H_4_), R′ = 4-pyridazinyl] revealed promising kinase inhibitory activity against CDK-1/cyclin B and HER-2 (activity = 51% and 52%, respectively) at 10 μM ^101^ (ESI Fig. S2†).

### 4.2. Indolyl Schiff bases

**Scheme 3:**
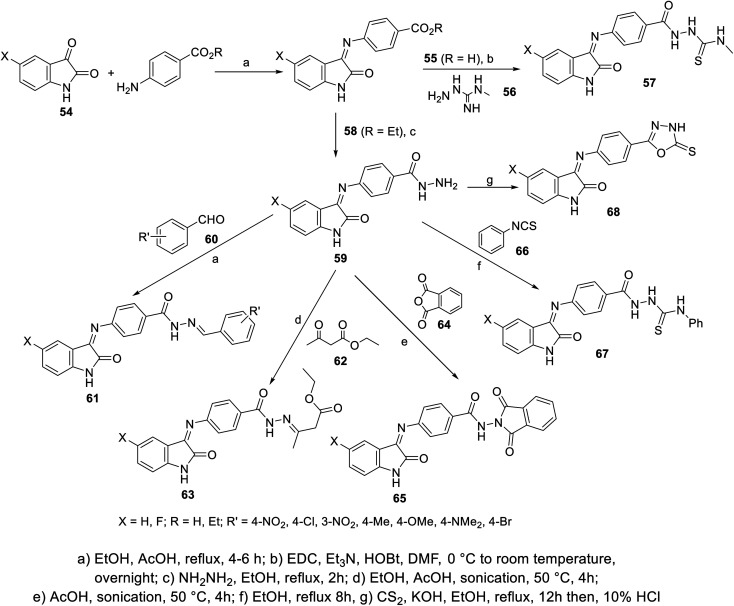
Synthesis of indolyl Schiff bases 55, 57–59, 61, 63, 65, 67 and 68.

Condensation of isatins 54 with *p*-aminobenzoic acid (in refluxing EtOH containing a catalytic amount of AcOH) resulted in the formation of Schiff bases 55, which upon reaction with 4-methylthiosemicarbazide 56 afforded the corresponding semicarbazones 57. Similarly, the reaction of isatins 54 with benzocaine (4-aminobenzoic acid ethyl ester) 58, followed by reaction with hydrazine hydrate in refluxing ethanol gave the corresponding hydrazides 59. The latter were subjected to reaction with aromatic aldehydes 60 (in refluxing EtOH containing a catalytic amount of AcOH), ethyl acetoacetate 62 (in EtOH/AcOH, under sonication at 50 °C), phthalic anhydride 64 (in AcOH “glacial” under sonication at 50 °C) or phenyl isothiocyanate 66 (in refluxing EtOH), yielding the corresponding hydrazone 61, 63, dioxoisoindoline 65 and thiourea derivatives 67, respectively. Reaction of 59 with carbon disulfide in refluxing ethanolic KOH followed by acidification with HCl (10%), afforded the corresponding oxadiazolyl derivatives 68 (Scheme 3).

Some of the synthesized agents exhibited promising antiproliferation properties against HepG2 (liver) and MCF7 (breast) cancer cell lines relative to that of sunitinib (MTT assay; IC_50_, μM ± SD “standard division”) (ESI Fig. S3†). Some of the synthesized fluorinated analogs showed enhanced antitumor properties compared to other prepared derivatives, directing attention on the importance of this substituent in controlling the bio-properties. The VEGFR-2 inhibitory properties determined for the discovered promising agents were consistent with the observed antitumor properties. Compound 67a (X = H) revealed promising antiproliferation and enzymatic inhibitory properties (IC_50_ = 1.13 ± 0.06, 1.44 ± 0.11 μM against HepG2 and MCF7, respectively; IC_50_ = 0.078 ± 0.003 μM against VEGFR-2) relative to that of sunitinib (IC_50_ = 2.23 ± 0.11, 4.77 ± 0.29 μM against HepG2 and MCF7, respectively; IC_50_ = 0.139 ± 0.007 μM against VEGFR-2)^103^ (ESI Fig. S3†).

**Scheme 4:**
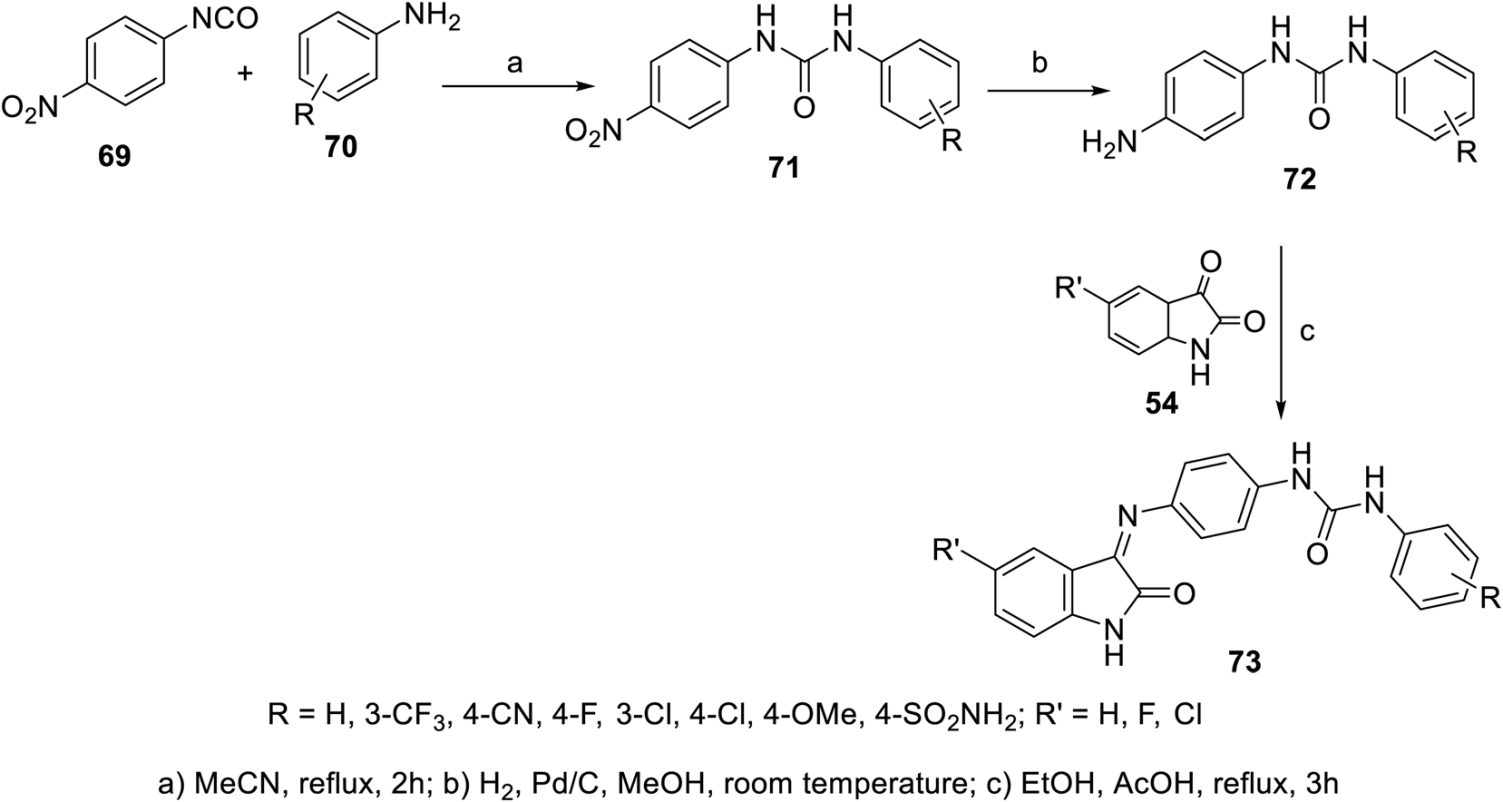
Synthesis of indolyl Schiff bases 73.

A set of indolyl Schiff bases incorporated in urea 73 was synthesized through the condensation reaction of the appropriate indoles 54 with the corresponding 1-(4-aminophenyl)-3-substituted urea 72. The latter was obtained through reduction (H_2_, Pd/C, and MeOH) of the corresponding nitro analogs, which were prepared through reaction of 4-nirophenylisocyanate 69 with the appropriate anilines 70 in refluxing acetonitrile^104^ (Scheme 4). Some of the synthesized Schiff bases revealed promising antiproliferation properties (SRB “sulforhodamine B” technique) against the HepG2 (liver) cancer cell line relative to that of doxorubicin and sorafenib. The VEGFR-2 properties were determined for the discovered promising agents, which showed comparable observations to that of the antiproliferation efficacies. The efficacy observed for most of the synthesized agents followed order of phenyl substitution of 4-Cl > 3-Cl > 3-CF_3_. The most promising agent discovered was 73x (R = SO_2_NH_2_, R′ = Cl; IC_50_ = 3.15 ± 0.36 and 0.31 ± 0.04 μM for HepG2 cell line and VEGFR-2, respectively), which exhibited comparable activity to that of sorafenib (IC_50_ = 3.40 ± 0.25, 0.10 ± 0.02 μM for the HepG2 cell line and VEGFR-2, respectively)^104^ (ESI Fig. S4†).

**Scheme 5:**
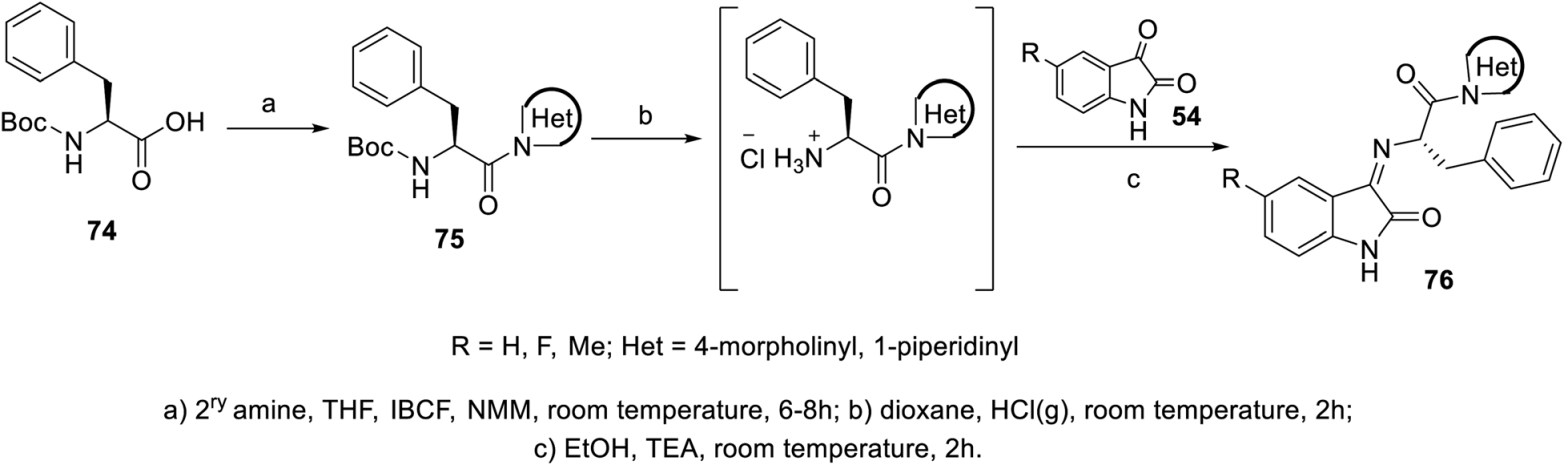
Synthesis of indolyl Schiff bases 76.

**Scheme 6:**
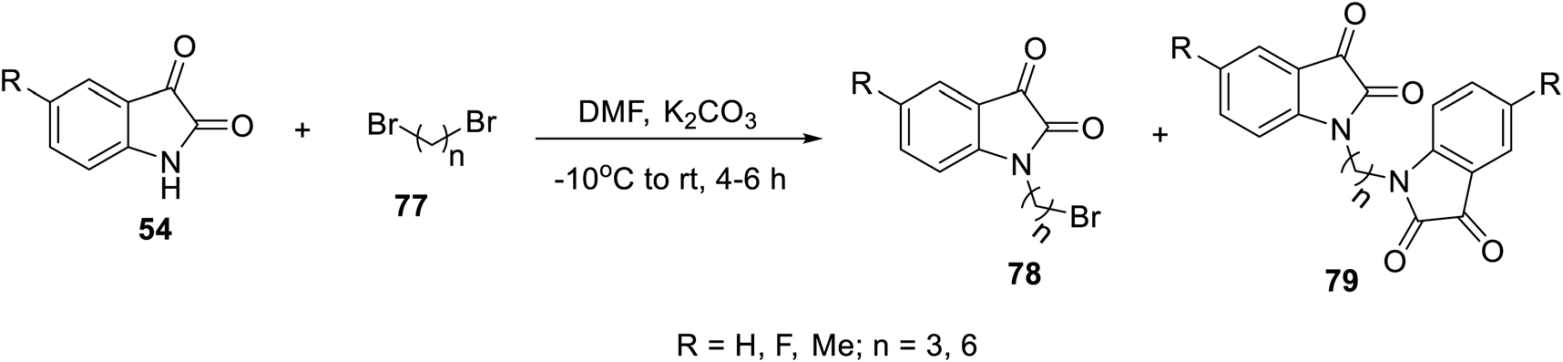
Synthesis of mono- 78 and bis-alkylated isatins 79.

**Scheme 7:**
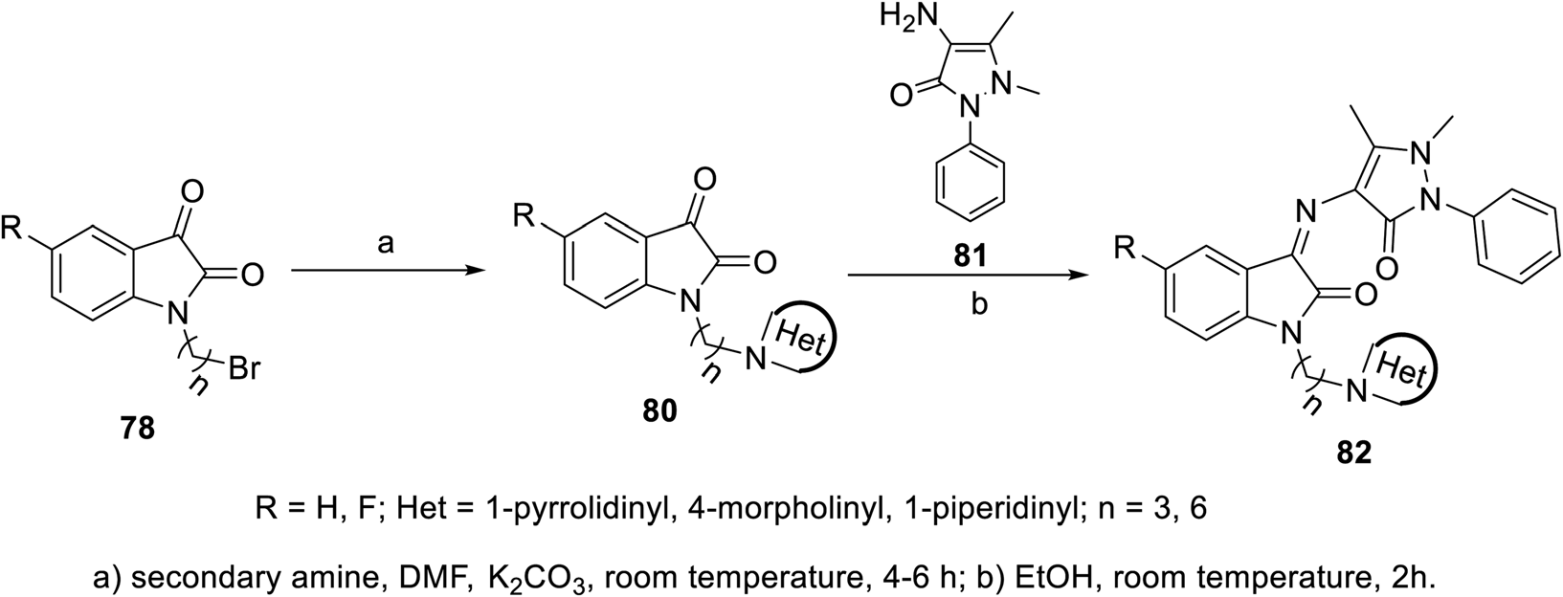
Synthesis of indolyl Schiff bases 82.

**Scheme 8:**
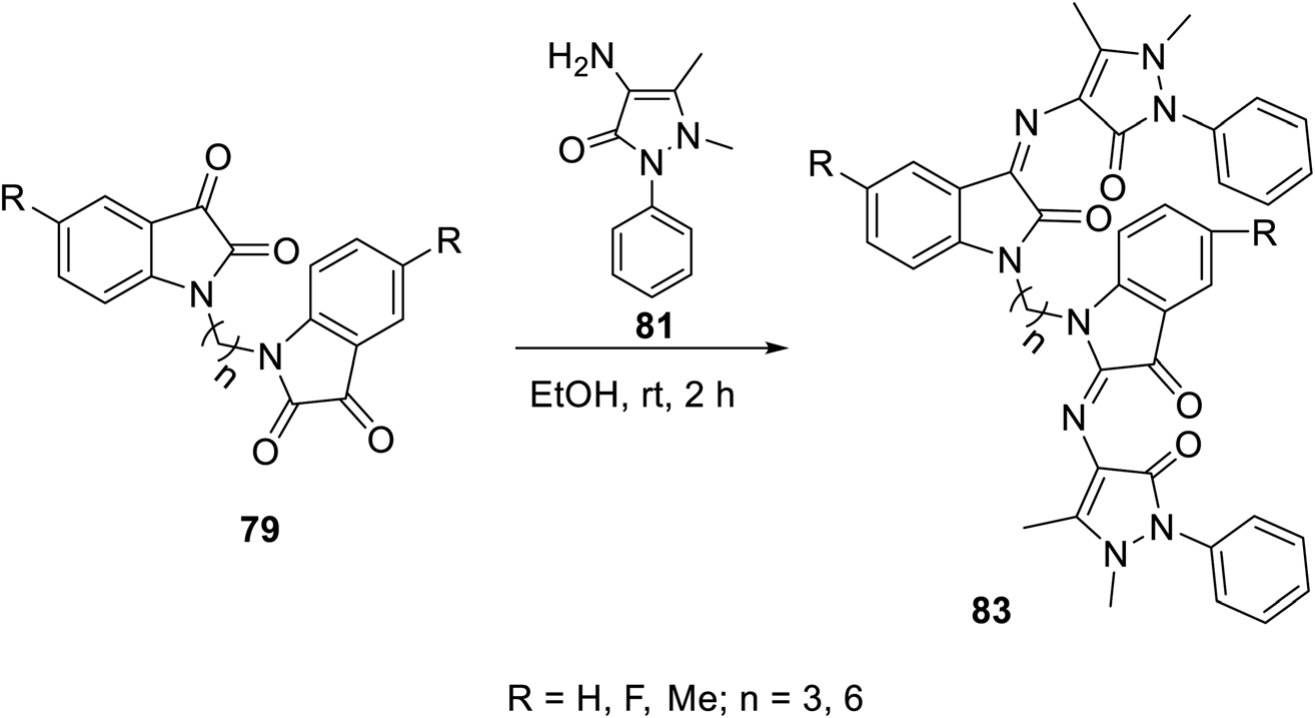
Synthesis of indolyl Schiff bases 83.

The condensation reaction of isatins 54 with l-phenylalanine secondary amine conjugates (obtained from the reaction of Boc amino acid with secondary amines in THF (tetrahydrofuran) in the presence of IBCF (iso-butyl chloroformate) and NMM (*N*-methyl morpholine) at room temperature and inert atmosphere followed by removal of the Boc group (HCl_gas_ in dioxane)) in EtOH containing triethylamine (TEA) at room temperature gave the corresponding Schiff bases 76 (Scheme 5). Additionally, the reaction of isatins 79/80 with 4-amino antipyrine 81 in ethanolic solution at room temperature afforded the corresponding Schiff bases 83 and 82, respectively. Isatin derivatives 80 were obtained through alkylation with excess dibromoalkane (DMF/K_2_CO_3_), which gave monoalkylated derivatives 78 (major products) and bis-isatin derivatives 79 (minor products). The monoalkylated isatins were coupled with secondary amines (DMF/K_2_CO_3_ at room temperature) affording the corresponding isatin derivatives 80 (Schemes 6–8).

**Scheme 9:**
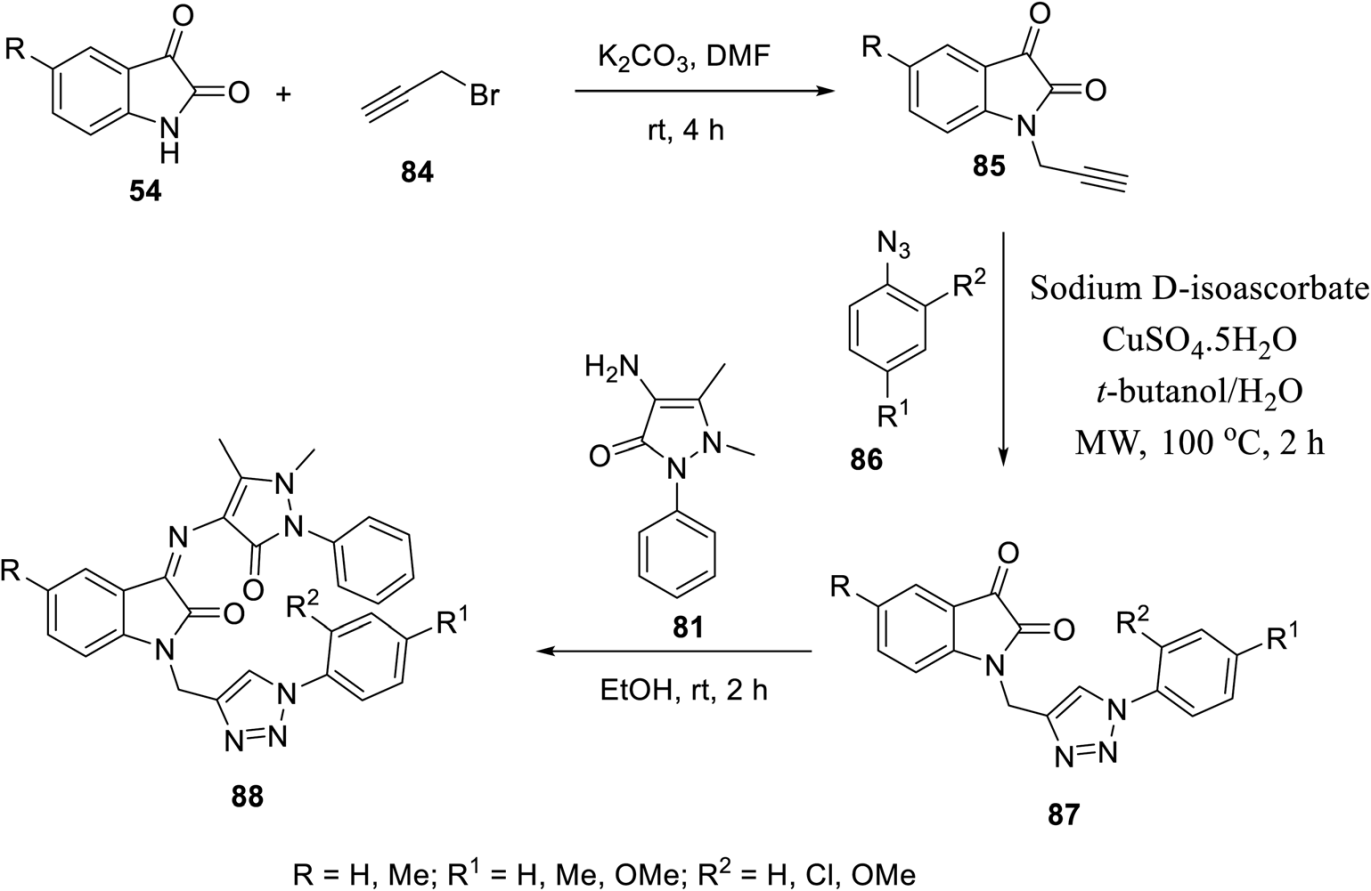
Synthesis of indolyl Schiff bases 88.

Similarly, Schiff bases 88 were obtained through the condensation of 4-amino antipyrine 81 with isatin triazol conjugates 87 (EtOH, room temperature). Isatin triazol conjugates 87 were synthesized through the click reaction of the appropriate aryl azides 86 with *N*-propargylated isatins 85 (*t*-butanol/H_2_O, CuSO_5_·5H_2_O, sodium d-isoascorbate, microwave, 100 °C)^15^ (Scheme 9).

Some of the synthesized Schiff bases exhibited promising antiproliferation properties against MCF7 (breast), HCT116 (colon) and PaCa2 (pancreatic) cancer cell lines (MTT assay) compared to the reference standards (sunitinib and 5-fluorouracil) (ESI Fig. S5†). Compound 88f (R = Me, R^1^ = H, and R^2^ = OMe) was the highest potent analog observed against MCF7 (2.1-times potency relative to the standard reference sunitinib). Additionally, some of the compounds prepared (88b, 88d and 88f) exhibited higher efficacies than that of the standard drug 5-flurouracil (approved drug for colon cancer^105^). The safe profile of all the tested analogs (IC_50_ = >50.00 μM) against the non-cancer RPE1 cell line is good support, especially for the high potent analogs towards more detailed studies for assigning promising hits. The CAM assay (chick chorioallantoic membrane) of fertilized chicken eggs in addition to VEGFR-2 inhibitory properties (% inhibition ± SD utilizing the IC_50_ values observed against MCF7 cell line of the tested agents relative to that of sunitinib) (ESI Fig. S5†) support their capability towards antiangiogenesis.^15^

### 4.3. Indolyl hydrazones

**Scheme 10:**
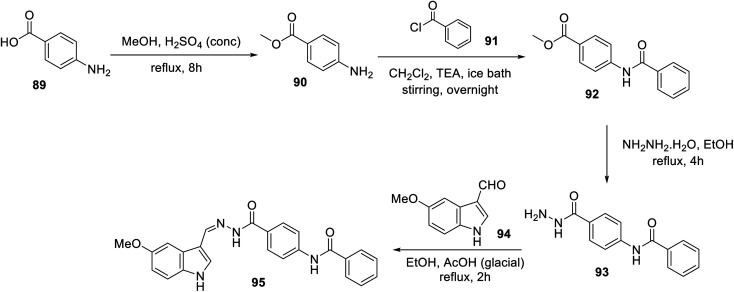
Synthesis of hydrazone derivative 95.

An ethanolic solution of 3-indolecarbaldehyde derivative 94 and hydrazide analog 93 in the presence of a catalytic amount of acetic acid under reflux afforded hydrazone 95 (Scheme 10). Hydrazone 95 exhibited considerably higher antiproliferation properties (MTT assay) against the MCF-7 and HCT116 cell lines (IC_50_ = 12.93 ± 0.54 and 11.52 ± 0.70 μM, respectively) compared to that of sorafenib (IC_50_ = 4.32 ± 0.33 and 7.28 ± 0.53 μM, respectively) with a safety profile against the W138 (non-cancer) cell line. Furthermore, the VEGFR-2 inhibitory properties of 95 (IC_50_ = 25 ± 1.29 nM) were comparable to that of the standard reference sorafenib (IC_50_ = 35 ± 1.34 nM). This study was supported by diverse computational studies (molecular docking “PDB: 2OH4, MOE2014 software”; MD “molecular dynamics, CHARMM-GUI web server, GROMACS 2021 engine”; DFT “density functional theory, Gaussian 09” and ADMET “absorption, distribution, metabolism, excretion and toxicity; Discovery Studio 4.0”).^106^

**Scheme 11:**
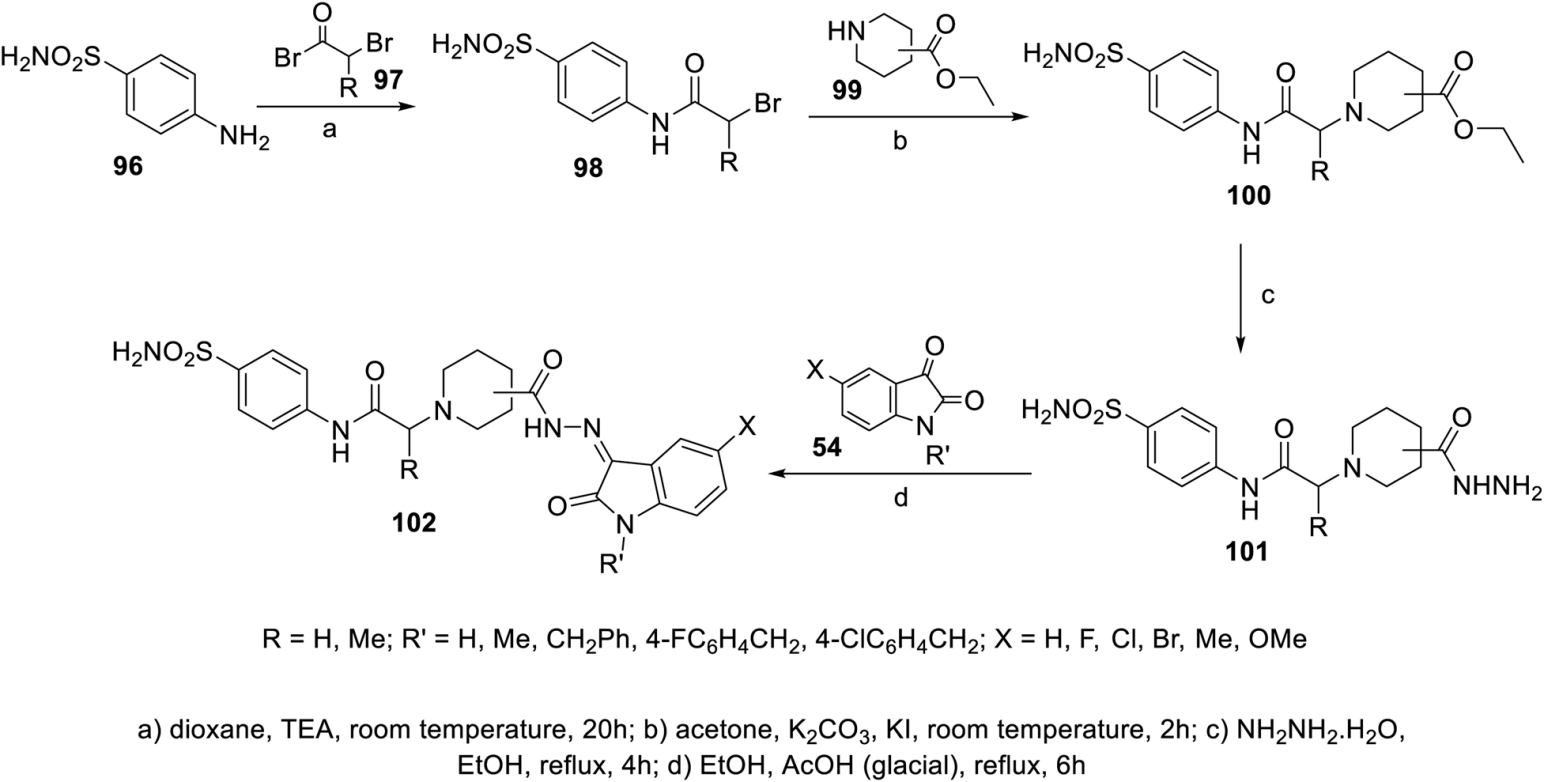
Synthesis of hydrazones 102.

**Fig. 9:**
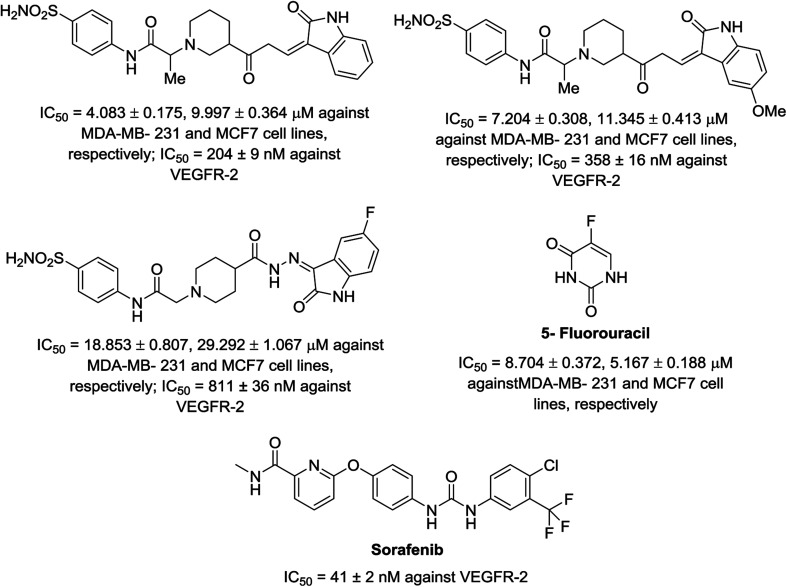
Antiproliferation (in μM ± SD) and inhibitory properties against VEGFR-2 (in μM ± SD) of the tested hydrazones 102 and standard references (5-fluorouracil and sorafenib), respectively.

A series of indolyl hydrazones incorporating benzenesulfonamides 102 was synthesized through the condensation of the appropriate isatins 54 with hydrazides 101 in refluxing ethanol containing a catalytic amount of acetic acid. Hydrazides 101 were obtained through the reaction of hydrazine hydrate with the corresponding ethyl esters 100 in refluxing ethanol (Scheme 11). Some of the synthesized hydrazones 102 revealed considerable antiproliferation activities against MDA-MB-231 and MCF7 (breast cancer cell lines) compared to that of 5-fluorouracil, and VEGFR-2 inhibitory properties compared to that of sorafenib (Fig. 9). Inhibitory properties against carbonic anhydrase (CA) *h*CAI, II, IX, XII were also observed by hydrazones 102 compared to the standard reference acetazolamide.^107^

**Scheme 12:**
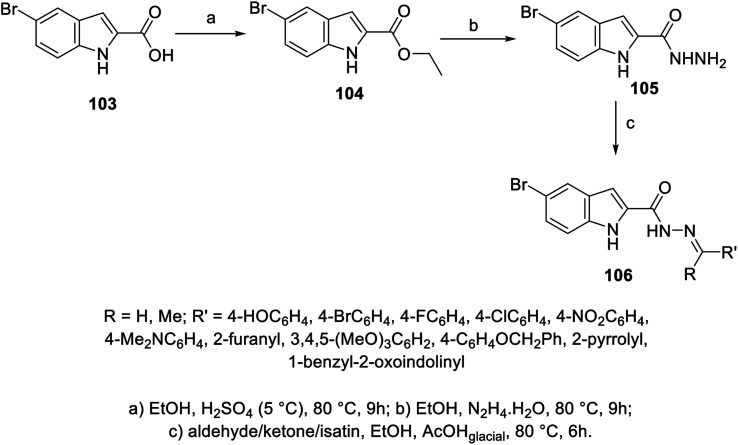
Synthesis of hydrazones 106.

A set of hydrazones 106a–k was prepared through the condensation reaction of 5-bromo-2-indolecarbohydrazide 105 (obtained from the reaction of hydrazine hydrate with ethyl ester of indole derivative 104 in refluxing EtOH) with the appropriate aldehyde, ketone or isatin derivative in refluxing EtOH containing a few drops of glacial AcOH (catalytic amount) (Scheme 12). Mild antiproliferation properties were exhibited by the synthesized hydrazones 106a–k against HepG2 (liver), HeLa (cervical) and PC3 (prostate) cancer cell lines (MTT assay) with modest VEGFR-2 inhibitory activity compared to the standard reference (sorafenib; IC_50_ = 6.2 ± 1.1, 11.7 ± 1.3, 19.0 ± 1.2, and 15.3 ± 1.8 μM against HepG2, HeLa, PC3 and WI-38 respectively; EC_50_ = 57.1 ± 3.0 nM ± SEM against VEGFR-2). Furthermore, the most promising agent 106e (R=4-Me_2_NC_6_H_4_, R′=H; IC_50_ = 14.3 ± 2.0, 22.2 ± 2.3, 36.2 ± 3.1, and 25.9 ± 2.1 μM against HepG2, HeLa, PC3 and WI-38, respectively) was screened against WI-38 (normal lung fibroblasts; EC_50_ = 102.6 ± 3.1 nM ± SEM against VEGFR-2) to confirm its safety index^108^ (ESI Fig. S6†).

**Scheme 13:**
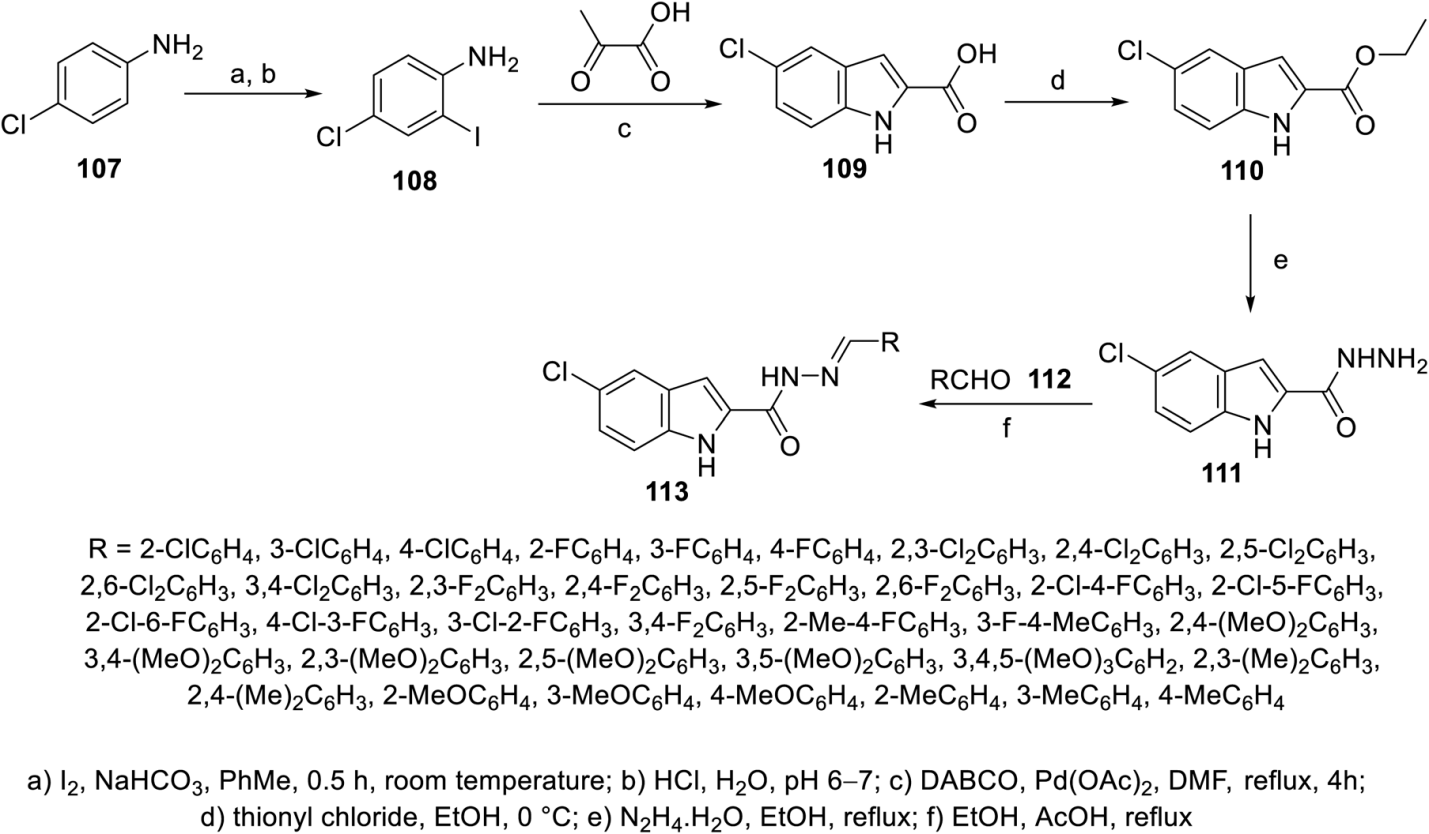
Synthesis of indolyl hydrazones 113.

Similarly, 5-chloroindolyl hydrazones 113 were obtained through the reaction of the corresponding hydrazide 111 and aromatic aldehydes 112 in refluxing ethanol containing AcOH as a catalyst (Scheme 13). Considerable antiproliferation properties against HCT116 and SW489 (colon) cancer in addition to MRC-5 (non-cancer human) cell lines were exhibited by some of the synthesized hydrazones compared to the standard references (cisplatin “GI_50_ = 7.67 ± 3.4, 4.43 ± 2.1, and 3.82 ± 1.9 μM against HCT116, SW480 and MRC-5, respectively”, sorafenib “GI_50_ = 4.17 ± 2.5, 2.02 ± 1.2, and 30.81 ± 10.6 μM against HCT116, SW480 and MRC-5, respectively” and sunitinib “GI_50_ = 15.84 ± 1.7, 1.09 ± 0.9, > 100 μM against HCT116, SW480 and MRC-5, respectively”) (ESI Fig. S7†). Compound 113x (R = 2,4-(MeO)_2_C_6_H_3_; GI_50_ = 8.10 ± 1.8, 7.90 ± 1.3, and >100 μM against HCT116, SW480 and MRC-5, respectively) was the most promising agent synthesized. Its anti-angiogenesis effect was supported by CAM proliferation and HUVEC (human umbilical vein endothelial cell) migration.^109^

### 4.4. 2-Oxoindolin-3-ylidenes

**Scheme 14:**
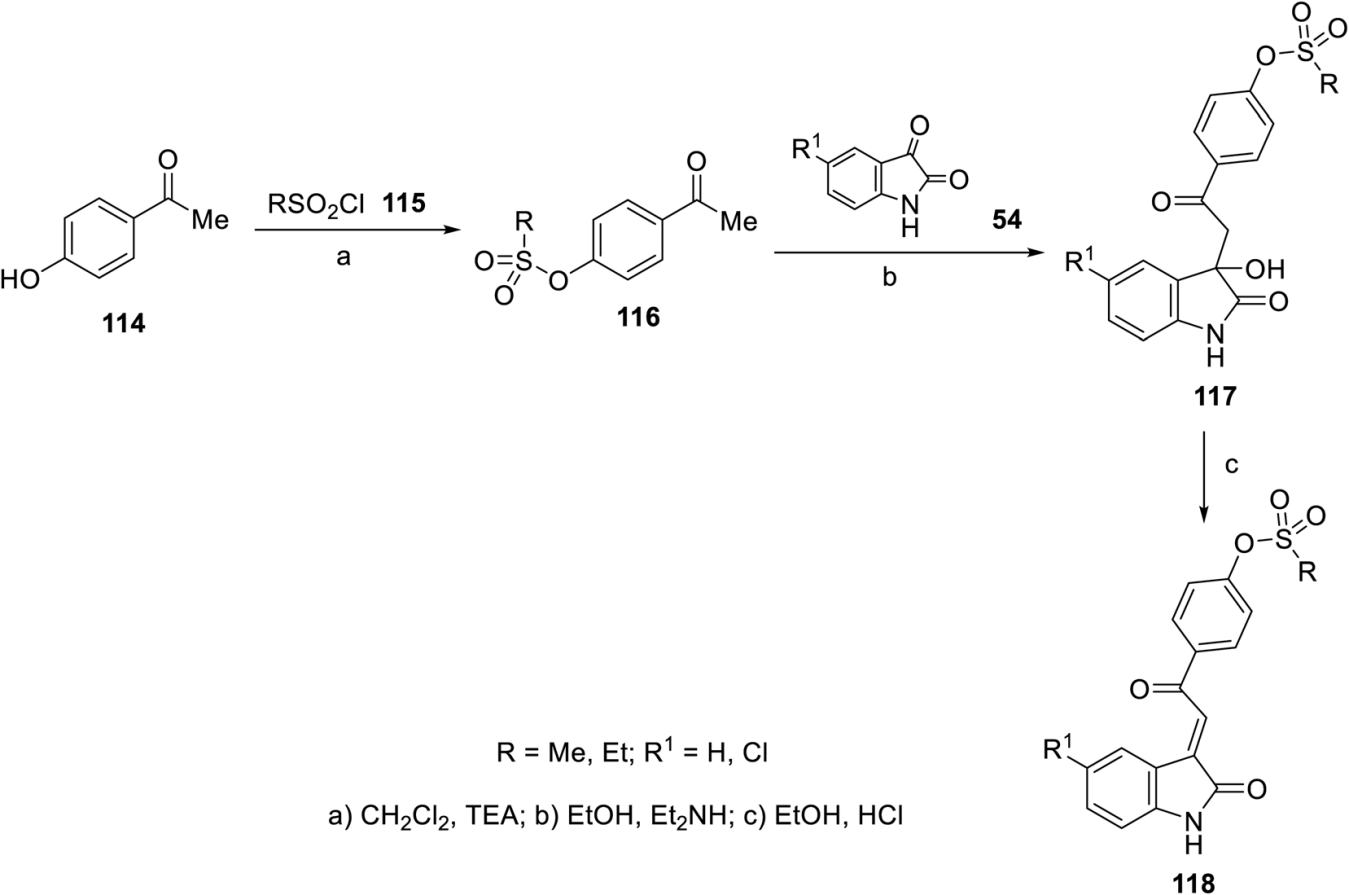
Synthesis of 2-oxoindolin-3-ylidenes 118.

The FDA approval for the use of sunitinib and nintedanib as potent multi-targeted tyrosine kinase inhibitors against many cancer types^40,41,55,56^ has inspired many researchers to utilize the 2-oxoindolin-3-ylidene scaffold for optimizing novel antitumor active agents. A variety of 2-oxoindolin-3-ylidenes bearing alkanesulfonate 118 was synthesized in a two-step reaction in excellent yields. The reaction of isatins 54 in EtOH containing a quantitative amount of Et_2_NH gave the corresponding 3-hydroxy-2-oxoindole derivatives 117. Acidic dehydration (EtOH and HCl) of 117 gave the corresponding 118 ^92^ (Scheme 14).

**Scheme 15:**
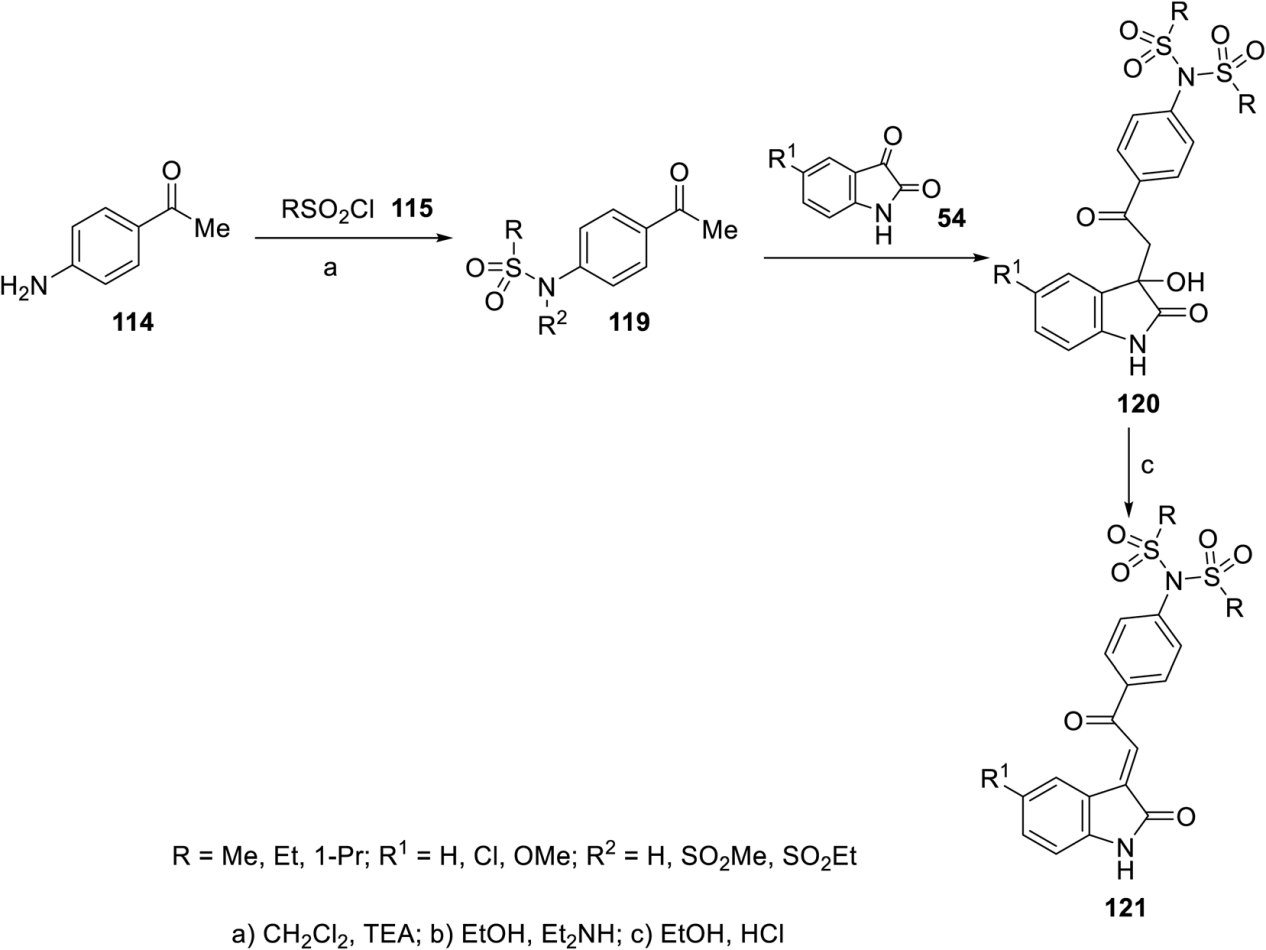
Synthesis of 2-oxoindolin-3-ylidenes 121.

**Scheme 16:**
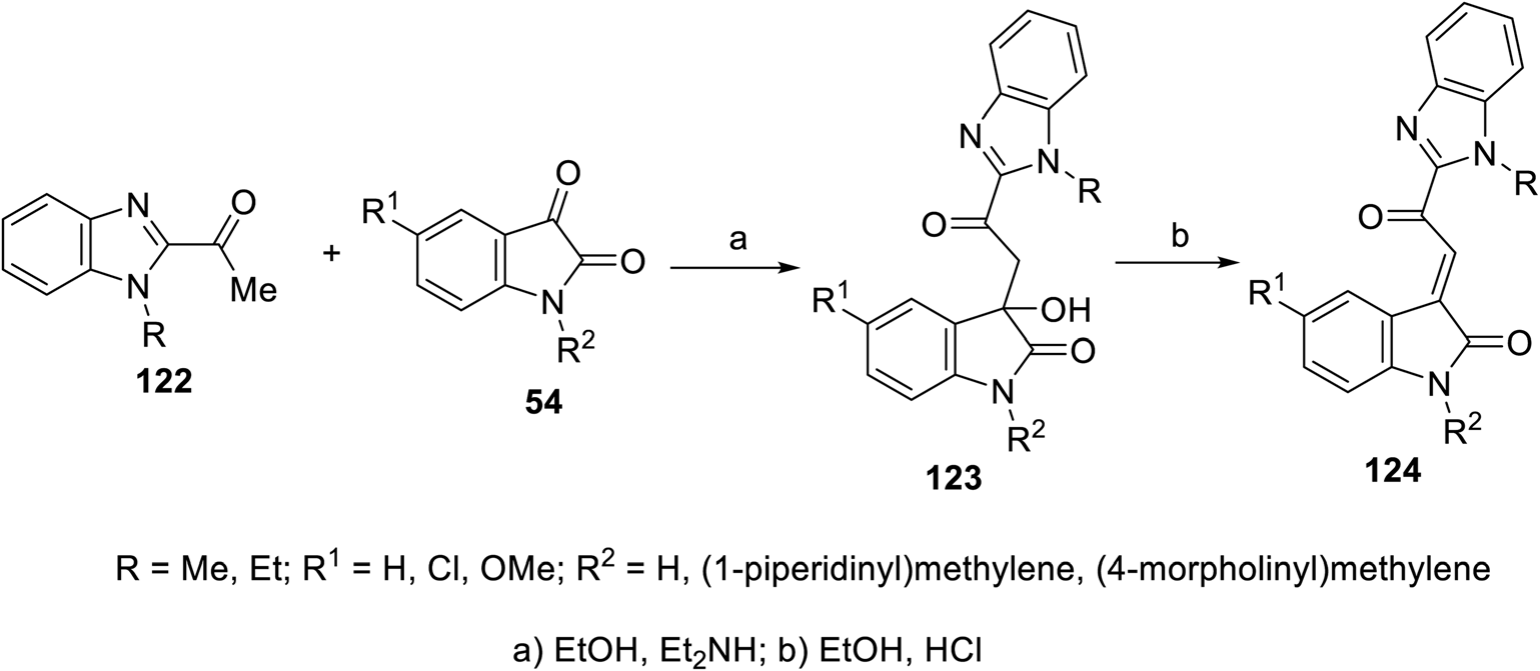
Synthesis of 2-oxoindolin-3-ylidenes 124.

2-Oxoindolin-3-ylidenes incorporating alkanesulfonamide 121 were formed starting from the corresponding alkanesulfonamide derivatives 119 utilizing a similar reaction sequence. It was mentioned that the reaction of isatins 54 with 119 bearing a mono-propanesulfonamide function gave the corresponding 120. This is presumably *via* the elimination of propylsulfonate from 119 under the applied basic condition^92^ (Scheme 15). Similarly, 2-oxoindolin-3-ylidenes connected to benzimidazolyl heterocycle 124 were prepared following the same reaction sequence utilizing 2-acetylbenimidazoles 122 ^92^ (Scheme 16).

**Fig. 10:**
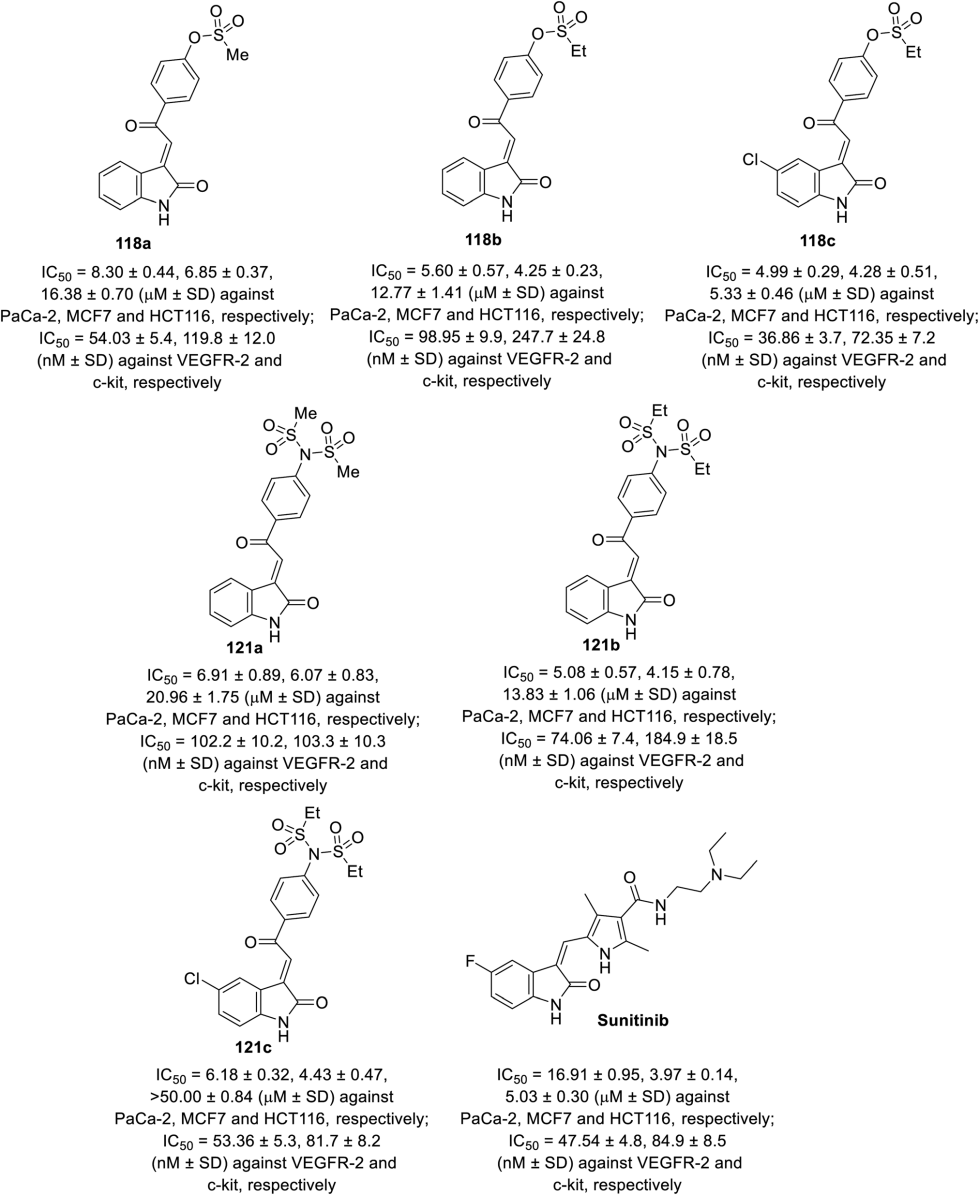
Antiproliferation and tyrosine kinase (VEGFR-2, c-kir) inhibitory properties of 118a–c, 121a–c and sunitinib.

Some of the synthesized agents 118 and 121 exhibited antiproliferation properties (MTT assay) against PaCa-2 (pancreatic), MCF7 (breast) and HCT116 (colon) cancer cell lines with potency comparable to that of sunitinib. The safety index of the tested agents was established through screening against the RPE1 (retinal pigment epithelium) normal cell line. The inhibitory properties of the tested agents against VEGFR-2 and c-kit were comparable to that of the antiproliferation results (Fig. 10). Their anti-angiogenesis properties were also supported by the CAM assay.^92^

**Scheme 17:**
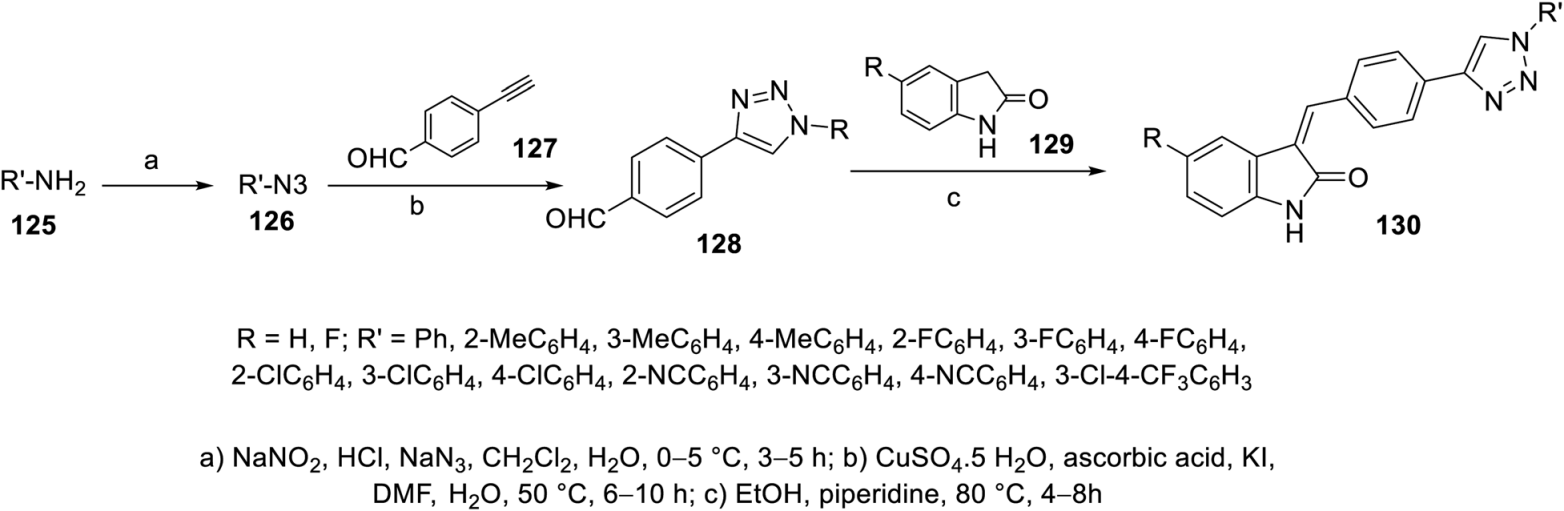
Synthesis of 2-oxoindolin-3-ylidenes 130.

Condensation of 2-indolinones 129 with aryl aldehydes substituted with 1,2,3-triazolyl heterocycle 128 in refluxing ethanol containing a catalytic amount of piperidine gave the corresponding 2-oxoindolin-3-ylidenes 130. The aryl aldehydes bearing 1,2,3-triazolyl heterocycle 128 were obtained through the click reaction of 4-ethynylbenzaldehyde 127 and azidobenzene 126 in DMF containing aqueous CuSO_4_·5H_2_O, ascorbic acid and a catalytic amount of KI^110^ (Scheme 17). Based on the VEGFR-2 inhibitory properties of the tested 130, it was observed that the substituent of the phenyl ring attached to the triazolyl nitrogen possesses a significant effect on the observed bio-properties. The potency against VEGFR-2 followed the order of 4-CH_3_ > 3-CH_3_ > 2-CH_3_ > H. The antiproliferation properties (CCK-8 assay) were studied against HT-29 (colon), MKN-45 (gastric) and HUVEC (umbilical vein endothelial) cancer cells. Compound 130d (R = H, R′ = 4-MeCH_4_; IC_50_ = 1.61 ± 0.45, 1.92 ± 0.37, and 7.94 ± 0.36 μM against HT-29, MKN-45 and HUVEC, respectively; IC_50_ = 26.38 ± 1.09 nM against VEGFR-2) was the most promising agent synthesized with antiproliferation and anti-VEGFR-2 properties comparable to that of sunitinib (IC_50_ = 10.34 ± 0.96, 9.25 ± 0.77, and 6.37 ± 0.59 μM against HT-29, MKN-45 and HUVEC, respectively; IC_50_ = 83.20 ± 1.36 nM against VEGFR-2) (ESI Fig. S8†). Also, its angiogenesis properties were supported by a zebrafish labeling model assay.^110^

**Scheme 18:**
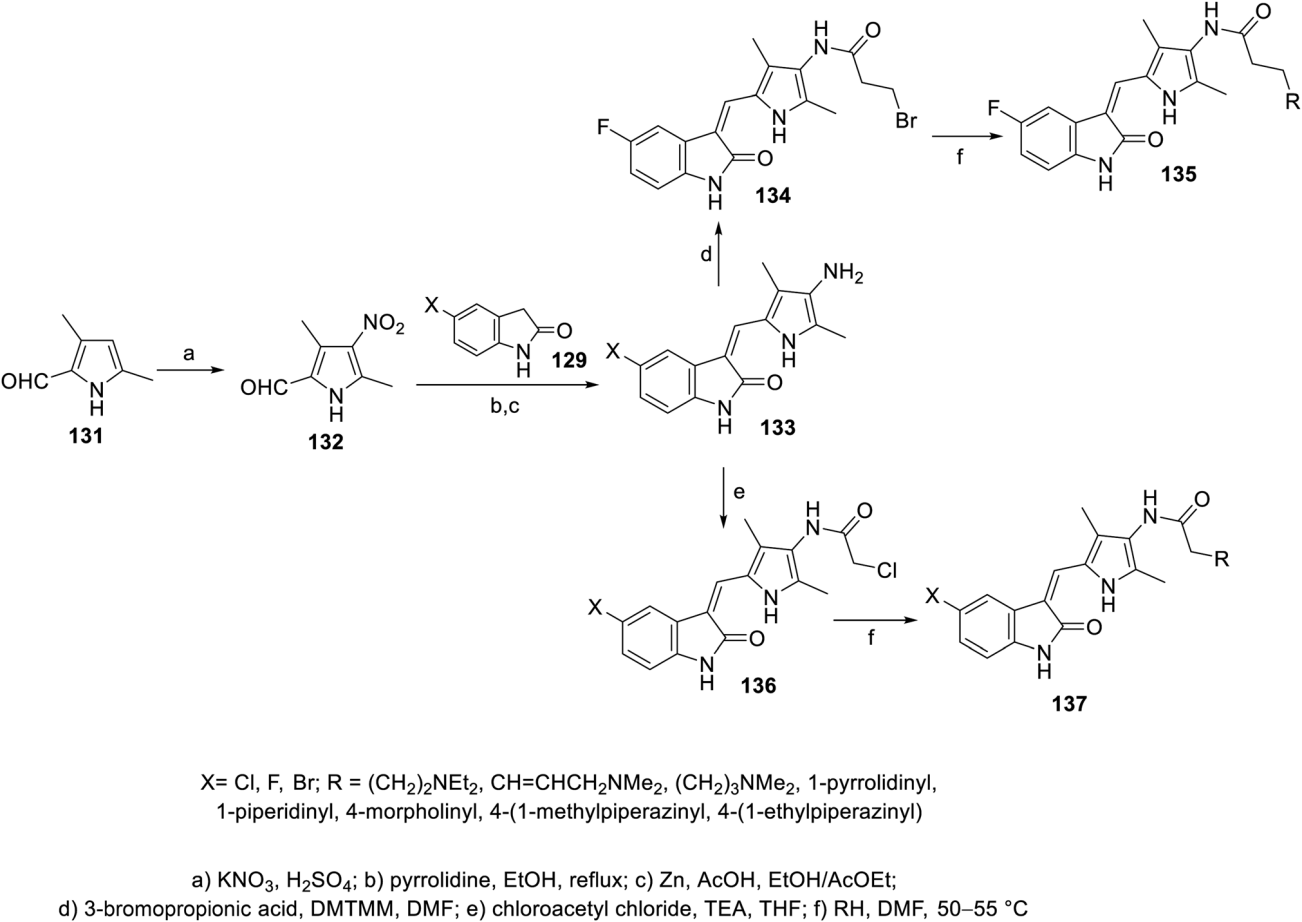
Synthesis of 2-oxoindolin-3-ylidenes 135 and 137.

**Fig. 11:**
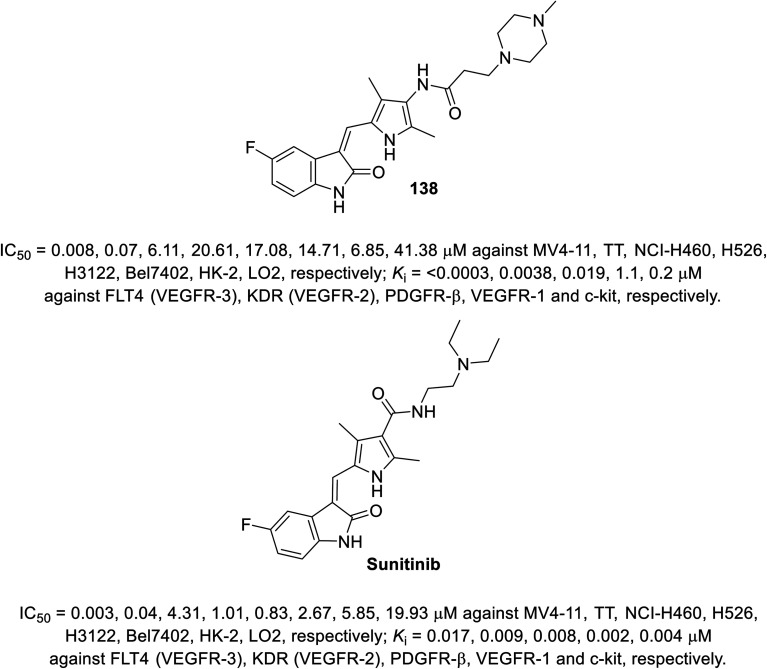
Antiproliferation and receptor inhibitory properties of 138 and sunitinib.

A series of 2-oxoindolin-3-ylidenes connected to pyrrole heterocycle 135 and 137 with high structural resemblance to sunitinib was synthesized *via* the condensation of 2-indolinones 129 with 2-pyrrolecarbaldehyde 132, followed by the reduction of the nitro group (Zn/AcOH). Subsequently, the reaction of 133 with chloroacetyl chloride (TEA, THF) or 3-bromopropionic acid (DMTMM: 4-(4,6-dimethoxytriazine)chlorinated-4-methylmorpholine, DMF) with various amines finally furnished 135 and 137 (Scheme 18). Based on their physicochemical properties and rat aortic ring assay, a few of the synthesized analogs was selected for *in vitro* testing (MTT assay) considering HT-29 (colon) and NCI-H460 (non-small cell lung) cancer cell lines, and compound 138 was selected for more sophisticated cell line and targeted receptor assays (Fig. 11) Compound 138 was nominated as a promising drug candidate based on its observed bio-properties compared to that of sunitinib.^39^

**Scheme 19:**
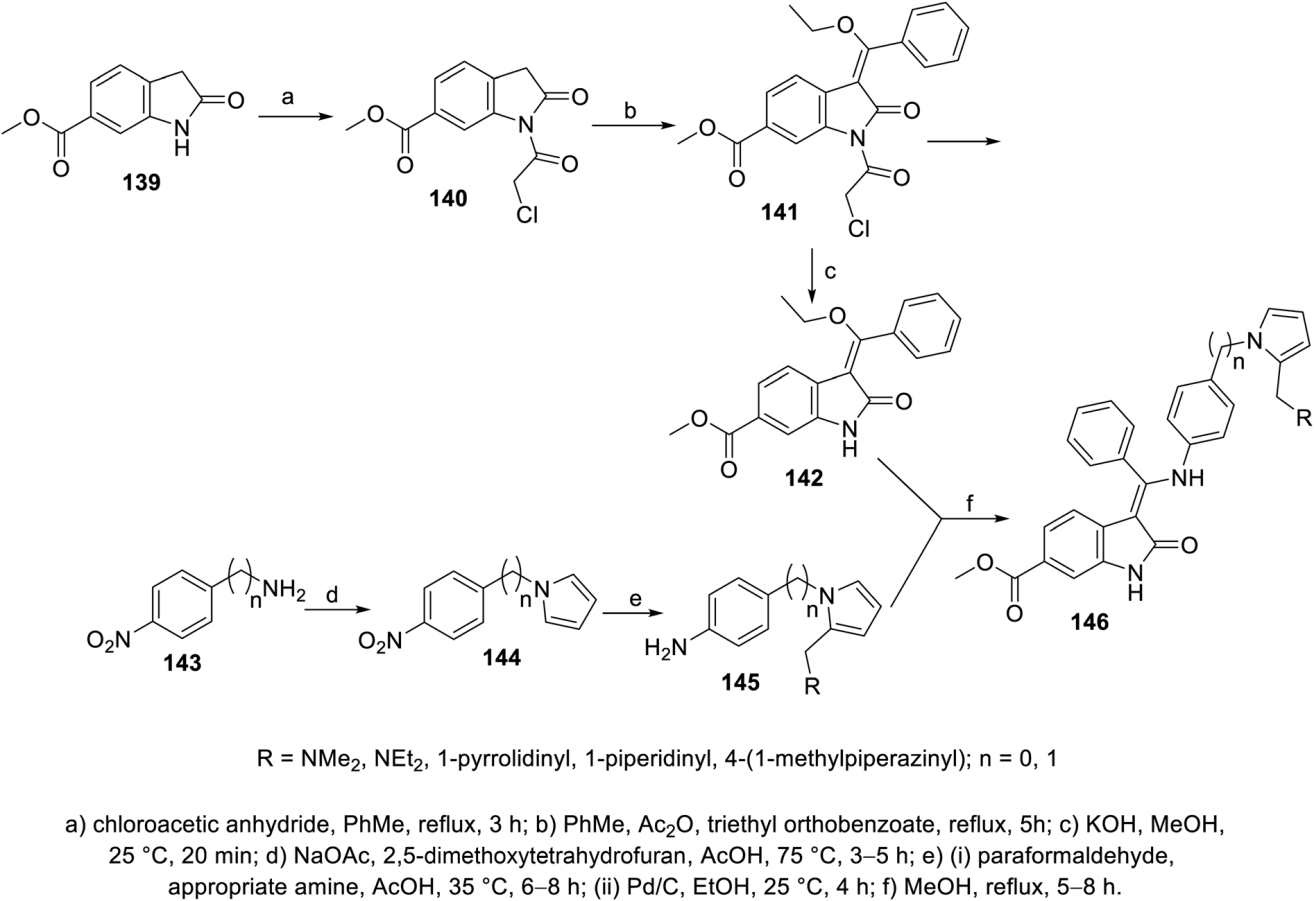
Synthesis of 2-oxoindolin-3-ylidenes 146.

2-Oxoindolin-3-ylidene 141 was obtained through the condensation of 2-indolinone 140 with triethyl orthobenzoate in boiling toluene (110 °C) in the presence of acetic anhydride. The removal of the chloroacetyl group (KOH, MeOH, room temperature), followed by reaction with 4-(1*H*-pyrrol-1-yl)aniline derivatives 145 gave 2-oxoindolin-3-ylidene 146 (Scheme 19). Some of the synthesized agents 146a (R=NMe_2_) and 146e (R = 1-methyl-4-piperazinyl) exhibited considerable VEGFR-2 and PDGFR-β inhibitory properties compared to that of nintedanib. The most promising agents observed were screened for antiproliferation properties (MTT assay) against HT-29 (colon), SK-OV-3 (ovarian) and HeLa (cervical) cancer cells, exhibiting promising activities (146a; IC_50_ = 51.7, 14.3 nM against VEGFR-2 and PDGFR-β, respectively; IC_50_ = 0.98 ± 0.11, 5.22 ± 0.36, 53.25 ± 1.20 μM against HT-29, SK-OV-3 and HeLa cells, respectively and 146e; IC_50_ = 38.0, 83.17 nM against VEGFR-2 and PDGFR-β, respectively; IC_50_ = 3.12 ± 0.27, 25.87 ± 1.32, 30.42 ± 1.98 μM against HT-29, SK-OV-3 and HeLa cells, respectively) compared to that of nintedanib (IC_50_ = 3.3, 3.7 nM against VEGFR-2 and PDGFR-β, respectively; IC_50_ = 4.90 ± 0.65, 28.76 ± 2.13, 51.65 ± 2.68 μM against HT-29, SK-OV-3 and HeLa cells, respectively)^111^ (ESI Fig. S9†).

**Scheme 20:**
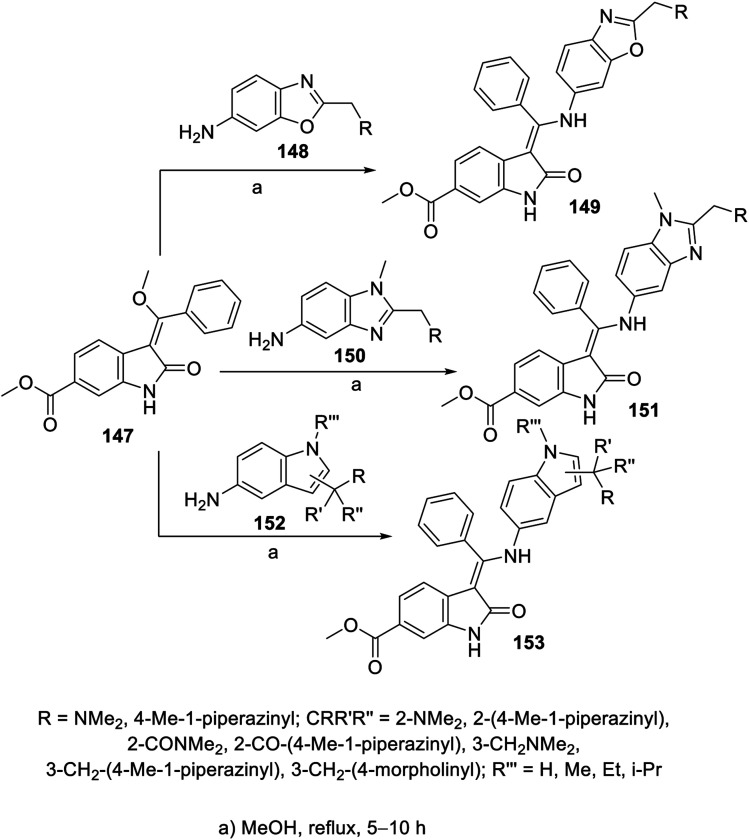
Synthesis of 2-oxoindolin-3-ylidenes 149, 151 and 153.

Similarly, the reaction of 2-oxoindolin-3-ylidene 147 with various amine-containing heterocycles (benzoxazole 148, benzimidazole 150 and indole 152) in refluxing methanol yielded the corresponding 2-oxoindolin-3-ylidenes 149, 151 and 153, respectively (Scheme 20). Some of the synthesized agents revealed promising antiproliferation activity (MTT assay) against the A549 (NSCLC), MCF7 (breast) and HT-29 (colon) cancer cell lines with enzymatic inhibitory properties against VEGFR-2 and PDGFR-β (ESI Fig. S10†). It was noticed that the prepared benzoxazolyl analogs were not tolerated due to either their weak or complete inactivity towards the tested enzymes. Meanwhile, the synthesized compounds with indolyl heterocycle 153 exhibited considerable antiproliferation properties against the MCF7 and/or HT-29 cell lines. Compound 153c (CR′R′′R′′′ = 2-[4-methylpiperazin-1-yl)carbonyl] was the most effective agent synthesized with promising inhibitory properties against VEGFR-2, -3 and PDGFR-α, PDGFR-β (IC_50_ = 69.1, 18.2, 4.4 and 22.0 nM, respectively) compared to nintedanib (IC_50_ = 8.5, 3.2, 2.3 and 3.5 nM, respectively). Furthermore, the safe proliferation behavior of 153c against the HEK293T (human embryonic kidney) normal cell line supports its use as a promising candidate.^112^

**Scheme 21:**
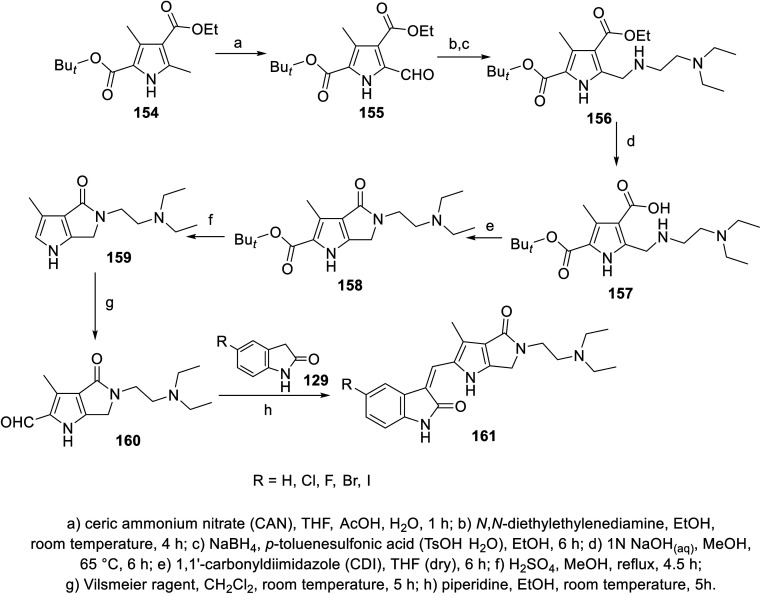
Synthesis of 2-oxoindolin-3-ylidenes 161.

**Fig. 12:**
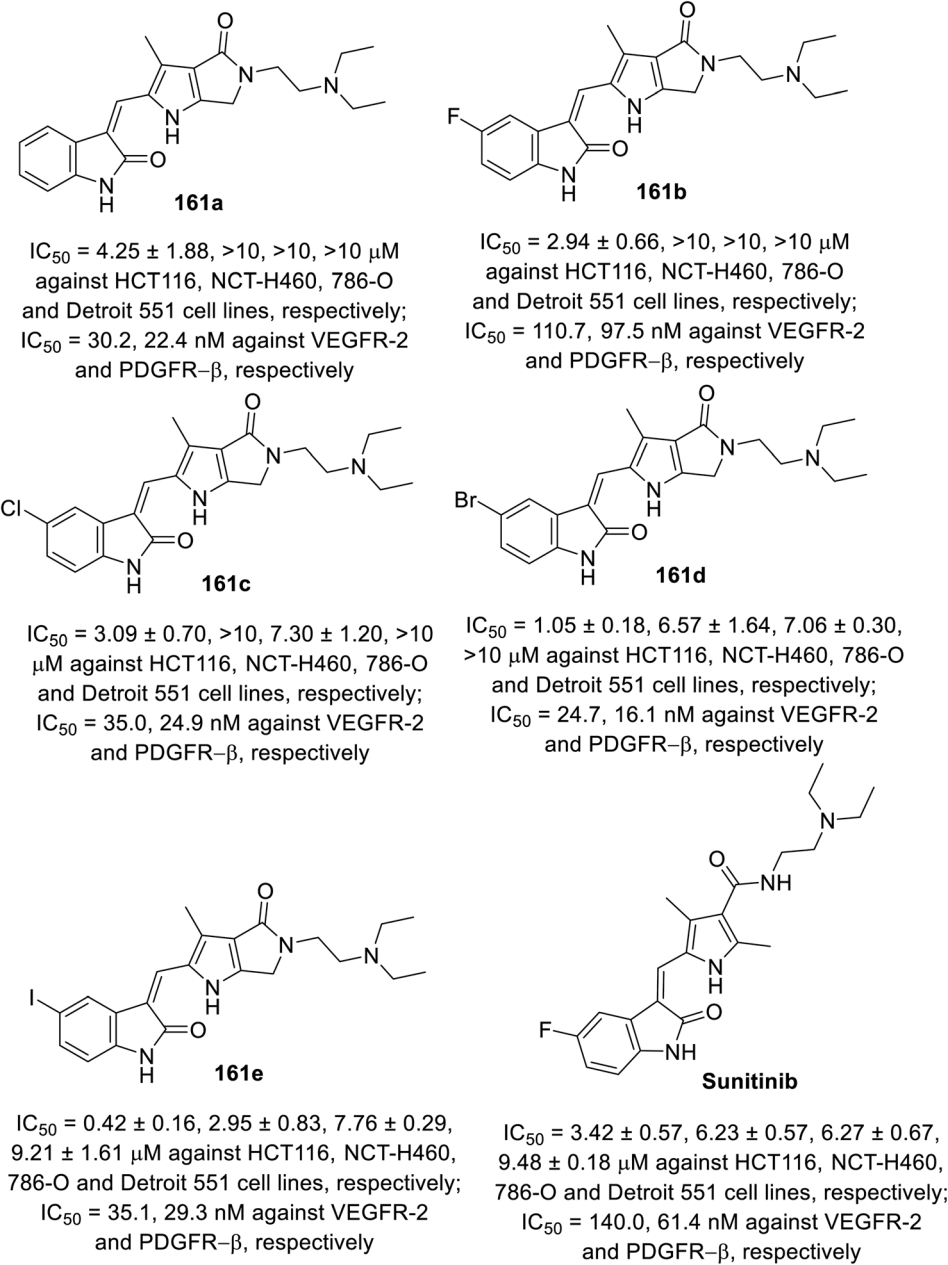
Antiproliferation and enzymatic inhibitory properties of 161a–e and sunitinib.

2-Oxoindolin-3-ylidenes linked to pyrrolo[3,4-*b*]pyrrol-2-yl 161 were synthesized through the condensation of 2-indolinones 129 with pyrrolo[3,4-*b*]pyrrole-2-carbaldehyde 160 (Scheme 21). The synthesized agents were screened for their antiproliferation properties (MTT assay) against HCT116 (colon), NCT-H460 (NSCLC), and 786-O (renal) cancer in addition to Detroit 551 (fibroblast) normal cell line. Iodoindolinone analog 161e (R = I) exhibited promising properties against HCT116 and NCT-H460 with potency higher than that of sunitinib (IC_50_ = 0.42 ± 0.16, 2.95 ± 0.83; 3.42 ± 0.57, 6.23 ± 0.57 μM for 161e and sunitinib, respectively). Bromo- 161d and iodoindolinones 161e showed considerable inhibitory properties against VEGFR-2 and PDGFR-β compared to that of sunitinib (IC_50_ = 24.7, 16.1; 35.1, 29.3; and 140.0, 61.4 nM against VEGFR-2 and PDGFR-β for 161d and 161e and sunitinib, respectively)^113^ (Fig. 12).

**Scheme 22:**
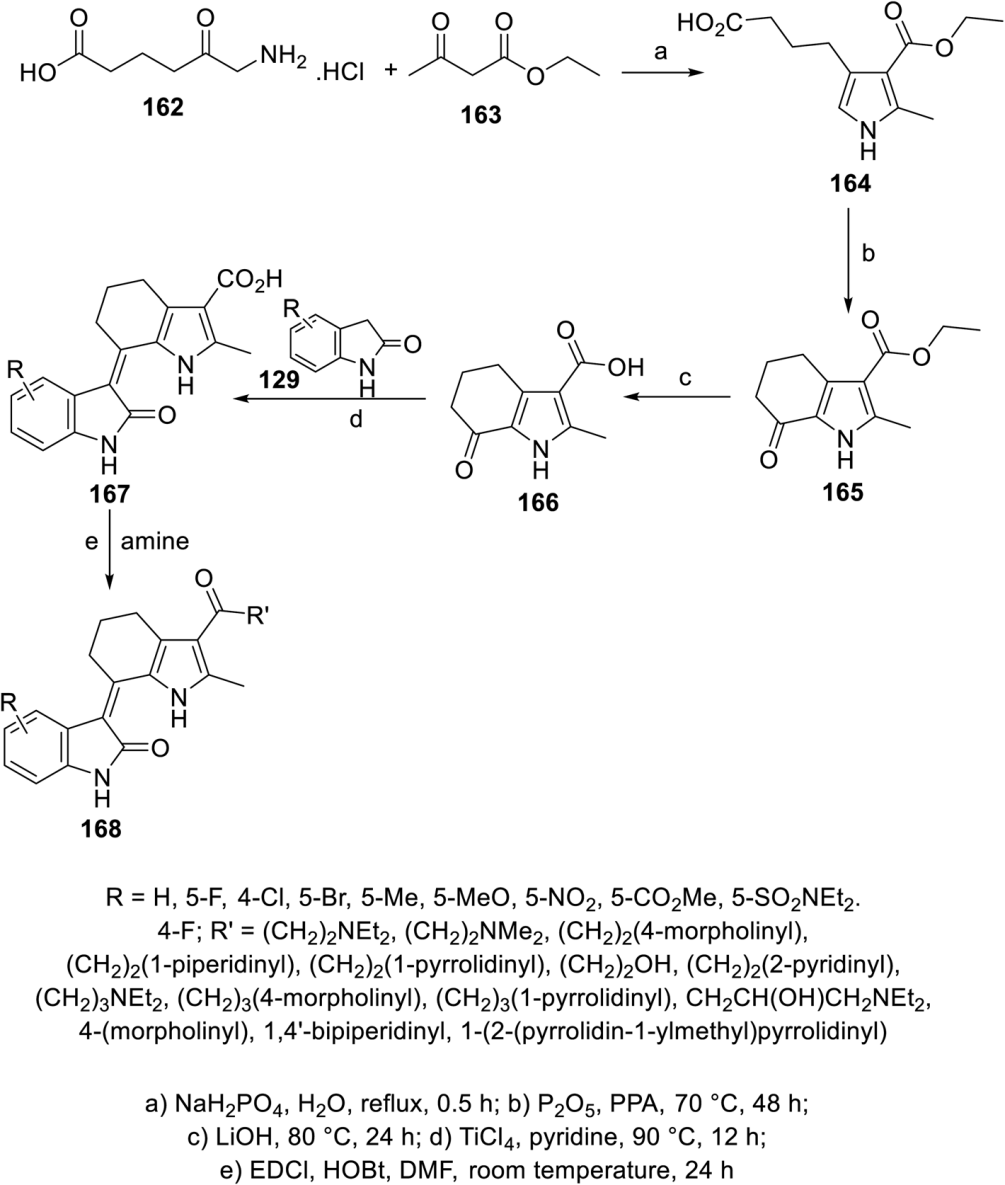
Synthesis of 2-oxoindolin-3-ylidenes 168.

A series of 2-oxoindolin-3-ylidenes connected to pyrrolo[*b*]cyclohexyl heterocycle 168 was prepared *via* the condensation (pyridine containing TiCl_4_ “catalyst”) of the ketonic function of 1*H*-indole-3-carboxylate 166 with 2-indolinones 129, followed by reaction with either primary or secondary amines (in DMF containing EDCI: 1-ethyl-3-(3-dimethylaminopropyl)carbodiimide and HOBt: hydroxybenzotriazole at room temperature) (Scheme 22). Some of the synthesized agents 168 showed promising inhibitory properties against different/multi-targeted tyrosine kinases (VEGFR-2, PDGFR-β and c-kit) compared to that of sunitinib. Furthermore, some of the promising agents were tested for their antiproliferation properties (MTT assay) against BXPC-3 (pancreatic), T24 (bladder), BGC (gastric), HEPG2 (liver) and HT29 (colon) cancer cell lines. Among them, compound 168c (R = 5-Cl, R′ = NH(CH_2_)_2_NEt_2_) was one of the most promising candidates discovered with antiproliferation (IC_50_ = 1.95, 1.83, 2.03, 3.14, and 6.48 μM against BXPC-3, T24, BGC, HEPG2 and HT29, respectively) and enzymatic inhibitory properties (IC_50_ = 2.6, 12.1, and 2.7 nM against VEGFR-2, PDGFR-β and c-kit, respectively) comparable to that of sunitinib (IC_50_ = 3.63, 2.44, 4.78, 5.61 and 1.47 μM against BXPC-3, T24, BGC, HEPG2 and HT29; IC_50_ = 4.0, 10.6, and 8.9 nM against VEGFR-2, PDGFR-β and c-kit, respectively)^114^ (ESI Fig. S11†).

**Scheme 23:**
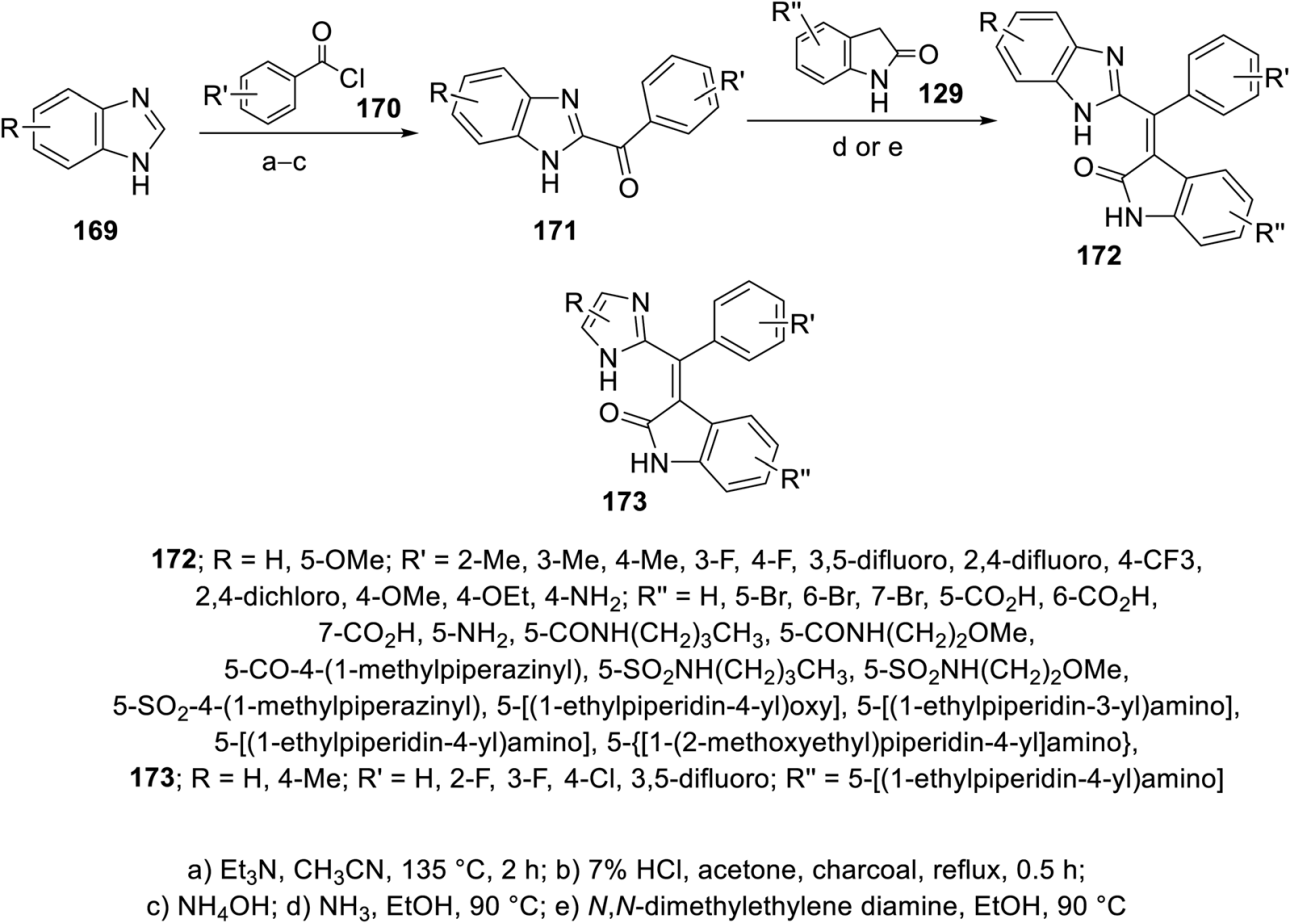
Synthesis of 2-oxoindolin-3-ylidenes 172 and 173.

The reaction of benzimidazoles 169 with benzoyl chlorides 170 at 135 °C in the presence of TEA led to the formation of *N*- and *C*-diacylated benzimidazoles. The *N*-acyl function was removed upon refluxing with 7% aqueous HCl. Condensation of the formed benzimidazolyl ketones 171 with 2-indolinones 129 in a sealed tube (90 °C) in EtOH/NH_3_ gave the targeted 2-oxoindolin-3-ylidenes 172. Similarly imidazolyl connected to 2-oxoindolin-3-ylidenes 173 were obtained (Scheme 23).^115^ Some of the synthesized 2-oxoindolin-3-ylidenes 172/173 revealed promising enzymatic inhibitory properties against VEGFR-1 (Flt-1), VEGFR-2 (KDR), FGFR-1 and PDGFR-α compared to sunitinib and SU6668 (standard references). Compounds 172p (R = R′ = H, R′′ = 5-CO_2_H), 172t (R = H, R′ = 4-Me, R′′ = 5-CO_2_H) and 172v (R = 5-OMe, R′ = H, R′′ = 5-CO_2_H) exhibited considerable potencies against VEGFR-2 compared to the standard references used (IC_50_ = 4, 5, and 3 nM, respectively). Additionally, compounds 172af {R = 5-OMe, R′ = H, R′′ = 5-NH[4-(*N*-ethylpiperidinyl)]} and 173c {R = H, R′ = 4-Cl, R′′ = 5-NH[4-(*N*-ethylpiperidinyl)]} (IC_50_ = 10, 4 nM, respectively) showed promising enzymatic inhibitory, ADME properties and cellular potencies^115^ (ESI Fig. S12†).

## 5. Indole heterocycle conjugates

Bio-conjugation is one of the most powerful and attractive rational drug design strategies used for the development of new drug candidates by connecting two or more therapeutic pharmacophoric functions/fragments/agents.^116,117^ Many conjugates have been reported to possess diverse potential bio-properties, among which vasodilation is useful for smooth muscle relaxation,^118,119^ anti-inflammatory, analgesic,^120–123^ antibacterial,^124–126^ antimycobacterial,^127^ antimalarial,^128^ antiviral including anti-SARS-CoV-2,^129–133^ HCV “hepatitis C viruses” and chikungunyan,^134^ anti-parasitic^135^ and antitumor.^136,137^

### 5.1. Indole triazole conjugates

**Scheme 24:**
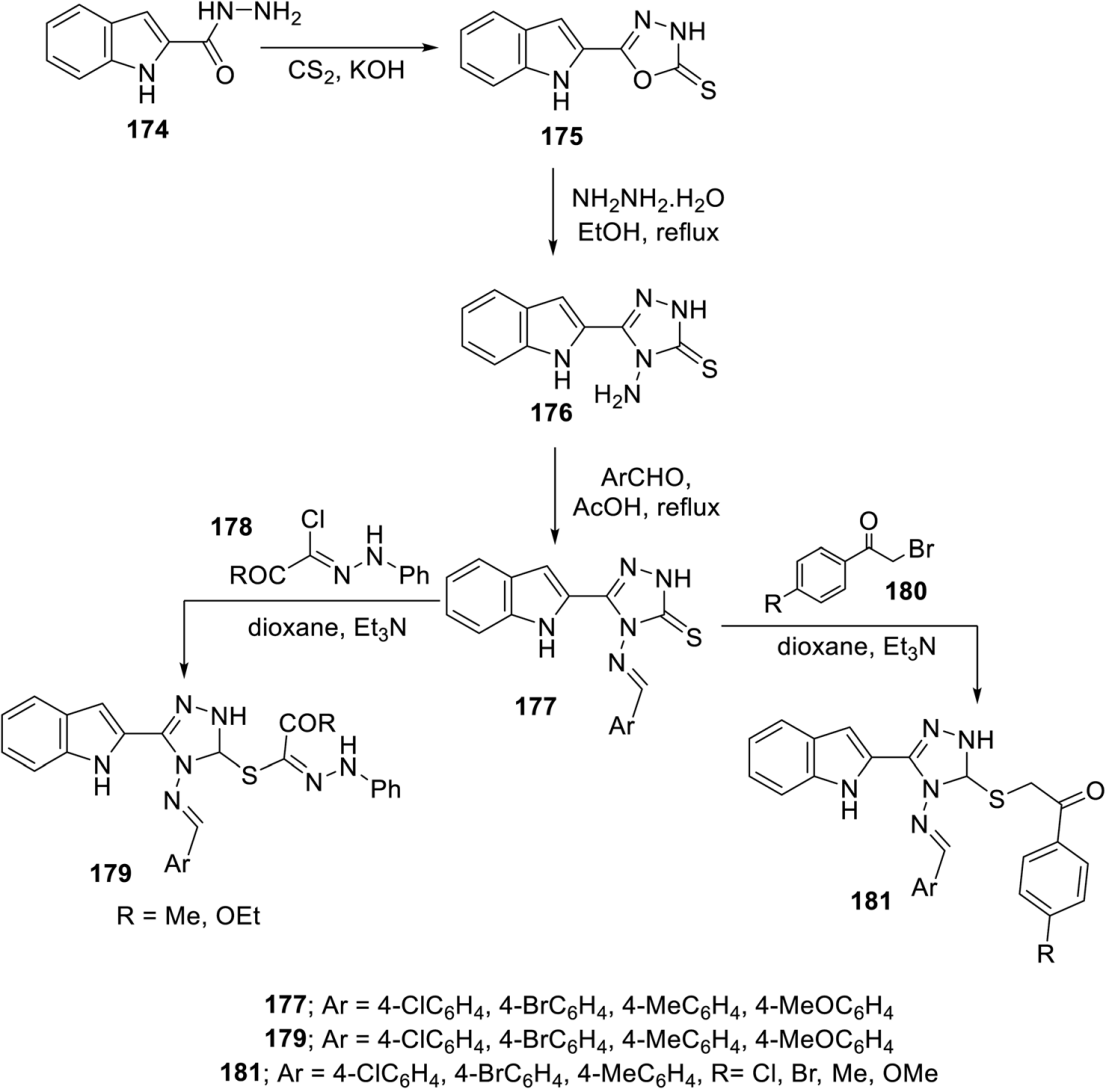
Synthesis of indole triazole conjugates 179 and 181.

The condensation reaction (in refluxing acetic acid) of the appropriate aromatic aldehyde with indole triazole conjugate bearing an amino function 176 (obtained through reaction of 2-indolecarbohydrazide 174 with CS_2_/KOH followed by hydrazonolysis (NH_2_NH_2_·H_2_O in refluxing EtOH)) afforded the corresponding Schiff base 177. The reaction of 177 with hydrazonoyl chlorides 178 or phenacyl bromides 180 (in dioxane, Et_3_N) afforded the corresponding conjugates 179 and 181 ^138^ (Scheme 24). Some of the synthesized agents were observed to exhibit considerable VEGFR-2 inhibitory and antiproliferation properties (MTT assay, against human renal cancer cell lines CAKI-1 and A498). The most promising agents discovered were 179c (Ar = 4-H_3_CC_6_H_4_; IC_50_ = 0.075 ± 0.002 μM against VEGFR-2; IC_50_ = 3.23 ± 0.15 and 2.05 ± 0.09 μM against CAKI-1 and A498 cell lines, respectively) and 181g (Ar = 4-MeOC_6_H_4_, R = Cl; IC_50_ = 0.071 ± 0.002 μM against VEGFR-2; IC_50_ = 0.89 ± 0.04 and 2.2 ± 0.1 μM against CAKI-1 and A498 cell lines, respectively) compared to sunitinib (IC_50_ = 0.075 ± 0.002 μM against VEGFR-2; IC_50_ = 4.93 ± 0.16, 1.25 ± 0.04 μM against CAKI-1 and A498 cell lines, respectively) with a safety profile upon testing against the RPTEC/TERT1 non-cancer cell line^138^ (ESI Fig. S13†).

**Scheme 25:**
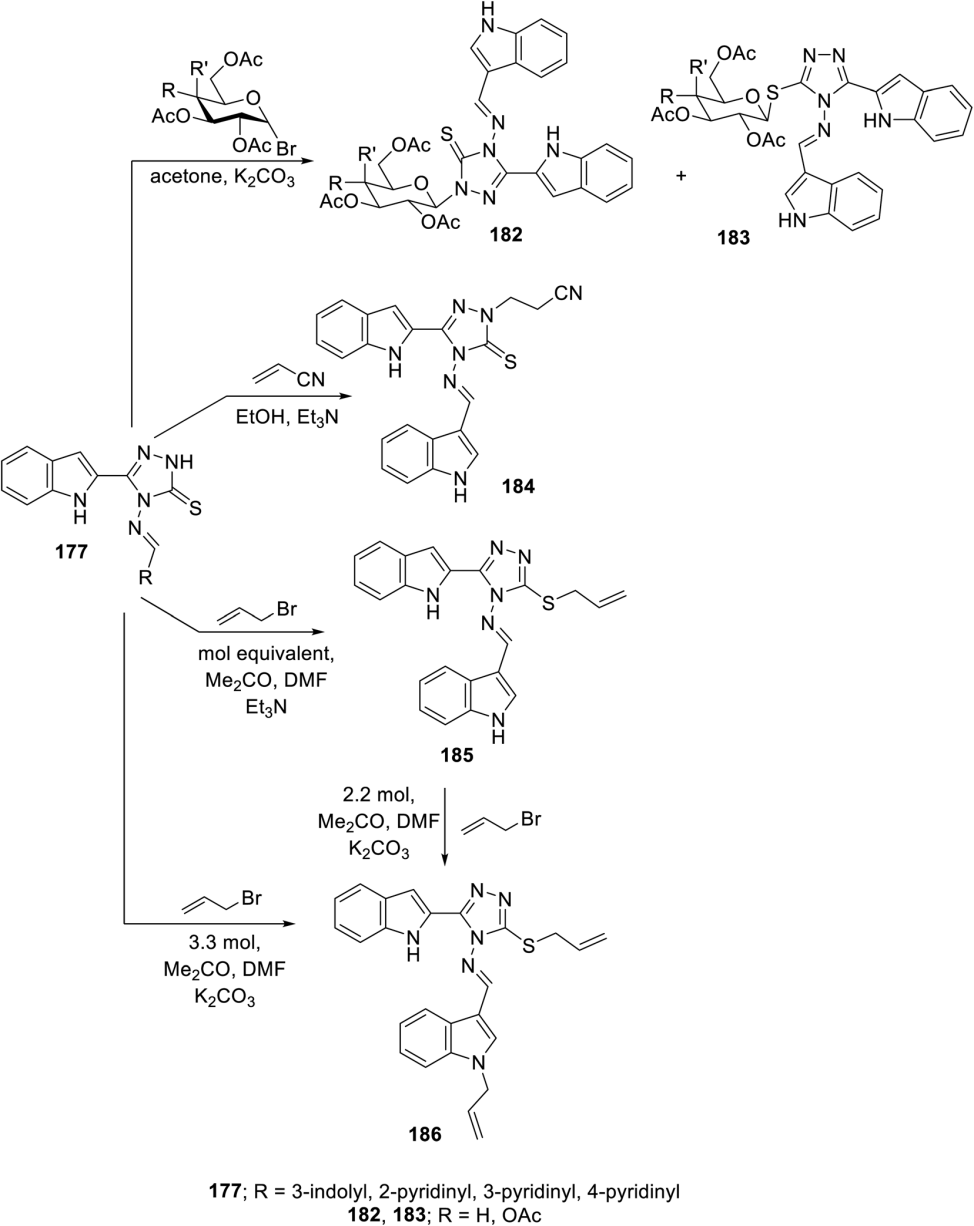
Synthesis of indole triazole conjugates 182–186.

Glycosylation of Schiff base 177 with either acetobromoglucose or acetobromogalactose (K_2_CO_3_, acetone at room temperature) gave a mixture of *N*-glycosylated 182 and *S*-analog 183. However, the reaction of 177 with acrylonitrile (Michael acceptor) in refluxing ethanol containing Et_3_N afforded the corresponding *N*-substituted Michael adduct 184. Meanwhile, the reaction of 177 with allyl bromide (equimolar values) in acetone/DMF (1 : 1 v/v) containing Et_3_N at room temperature as a basic catalyst gave the *S*-allylated analog 185. Meanwhile, utilizing K_2_CO_3_ in the same reaction (using 3.3 molar value equivalent of allyl bromide relative to 177), the allylation of both the indolyl nitrogen and *S*-function of triazolyl heterocycle 186 was achieved. The same product was obtained upon reacting *S*-allylated analog 185 under the same conditions (2.2 molar equivalent value) (Scheme 25). The antiproliferation properties of the synthesized conjugates 182–186 were studied against the MCF7 (breast) and HepG2 (liver) cancer cell lines (MTT assay) utilizing 5-flurouracil and sorafenib as reference standards. Also, the VEGFR-2 inhibitory properties were studied for the most effective antiproliferative agent observed, supporting that compound 185 is a promising conjugate (IC_50_ = 1.18 ± 0.15 and 7.09 ± 0.67 μM against MCF7 and HepG2 cell lines; IC_50_ = 19.8 ± 1.58 nM against VEGFR-2, respectively) compared to sorafenib (IC_50_ = 2.13 ± 0.24 and 3.24 ± 0.23 μM against MCF7 and HepG2 cell lines; IC_50_ = 30.0 nM against VEGFR-2, respectively). The safe profile of compound 185 was supported through antiproliferation studies against the MDA-MB-231 non-cancer cell line (triple-negative breast cancer) and normal breast cells (MCF-10A) (IC_50_ = 10.49, 24.76 μM, against MDA-MB-231 and MCF-10A, respectively)^139^ (ESI Fig. S14†).

### 5.2. Indole benzimidazole conjugates

**Scheme 26:**
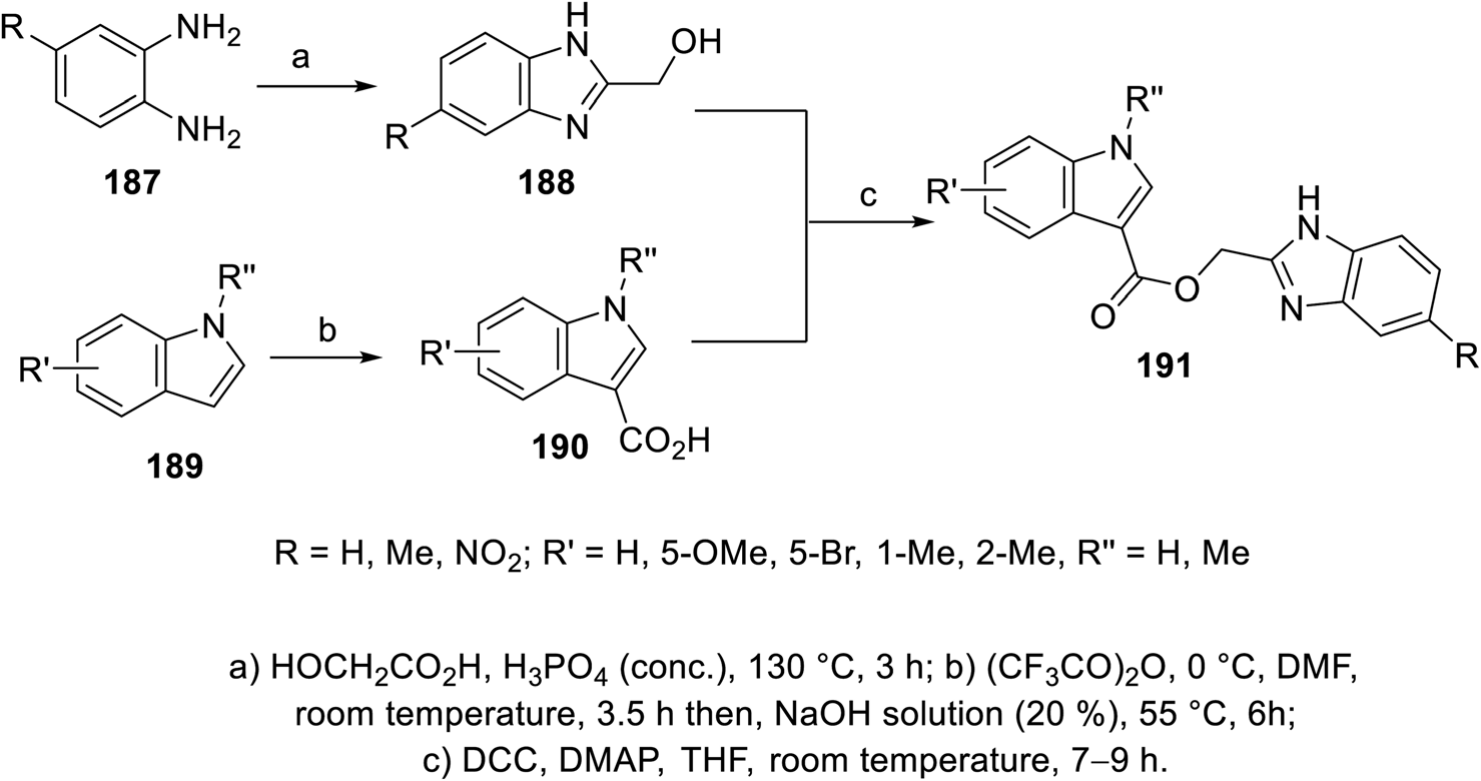
Synthesis of indole benzimidazole conjugates 191.

**Scheme 27:**
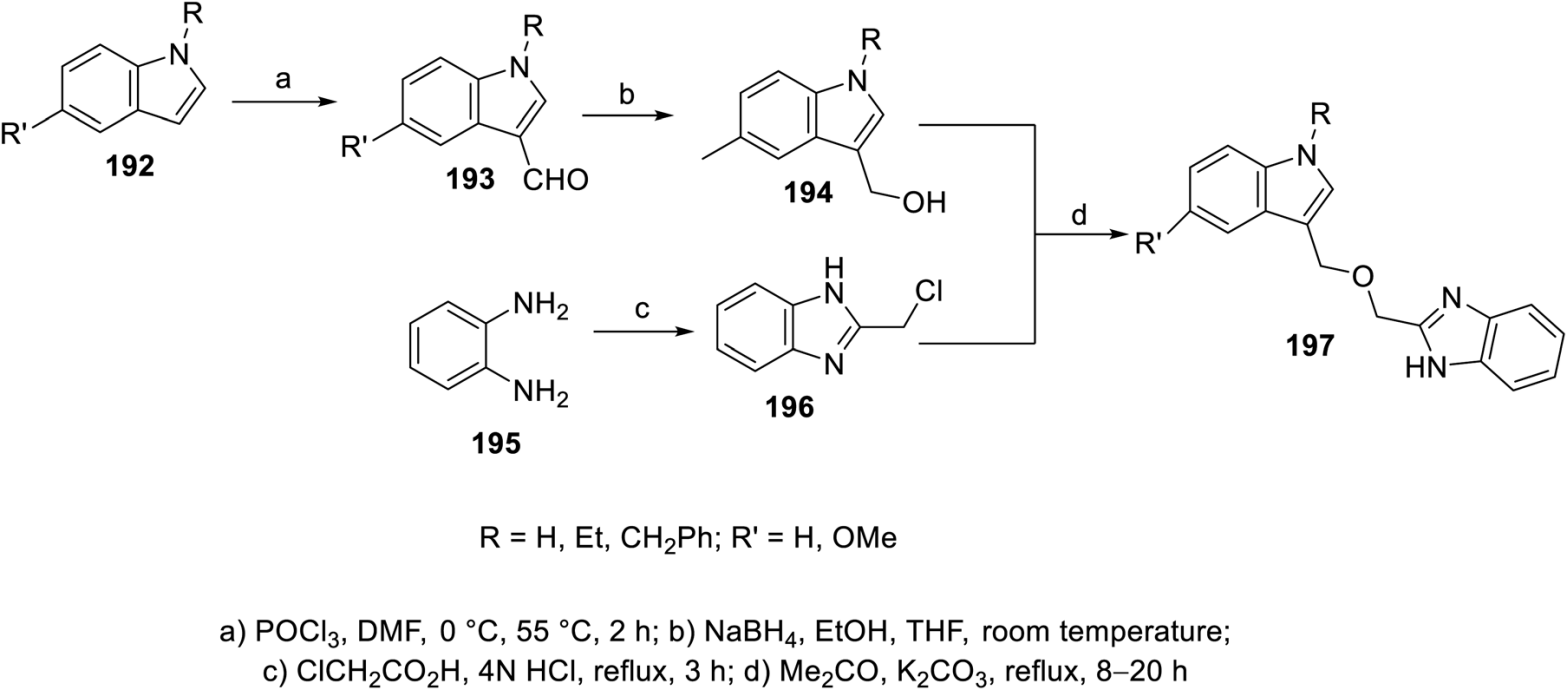
Synthesis of indole benzimidazole conjugates 197.

The esterification reaction of 3-indolecarboxylic acid 190 (obtained through the reaction of indole analogs 189 with (CF_3_CO)_2_O in DMF followed by treatment with 20% NaOH solution at 55 °C) with 2-hydroxymethyl benzimidazole 188 (synthesized through the reaction of the appropriate *o*-phenylenediamine 187 with hydroxyacetic acid in conc. H_3_PO_4_ at 130 °C) in THF containing DMAP (dimethylaminopyridine) and (DCC) (dicyclohexylcarbodiimide) at room temperature afforded indole benzimidazole conjugates 191 (Scheme 26). Additionally, indole benzimidazole conjugates 197 were obtained *via* the reaction of 2-chloromethyl-1*H*-benzimidazoles 196 (prepared by refluxing the appropriate *o*-phenylenediamine 195 with chloroacetic acid in 4 N HCl) with the appropriate (1*H*-indol-3-yl)methanol 194 (obtained through the formylation of the appropriate indolyl analogs “POCl_3_, DMF, 55 °C” followed by NaBH_4_ reduction) in refluxing acetone containing anhydrous K_2_CO_3_ (Scheme 27). Some of the synthesized conjugates exhibited considerable VEGFR-2 inhibitory properties (ESI Fig. S15†). Among them, the most promising was 191j (R = R′ = H, R′′ = Me; % inhibition of VEGFR-2 at 10 μM = 6.0) relative to sunitinib/SU11248 (% inhibition of VEGFR-2 at 10 μM = 98.1).^140^

### 5.3. Indole benzothiazole conjugates

**Scheme 28:**
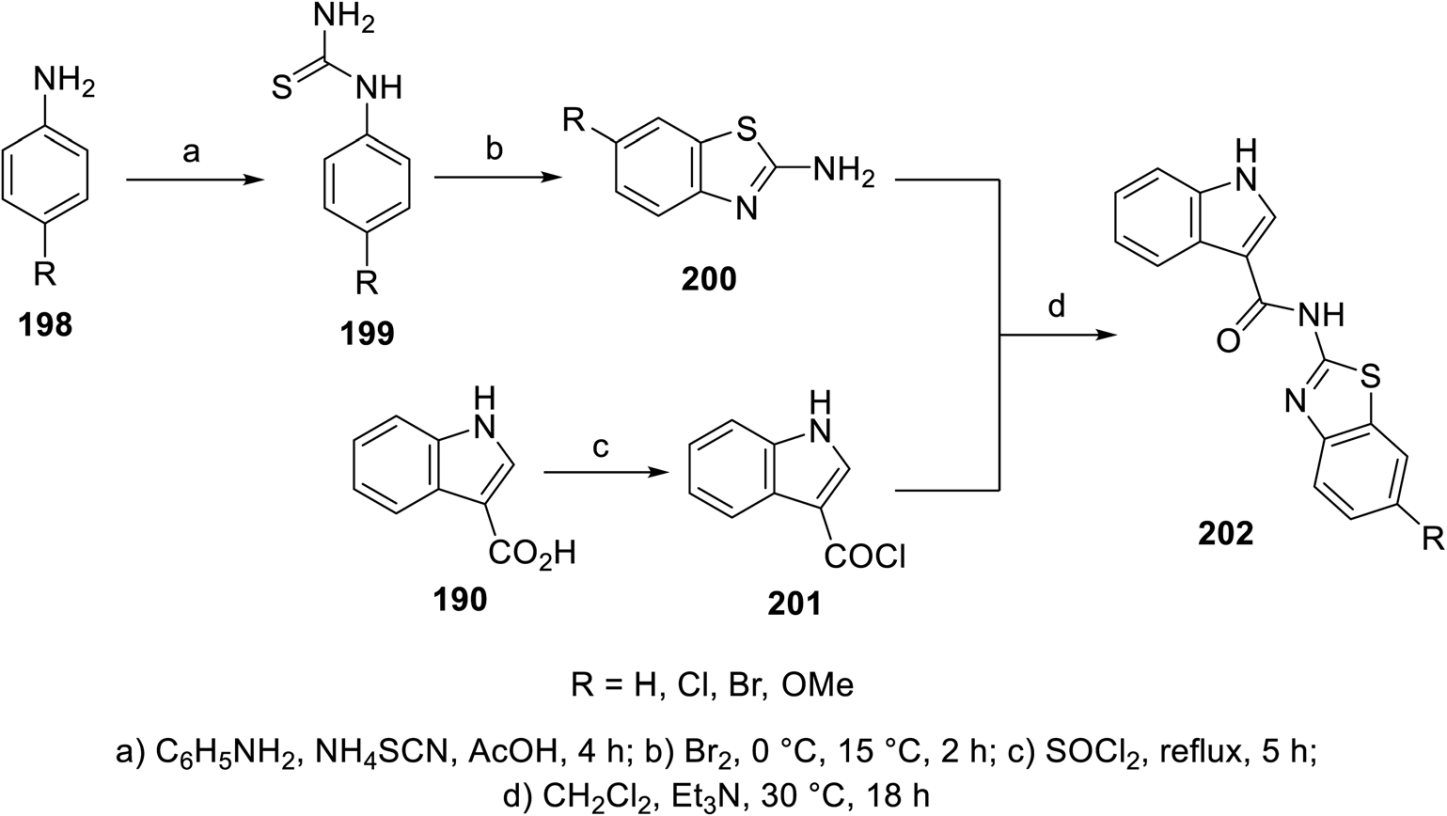
Synthesis of indole benzothiazole conjugates 202.

**Fig. 13:**
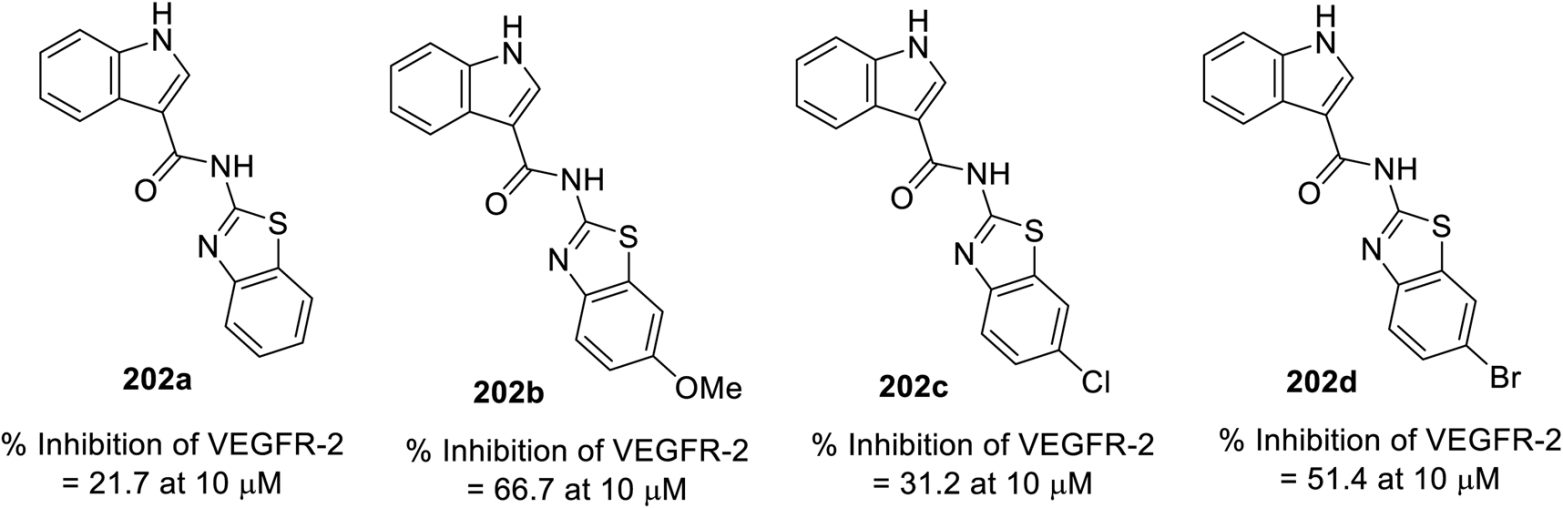
% Inhibitory properties of VEGFR-2 by indole benzothiazole conjugates 202 at 10 μM.

A variety of indole benzothiazole conjugates 202 was obtained through the reaction of 3-indole carbonyl chloride 201 (obtained by refluxing 3-indolecarboxylic acid 190 with thionyl chloride) with 2-aminobenzothiazoles 200 (in CH_2_Cl_2_ containing Et_3_N at room temperature) (Scheme 28). The synthesized conjugates exhibited considerable inhibitory properties (Fig. 13) compared to that of sunitinib (% inhibition of VEGFR-2 at 10 μM = 98.1).^140^

### 5.4. Indole–pyrimidine conjugates

**Fig. 14:**
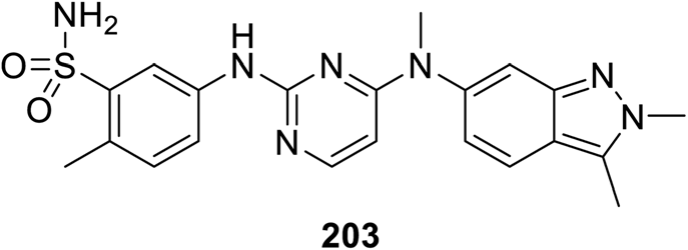
Pazopanib VEGFR-2 inhibitor FDA approved for treatment of advanced renal cell cancer and soft tissue sarcoma.

**Scheme 29:**
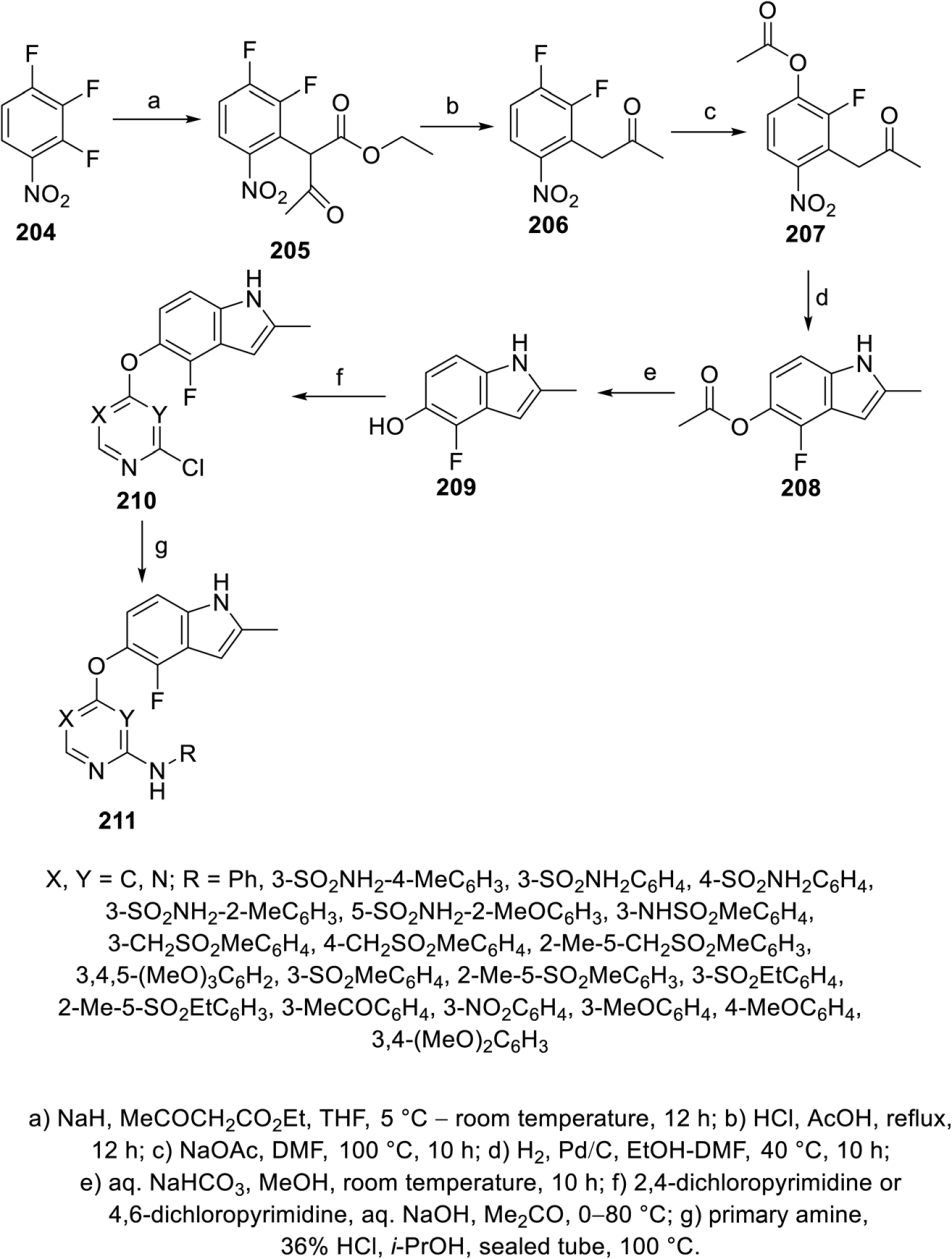
Synthesis of indole-pyrimidine conjugates 211.

Pazopanib 203 (Fig. 14) is an FDA approved VEGFR-2 inhibitor for the treatment of advanced renal cell cancer (2009) and soft tissue sarcoma.^141,142^ In this molecule, the pyrimidinyl pharmacophoric heterocycle is attached to an indazole heterocycle. This inspired the design, construction and VEGFR-2 inhibitory properties investigation of indole pyrimidine conjugates linked through an ether linkage.^143^ A set of indole pyrimidine conjugates 211 was synthesized through the coupling of 5-hydroxyindole derivative 209 with 2,4-dicholoropyrimidine or 4,6-dicholoropyrimidine in Me_2_CO containing aq. NaOH, followed by reaction with various primary amines (HCl “36%”, *i*-PrOH, sealed tube, 100 °C) (Scheme 29). Some of the synthesized conjugates 211 were observed to exhibit considerable VEGFR-2 inhibitory properties. Among them, the most promising was 211k [R = 3-(MeSO_2_C_6_H_4_)CH_2_] compared to sunitinib (IC_50_ = 0.0038 ± 0.0033, 0.0022 ± 0.0005 μM, respectively) (ESI Fig. S16†). Additionally, compound 211k exhibited inhibitory properties against VEGFR-1, VEGFR-3, PDGFR-α and PDGFR-β (IC_50_ = 40.4, 515.8, 24.1 and 33.6 nM, respectively) and can be nominated as multi-targeted tyrosine kinase inhibitor.^143^

**Scheme 30:**
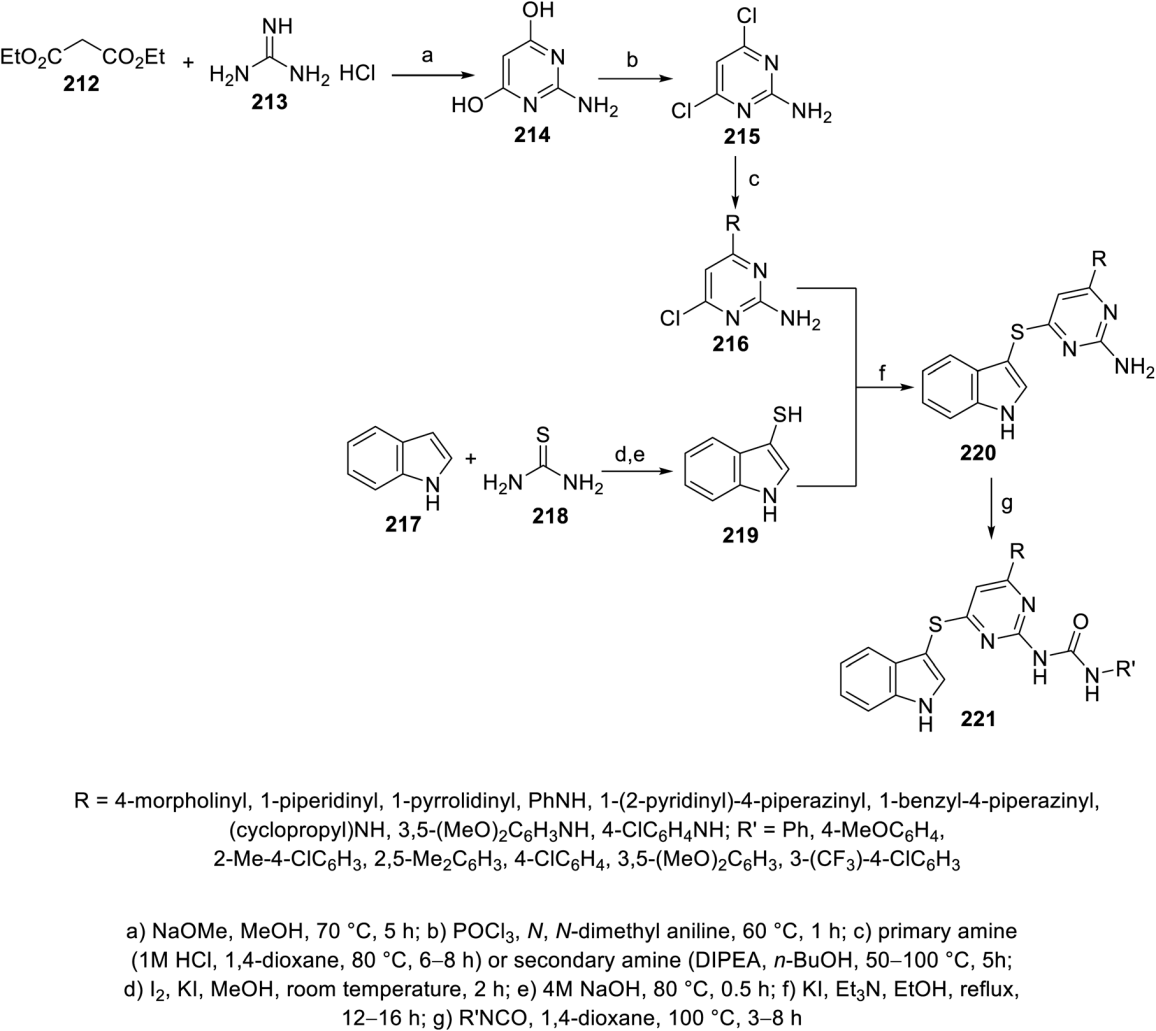
Synthesis of indole-pyrimidine conjugates 221.

Indole pyrimidine conjugates 221 connected through a thioether linkage were also reported. The reaction of indole-3-thiol 219 with 6-chloro-2,4-diaminopyrimidines 216 (obtained from condensation of diethylmalonate 212 and guanidine hydrochloride 213 under basic condition, followed by chlorination with POCl_3_, and then reaction with either primary or secondary amine) in refluxing ethanol containing Et_3_N and KI gave 220, which was reacted with isocyanate in refluxing 1,4-dioxane, finally giving 221 ^144^ (Scheme 30). Some of the synthesized agents exhibited promising antiproliferation properties (MTT assay) against A549 (lung), PC-3 (prostate), MDAMB-231 (breast) and HepG2 (liver) human cancer cell lines with promising VEFGR-2 properties compared to that of sorafenib. The most promising was 221k (R = 1-pyrrolidinyl, R′ = 4-ClC_6_H_4_; IC_50_ = 6.41 ± 0.81, 10.42 ± 0.78, 5.85 ± 0.71, and 7.87 ± 1.18 μM against A549, PC-3, MDAMB-231 and HepG2, respectively with % inhibition = 0.33% ± 0.04% of VEGFR-2 at 10 μM) compared to that of Sorafenib (IC_50_ = 7.43 ± 0.81, 9.77 ± 1.12, 11.84 ± 1.25, 5.78 ± 0.41 μM against A549, PC-3, MDAMB-231 and HepG2, respectively with % inhibition = 1.21% ± 0.02% of VEGFR-2 at 10 μM)^144^ (ESI Fig. S17†).

### 5.5. Indole chromene conjugates

**Scheme 31:**
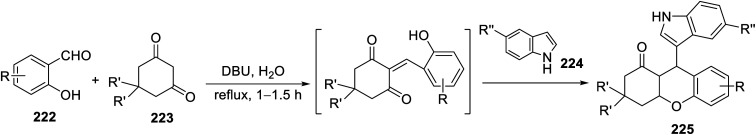
Synthesis of indole chromene conjugates 225.

**Fig. 15:**
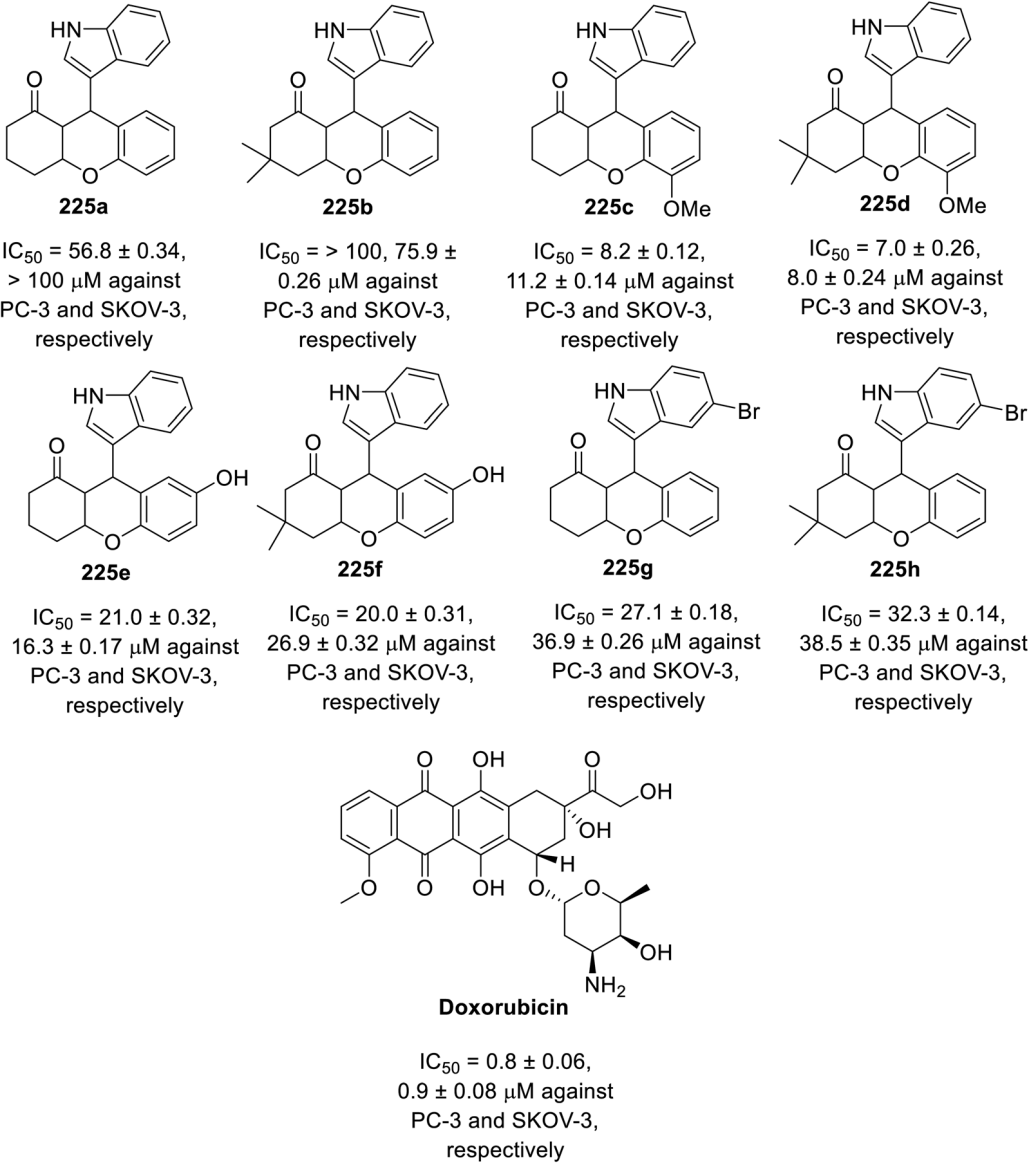
Antiproliferation properties of indole chromene conjugates 225 and doxorubicin (standard reference).

A multi-component eco-friendly synthetic procedure was employed for the preparation of indole chromene conjugates 225*via* the one-pot reaction of salicaldehydes 222, with the appropriate 1,3-cyclohaxanones 223 and indole derivative 224 in refluxing water containing DBU (1,8-diazabicyclo[5.4.0]undec-7-ene). The reaction was assumed to take place *via* the base-catalyzed Michael addition of indole to the Knoevenagel adduct formed from the condensation of the salicaldehyde with 1,3-cyclohexanone assisted by the basic catalysis of DBU^145^ (Scheme 31). The synthesized conjugates 225 exhibited mild antitumor properties (MTT assay) against the PC-3 (prostate) and SKOV-3 (ovarian) cancer cell lines compared to that of doxorubicin (Fig. 15). Their VEGFR-2 inhibitory properties were reported based on the molecular modeling technique (PDB ID: 4ASD, AutoDock Tools 4.2).^145^ However, theoretical (molecular modeling) studies are insufficient supporting evidence for assigning the mode of action, and thus experimental data is required.

## 6. Spiroindoles

Spiroindoles occupy a unique place in heterocyclic chemistry due to the versatile bio-properties of their natural and synthetic analogs^146^ (exemplified by antibacterial, antifungal,^147–151^ antimycobacterial,^152–154^ antiviral,^19,155^ anticancer,^156–160^ antimalarial,^161,162^ anti-inflammatory^163^ and antihyperglycemic^164,165^). [3 + 2]-Dipolar cycloaddition of azomethine ylides derived from isatin derivatives with α-amino acids to the exocyclic olefinic linkage derived from alicycles or heterocycles is an accessible synthetic approach successfully used for the construction of various spiroindoles with regio- and stereoselectivity.^146,166–168^ Many antitumor active agents against various human tumor cell lines were optimized *via* the above-mentioned synthetic approach; however, the VEGFR mode of action was only assigned for a few analogs.^169,170^

**Scheme 32:**
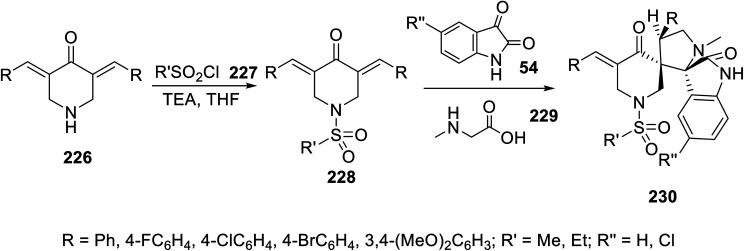
Synthesis of spiroindoles 230.

Spiroindoles 230 were regioselectively synthesized through the one-pot three-component reaction of 3,5-diylidene-4-piperidones 228 and azomethine, which were formed by the condensation of isatins 54 and sarcosine 229 in refluxing ethanol (Scheme 32). The stereochemical structure of 230 was established by single crystal X-ray studies. Promising antiproliferation properties were exhibited by some of the synthesized spiroindoles 230 against the MCF7 (breast), HCT116 (colon), A431 (skin squamous) and PaCa2 (pancreatic) cancer cell lines (MTT assay) compared to the standard references (sunitinib and 5-fluorouracil). Safe behaviors against the non-cancer RPE1 cell line were revealed by the synthesized agents. Considerable multi-targeted inhibitory properties (western blotting technique) were exhibited by the synthesized agents against VEGFR-2 and EGFR. The SAR (structure–activity relationship) study indicated that the chloro-substituted indolyl heterocycle can optimize efficient agents against the tested cancer cell lines. Compound 231n (R = 4-BrC_6_H_4_, R′ = Me, R′′ = H) was the most promising agent observed (IC_50_ = 3.597 ± 0.19, 3.236 ± 0.27, 2.434 ± 0.18, 12.500 ± 0.67, and 14.894 ± 1.61 μM against the MCF7, HCT116, A431, PaCa-2 and RPE1 cell lines, respectively; % inhibition = 61.3% and 65.6% against VEGFR-2 and EGFR at the IC_50_ value observed against MCF7, respectively) compared to sunitinib (IC_50_ = 3.97 ± 0.32, 9.67 ± 0.22, and 16.91 ± 0.95 μM against MCF7, HCT116, and PaCa-2 cell lines, respectively; % inhibition = 74.7% and 81.4% against VEGFR-2 and EGFR at IC_50_ value observed against MCF7, respectively)^19^ (ESI Fig. S18†).

## 7. Conclusion

Tyrosine kinases are capable of many diverse cellular functions including growth, proliferation, differentiation and death. VEGFR is one of the targeted therapeutic approaches that is preferable to the classical non-selective therapies to minimize the associated side effects or drawbacks. VEGF is an important category of tyrosine kinases, which can stimulate angiogenesis. VEGFR-2 is the most well-known factor in the angiogenesis of different solid tumors (colon, breast, ovary, lung, skin, renal, head, neck, lymphoma, *etc.*). Indolyl therapeutics have been approved against some serious types of cancer. Additionally, research efforts identified natural and synthesized antitumor indole-containing compounds with promising anti-VEGFR properties. Computational/theoretical studies can assist in designing and identifying novel hits/leads of anti-VEGFR agents; however, without experimental supporting their enzymatic properties, the predictions cannot be considered for further investigations or utilization.

## Abbreviations

ADMET: Absorption, distribution, metabolism, excretion and toxicity
AMD: Age-related macular degeneration
ATP: Adenosine triphosphate
BOP: Benzotriazol-1-yloxytris(dimethylamino)phosphonium hexafluorophosphate
CA: Carbonic anhydrase
CAM: Chick chorioallantoic membrane
CDK-1: Cyclin-dependent kinase 1
c-kit: Stem cell factor receptor
COX: Cyclooxygenase
COX-2: Cyclooxygenase-2
CSF-1R: Colony stimulating factor-1 receptor
DBU: 1,8-Diazabicyclo[5.4.0]undec-7-ene
DCM: Dichloromethane
DFT: Density function theory
DIPEA: *N*,*N*-Diisopropylethylamine
DMTMM: 4-(4,6-Dimethoxytriazine)chlorinated 4-methylmorpholine
EDCI: 1-Ethyl-3-(3-dimethylaminopropyl)carbodiimide
EGF: Epidermal growth factor
EGFR: Epidermal growth factor receptor
FDA: Food and drug administration
FGF: Fibroblast growth factor
FGFR: Fibroblast growth-factor receptor
HATU: Hexafluorophosphate azabenzotriazole tetramethyl uronium
HCV: Hepatitis C viruses
HER-2: Human epidermal growth factor receptor 2
HIF-1-α: Hypoxia-inducible factor-1-alph
HIV: Human immunodeficiency virus
HOBt: Hydroxybenzotriazole
HUVEC: Human umbilical vein endothelial cell
IBCF: Iso-butyl chloroformate
MD: Molecular dynamic
MTT: 3-(4,5-Dimethylthiazol-2-yl)-2,5-diphenyltetrazolium bromide
NET: Neuroendocrine tumor
NSCLC: Non-small cell lung cancer
NT: Not tested
PDGFR: Platelet-derived growth-factor receptor
SAR: Structure–activity relationship
SD: Standard division
SEM: Standard error mean
SRB: Sulforhodamine B
TEA: Triethylamine
THF: Tetrahydrofuran
TRAMP: Transgenic adenocarcinoma of the mouse prostate
VEGF: Vascular endothelial growth factor
VEGFR: Vascular endothelial growth factor receptor

## Author contributions

Conceptualization, A. S. G.; methodology, A. S. G. and D. R. A.; data analyses, A. S. G.; writing – original draft preparation, A. S. G. and D. R. A.; review and editing, A. S. G., D. R. A., M. S. B. and A. R. H.; supervision, A. S. G. and M. A. Y. All authors have read and agreed to the published version of the manuscript.

## Conflicts of interest

There is no conflict to declare.

## Supplementary Material

RA-014-D3RA08962B-s001
