# Supplementary material for: Antineoplastic indole-containing compounds with potential VEGFR inhibitory properties
Source: RSC Adv. 2024 Feb 14;14(9):5690–728. doi: 10.1039/d3ra08962b (PMC10866129; doi:10.1039/d3ra08962b)
Supplement: RA-014-D3RA08962B-s001 [file RA-014-D3RA08962B-s001.pdf]

Electronic Supplementary Material (ESI) for RSC Advances.
This journal is © The Royal Society of Chemistry 2024

Antineoplastic indole-containing compounds with potential VEGFR inhibitory properties

Dalia R. Aboshouk,[a] M. Adel Youssef,[b] Mohamed S. Bekheit,[a] Ahmed R. Hamed[c] and Adel S. Girgis*[a]

[a]*Department of Pesticide Chemistry, National Research Centre, Dokki, Giza 12622, Egypt*

[b]*Department of Chemistry, Faculty of Science, Helwan University, Helwan, Egypt*

[c]*Chemistry of Medicinal Plants Department, National Research Centre, Dokki, Giza 12622, Egypt*

* Corresponding author: girgisas10@yahoo.com, as.girgis@nrc.sci.eg

Supplementary material

Figure captions

Fig. S1. Antiproliferation and anti-VEGFR-2 properties of the synthesized indole-2-carboxamides **49a–h** and reference standards (Erlotinib and Sorafenib).

Fig. S2. Activity of 5-indolecarboxamides **53a–l** against VEGFR-2, CDK-1/cyclin B and HER-2 at 10 mM, respectively.

Fig. S3. Antiproliferation and anti-VEGFR-2 properties of the synthesized indolyl Schiff bases **55**, **57–59**, **61**, **63**, **65**, **67** and **68**.

Fig. S4. Antiproliferation and VEGFR-2 properties (µM ± SD) of the prepared Schiff bases **73a–x** and standard references (Doxorubicin and Sorafenib).

Fig. S5. Antiproliferation properties (µM ± SEM) and % inhibition of VEGFR-2 for the synthesized agents and standard references "Sunitinib and 5-Fluorouracil".

Fig. S6 . Antiproliferation and VEGFR-2 inhibitory properties of hydrazones **106a–k** and Sorafenib.

Fig. S7. Antiproliferation properties of indolyl hydrazones **113** and standard references (Cisplatin, Sorafenib and Sunitinib).

Fig S8. Antiproliferation and VEGFR-2 inhibitory properties of 2-oxoindolin-3-ylidenes **130** and Sunitinib.

Fig. S9. Antiproliferation and enzymatic inhibitory properties of 2-oxoindolin-3-ylidene **146** and Nintedanib.

Fig. S10. Antiproliferation and enzymatic inhibitory properties of 2-oxoindolin-3-ylidenes **151**,**153** and Nintedanib.

Fig. S11. Antiproliferation and enzymatic inhibitory properties of 2-oxoindolin-3-ylidenes **168** and Sunitinib.

Fig. S12. Enzymatic inhibitory properties of 2-oxoindolin-3-ylidenes **172**, **173** and standard references (Sunitinib and SU6668).

Fig. S13. VEGFR-2 inhibitory and antiproliferation properties of indole triazole conjugates **176**, **177**, **179**, **181** and Sunitinib.

Fig. S14. Antiproliferation and VEGFR-2 inhibitory properties of indole triazole conjugates **177**, **183-186** and standard references.

Fig. S15. % Inhibitory properties of VEGFR-2 by indole benzimidazole conjugates **191** and **197** at 10 mM.

Fig. S16. Inhibitory properties of VEGFR-2 by indole-pyrimidine conjugates **211** and Sunitinib.

Fig. S17. Antiproliferation and inhibitory properties of VEGFR-2 for indole pyrimidine conjugates **221** and Sorafenib.

Fig. S18. Antiproliferation and enzymatc inhibitory (VEGFR-2 and EGFR) properties of spiroindoles **230** and reference standards (Sunitinib and 5-Fluorouracil).

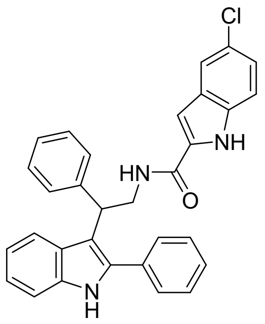

49a

IC_{50} = 26 ± 2, 28 ± 2,
26 ± 2, 25 ± 2 nM against
Panc-1, MCF7, HT-29
and A-549, respectively;
IC_{50} = 2.15 ± 0.20 nM against
VEGFR-2

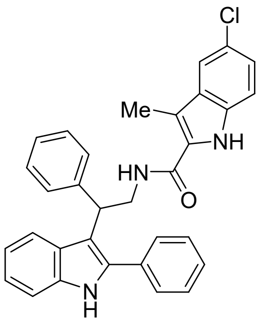

49b

IC_{50} = 58 ± 5, 61 ± 6,
59 ± 5, 58 ± 5 nM against
Panc-1, MCF7, HT-29
and A-549, respectively

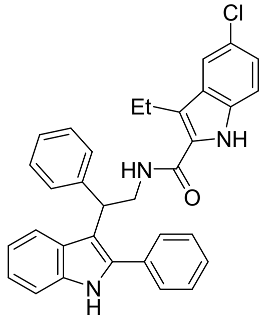

49c

IC_{50} = 56 ± 5, 57 ± 5,
55 ± 5, 54 ± 5 nM against
Panc-1, MCF7, HT-29
and A-549, respectively

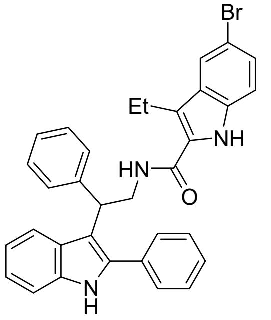

49d

IC_{50} = 66 ± 6, 68 ± 6,
66 ± 6, 64 ± 6 nM against
Panc-1, MCF7, HT-29
and A-549, respectively

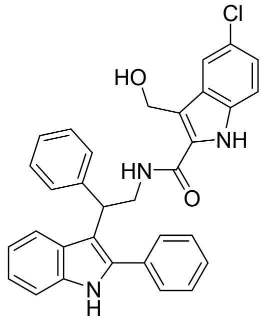

49e

IC_{50} = 44 ± 4, 46 ± 4,
45 ± 4, 42 ± 4 nM against
Panc-1, MCF7, HT-29
and A-549, respectively;
IC_{50} = 1.10 ± 0.08 nM against
VEGFR-2

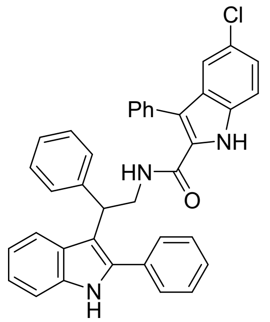

49f

IC_{50} = 48 ± 4, 49 ± 4,
48 ± 4, 46 ± 4 nM against
Panc-1, MCF7, HT-29
and A-549, respectively;
IC_{50} = 2.50 ± 0.20 nM against
VEGFR-2

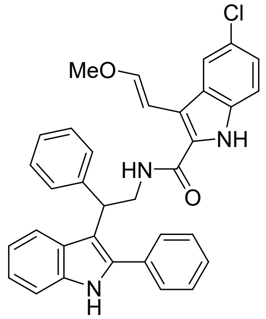

49g

IC_{50} = 30 ± 2, 33 ± 3,
30 ± 2, 30 ± 2 nM against
Panc-1, MCF7, HT-29
and A-549, respectively;
IC_{50} = 1.60 ± 0.10 nM against
VEGFR-2

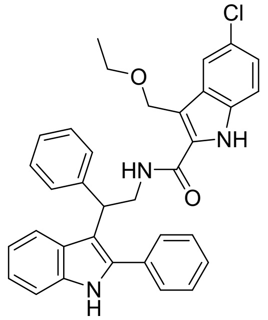

49h

IC_{50} = 36 ± 3, 38 ± 3,
38 ± 3, 34 ± 3 nM against
Panc-1, MCF7, HT-29
and A-549, respectively;
IC_{50} = 3.25 ± 0.25 nM against
VEGFR-2

Fig. S1. Antiproliferation and anti-VEGFR-2 properties of the synthesized indole-2-carboxamides **49a**–**h** and reference standards (Erlotinib and Sorafenib).

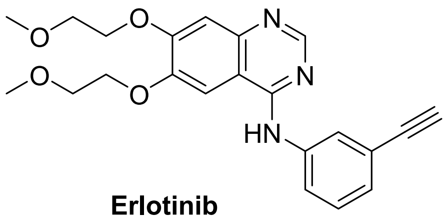

Erlotinib

IC_{50} = 30 ± 3, 40 ± 3, 30 ± 3, 30 ± 3 nM against Panc-1, MCF7, HT-29 and A-549, respectively

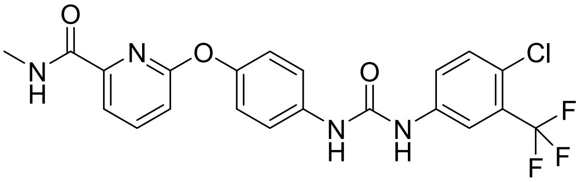

Sorafenib

IC_{50} = 0.17 ± 0.01 nM against VEGFR-2

Fig. S1 (continued). Antiproliferation and anti-VEGFR-2 properties of the synthesized indole-2-carboxamides **49a**–**h** and reference standards (Erlotinib and Sorafenib).

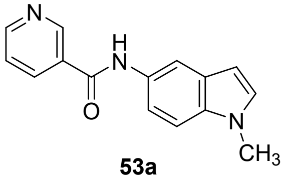

53a

% activity = 96, 91, 95
against VEGFR-2, CDK-1/cyclin B
and HER-2, respectively

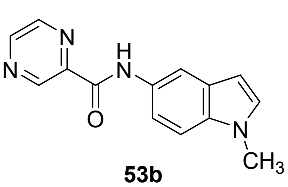

53b

% activity = 100, 100, 93
against VEGFR-2, CDK-1/cyclin B
and HER-2, respectively

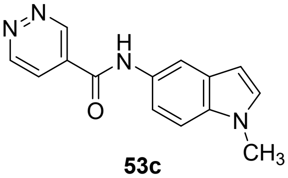

53c

% activity = 100, 91, 99
against VEGFR-2, CDK-1/cyclin B
and HER-2, respectively

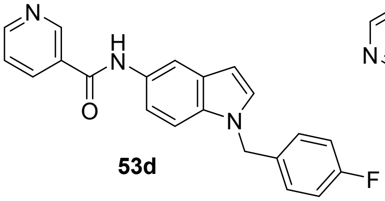

53d

% activity = 91, 85, 90
against VEGFR-2, CDK-1/cyclin B
and HER-2, respectively

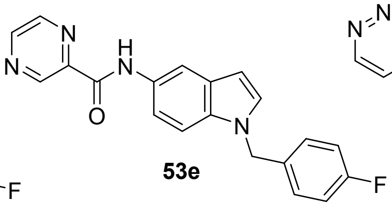

53e

% activity = 98, 88, 86
against VEGFR-2, CDK-1/cyclin B
and HER-2, respectively

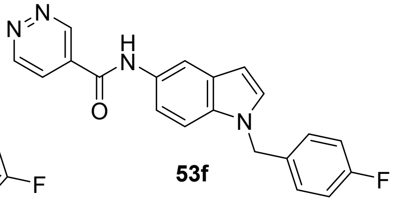

53f

% activity = 96, 91, 100
against VEGFR-2, CDK-1/cyclin B
and HER-2, respectively

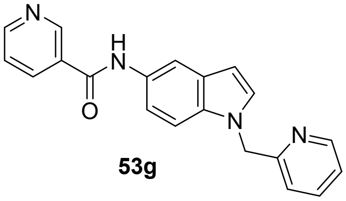

53g

% activity = 100, 92, 83
against VEGFR-2, CDK-1/cyclin B
and HER-2, respectively

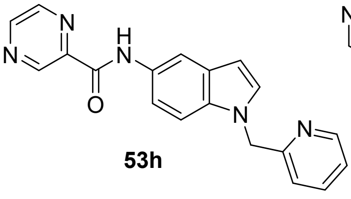

53h

% activity = 100, 95, 97
against VEGFR-2, CDK-1/cyclin B
and HER-2, respectively

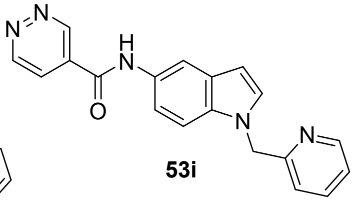

53i

% activity = 85, 89, 87
against VEGFR-2, CDK-1/cyclin B
and HER-2, respectively

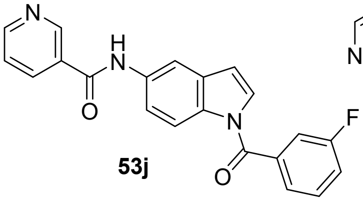

53j

% activity = 90, 91, 93
against VEGFR-2, CDK-1/cyclin B
and HER-2, respectively

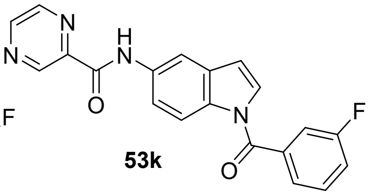

53k

% activity = 90, 92, 92
against VEGFR-2, CDK-1/cyclin B
and HER-2, respectively

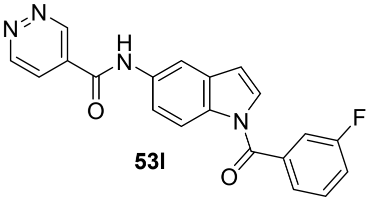

53l

% activity = 92, 51, 52
against VEGFR-2, CDK-1/cyclin B
and HER-2, respectively

Fig. S2. Activity of 5-indolecarboxamides **53a–l** against VEGFR-2, CDK-1/cyclin B and HER-2 at 10 μM, respectively.

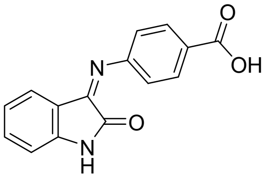

55a

IC_{50} = 62.41 ± 2.61,
51.60 ± 2.50 μM against
HepG2 and MCF7, respectively

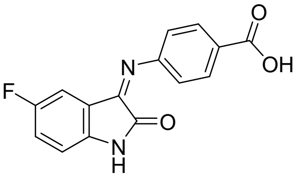

55b

IC_{50} = 51.22 ± 2.50,
43.90 ± 2.10 μM against
HepG2 and MCF7, respectively

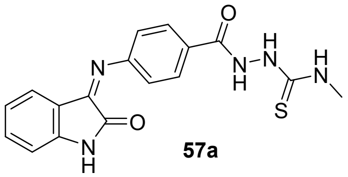

57a

IC_{50} = 5.48 ± 0.27,
6.42 ± 0.55 μM against
HepG2 and MCF7, respectively

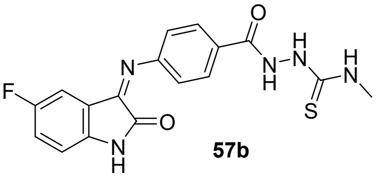

57b

IC_{50} = 2.62 ± 0.13,
0.99 ± 0.04 μM against
HepG2 and MCF7, respectively;
IC_{50} = 0.160 ± 0.008 μM
against VEGFR-2

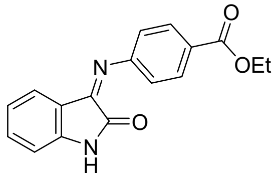

58a

IC_{50} = 10.94 ± 0.53,
14.90 ± 0.70 μM against
HepG2 and MCF7, respectively

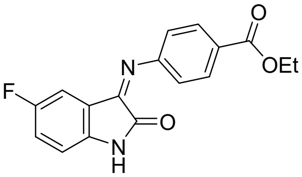

58b

IC_{50} = 2.23 ± 0.11,
3.81 ± 0.20 μM against
HepG2 and MCF7, respectively

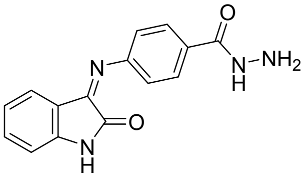

59a

IC_{50} = 24.85 ± 1.21,
27.20 ± 1.30 μM against
HepG2 and MCF7, respectively

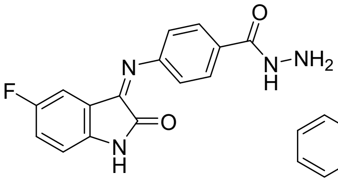

59b

IC_{50} = 53.89 ± 2.63,
40.70 ± 2.80 μM against
HepG2 and MCF7, respectively

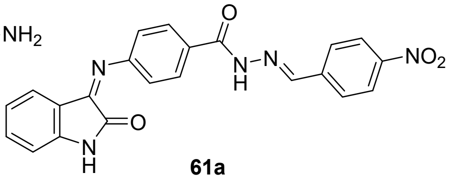

61a

IC_{50} = 8.24 ± 0.40,
40.00 ± 2.00 μM against
HepG2 and MCF7, respectively

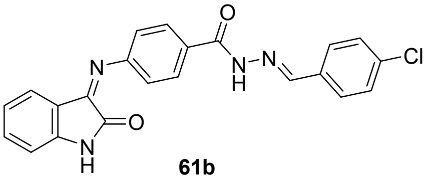

61b

IC_{50} = 33.83 ± 1.65,
23.80 ± 1.21 μM against
HepG2 and MCF7, respectively

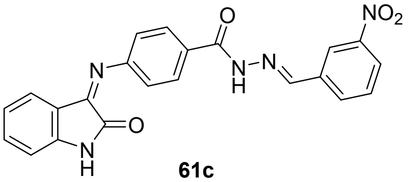

61c

IC_{50} = 20.96 ± 1.02,
14.60 ± 0.71 μM against
HepG2 and MCF7, respectively

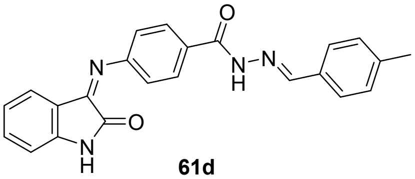

61d

IC_{50} = 21.41 ± 1.05,
13.70 ± 0.40 μM against
HepG2 and MCF7, respectively

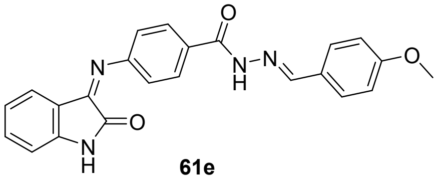

61e

IC_{50} = 8.81 ± 0.43,
4.62 ± 0.20 μM against
HepG2 and MCF7, respectively;
IC_{50} = 0.358 ± 0.019 μM
against VEGFR-2

Fig. S3. Antiproliferation and anti-VEGFR-2 properties of the synthesized indolyl Schiff bases **55**, **57**–**59**, **61**, **63**, **65**, **67** and **68**.

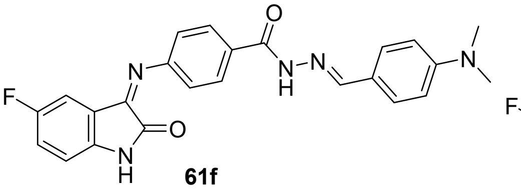

61f

IC_{50} = 19.16 ± 0.94,
11.00 ± 0.59 μM against
HepG2 and MCF7, respectively

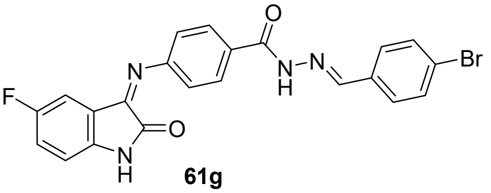

61g

IC_{50} = 2.79 ± 0.14,
0.74 ± 0.03 μM against
HepG2 and MCF7, respectively;
IC_{50} = 0.087 ± 0.004 μM
against VEGFR-2

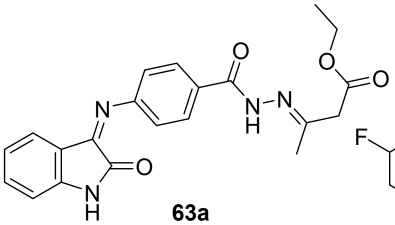

63a

IC_{50} = 10.28 ± 0.81,
7.37 ± 0.40 μM against
HepG2 and MCF7, respectively

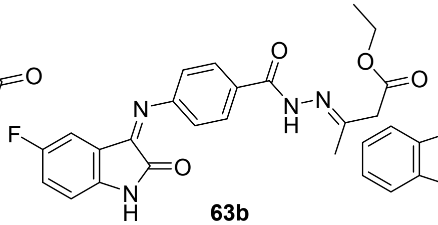

63b

IC_{50} = 13.75 ± 0.67,
32.90 ± 1.61 μM against
HepG2 and MCF7, respectively

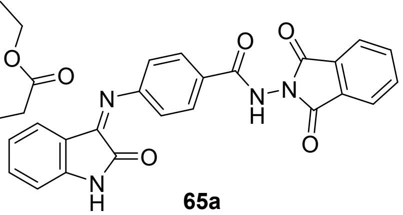

65a

IC_{50} = 130.20 ± 6.37,
78.40 ± 3.81 μM against
HepG2 and MCF7, respectively

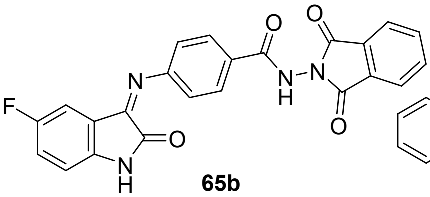

65b

IC_{50} = 23.46 ± 1.15,
33.80 ± 1.70 μM against
HepG2 and MCF7, respectively

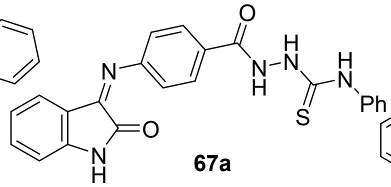

67a

IC_{50} = 1.13 ± 0.06,
1.44 ± 0.11 μM against
HepG2 and MCF7, respectively;
IC_{50} = 0.078 ± 0.003 μM
against VEGFR-2

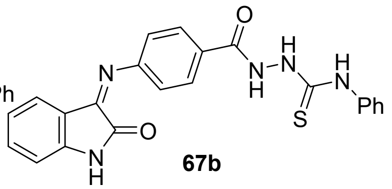

67b

IC_{50} = 9.81 ± 0.48,
25.80 ± 1.30 μM against
HepG2 and MCF7, respectively

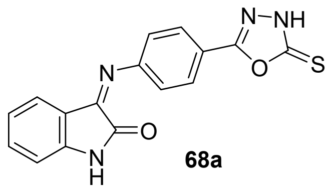

68a

IC_{50} = 2.30 ± 0.18,
2.77 ± 0.10 μM against
HepG2 and MCF7, respectively;
IC_{50} = 0.180 ± 0.009 μM
against VEGFR-2

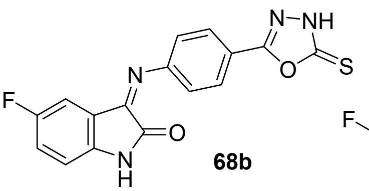

68b

IC_{50} = 12.69 ± 0.62,
19.60 ± 1.80 μM against
HepG2 and MCF7, respectively

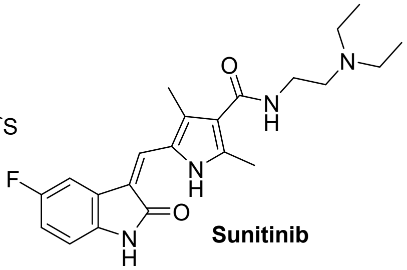

Sunitinib

IC_{50} = 2.23 ± 0.11,
4.77 ± 0.29 μM against
HepG2 and MCF7, respectively;
IC_{50} = 0.139 ± 0.007 μM
against VEGFR-2

Fig. S3 (continued). Antiproliferation and anti-VEGFR-2 properties of the synthesized indolyl Schiff bases **55**, **57-59**, **61**, **63**, **65**, **67** and **68**.

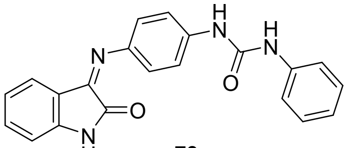

73a

IC_{50} = 3.46 ± 0.29, 5.61 ± 0.65 μM
for HepG2 cell line and VEGFR-2,
respectively

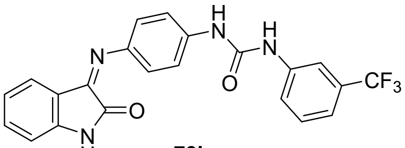

73b

IC_{50} = 3.05 ± 0.23, 4.26 ± 0.31 μM
for HepG2 cell line and VEGFR-2,
respectively

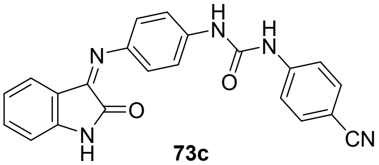

73c

IC_{50} = 7.73 ± 0.23, 5.09 ± 1.05 μM
for HepG2 cell line and VEGFR-2,
respectively

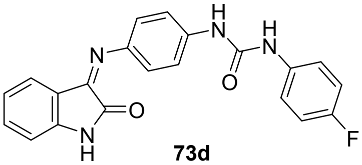

73d

IC_{50} = 10.11 ± 3.75 μM
for HepG2 cell line

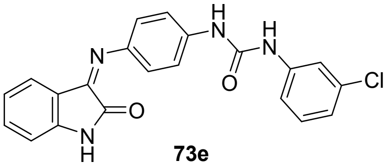

73e

IC_{50} = 3.20 ± 0.36, 2.58 ± 0.18 μM
for HepG2 cell line and VEGFR-2,
respectively

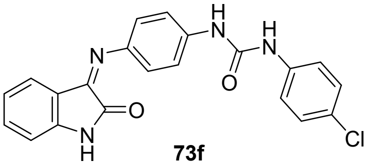

73f

IC_{50} = 3.12 ± 0.18, 2.51 ± 0.09 μM
for HepG2 cell line and VEGFR-2,
respectively

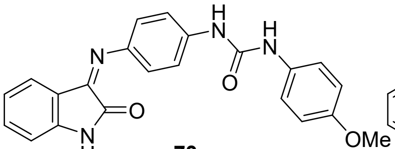

73g

IC_{50} = >50 μM
for HepG2 cell line

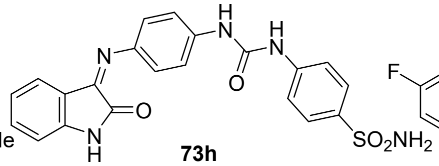

73h

IC_{50} = 11.68 ± 1.56 μM
for HepG2 cell line

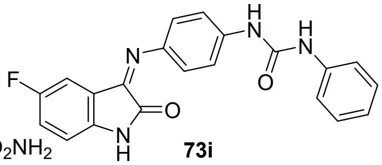

73i

IC_{50} = 22.54 ± 1.25 μM
for HepG2 cell line

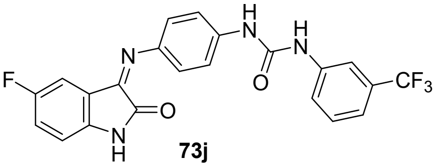

73j

IC_{50} = 2.67 ± 0.28, 2.12 ± 0.33 μM
for HepG2 cell line and VEGFR-2,
respectively

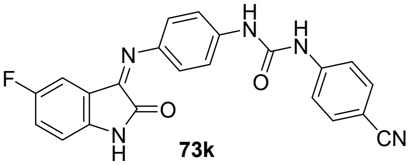

73k

IC_{50} = >50 μM
for HepG2 cell line

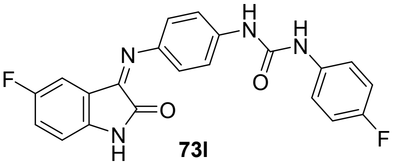

73l

IC_{50} = 30.70 ± 2.97 μM
for HepG2 cell line

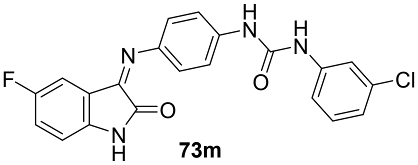

73m

IC_{50} = 2.29 ± 0.38, 1.99 ± 0.11 μM
for HepG2 cell line and VEGFR-2,
respectively

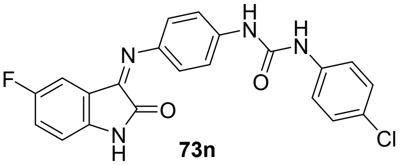

73n

IC_{50} = 1.81 ± 0.14, 1.93 ± 0.07 μM
for HepG2 cell line and VEGFR-2,
respectively

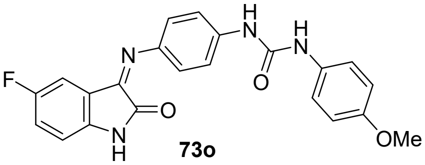

73o

IC_{50} = 2.79 ± 0.29, 2.67 ± 0.22 μM
for HepG2 cell line and VEGFR-2,
respectively

Fig. S4. Antiproliferation and VEGFR-2 properties (μM ± SD) of the prepared Schiff bases **73a–x** and standard references (Doxorubicin and Sorafenib).

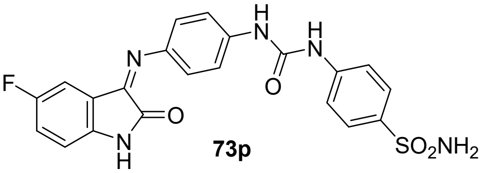

73p

IC_{50} = 13.83 ± 1.45 μM
for HepG2 cell line

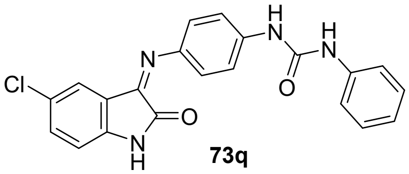

73q

IC_{50} = 21.37 ± 2.58 μM
for HepG2 cell line

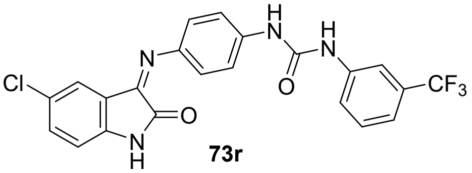

73r

IC_{50} = >50 μM
for HepG2 cell line

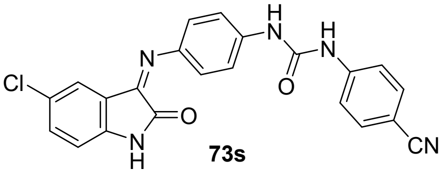

73s

IC_{50} = 10.26 ± 1.39 μM
for HepG2 cell line

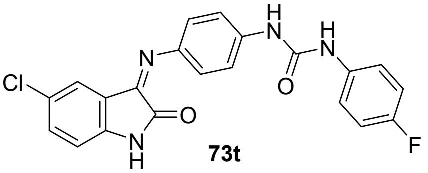

73t

IC_{50} = 3.19 ± 0.97, 3.92 ± 0.46 μM
for HepG2 cell line and VEGFR-2,
respectively

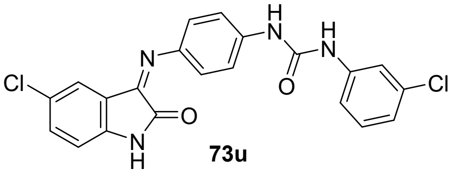

73u

IC_{50} = 2.96 ± 0.35, 0.75 ± 0.05 μM
for HepG2 cell line and VEGFR-2,
respectively

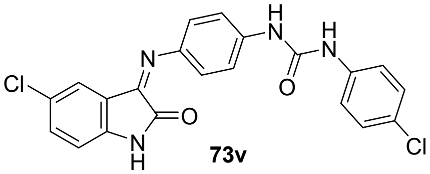

73v

IC_{50} = 8.37 ± 0.85, 0.71 ± 0.08 μM
for HepG2 cell line and VEGFR-2,
respectively

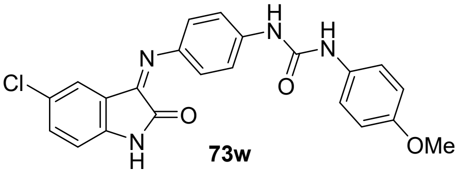

73w

IC_{50} = IC50 = 22.22 ± 3.09 μM
for HepG2 cell line

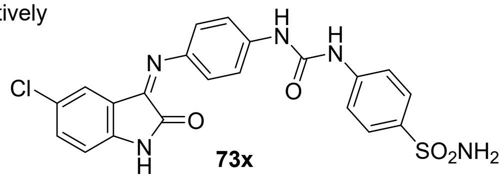

73x

IC_{50} = 3.15 ± 0.36, 0.31 ± 0.04 μM
for HepG2 cell line and VEGFR-2,
respectively

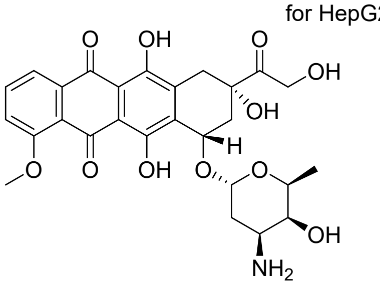

Doxorubicin
IC_{50} = 2.90 ± 0.36 μM for HepG2 cell line

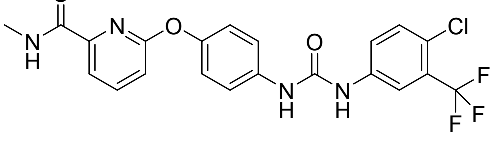

Sorafenib
IC_{50} = 3.40 ± 0.25, 0.10 ± 0.02 μM for
HepG2 cell line and VEGFR-2, respectively

Fig. S4 (continued). Antiproliferation and VEGFR-2 properties (μM ± SD) of the prepared Schiff bases **73a–x** and standard references (Doxorubicin and Sorafenib).

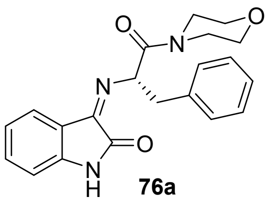

76a

IC_{50} = >50.000 ± 1.52,
>50.00 ±1.85, >50.000 ± 3.16
against MCF7, HCT116
and PaCa2 cell lines, respectively

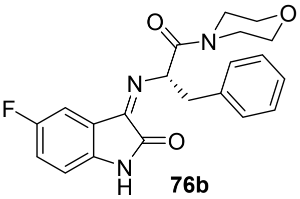

76b

IC_{50} = 45.870 ± 2.32,
>50.00 ± 2.10, >50.000 ± 2.98
against MCF7, HCT116
and PaCa2 cell lines, respectively

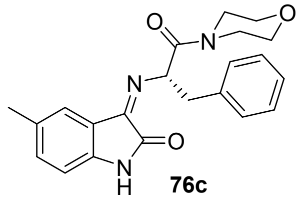

76c

IC_{50} = >50.000 ± 3.33,
>50.00 ± 1.17, >50.000 ± 2.00
against MCF7, HCT116
and PaCa2 cell lines, respectively

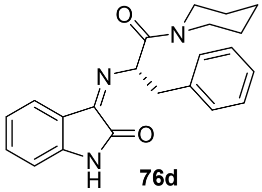

76d

IC_{50} = 39.565 ± 1.64,
>50.00 ± 2.04, 43.617 ± 1.98
against MCF7, HCT116
and PaCa2 cell lines, respectively

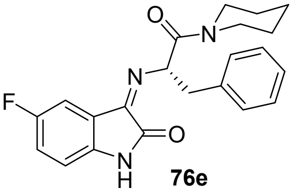

76e

IC_{50} = 39.783 ± 1.92,
>50.00 ± 1.03, 27.021 ± 1.41
against MCF7, HCT116
and PaCa2 cell lines, respectively

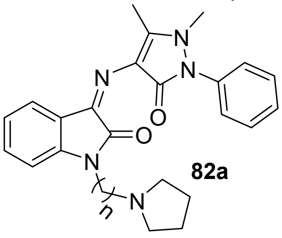

82a

n = 3; IC_{50} = >50.000 ± 3.01,
>50.00 ± 1.92, >50.000 ± 1.84
against MCF7, HCT116
and PaCa2 cell lines, respectively

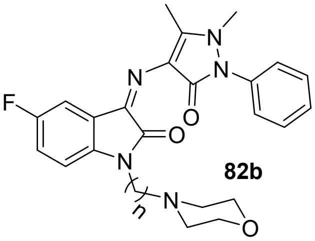

82b

n = 6; IC_{50} = 30.217 ± 1.11,
43.04 ± 1.02, 38.404 ±1.62
against MCF7, HCT116
and PaCa2 cell lines, respectively

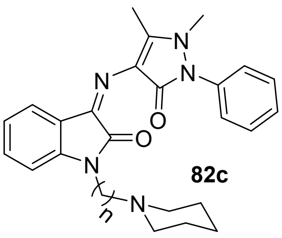

82c

n = 6; IC_{50} = >50.000 ± 2.85,
>50.00 ± 1.99, >50.000 ± 2.26
against MCF7, HCT116
and PaCa2 cell lines, respectively

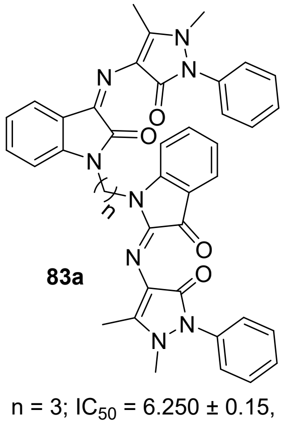

83a

n = 3; IC_{50} = 6.250 ± 0.15,
42.87 ± 1.46, 31.489 ± 1.83
against MCF7, HCT116
and PaCa2 cell lines, respectively;
% inhibition = 65.2 ± 4.1 gainst
VEGFR-2 utilizing IC_{50}
value observed against MCF7 cell line

Fig. S5. Antiproliferation properties (μM ± SEM) and % inhibition of VEGFR-2 for the synthesized agents and standard references "Sunitinib and 5-Fluorouracil".

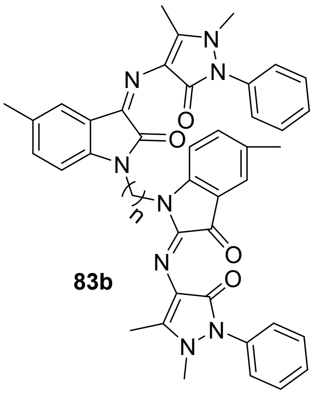

83b

n = 3; IC$_{50}$ = 43.830 ± 2.47,
>50.00 ± 1.02, 40.426 ± 1.04
against MCF7, HCT116
and PaCa2 cell lines, respectively

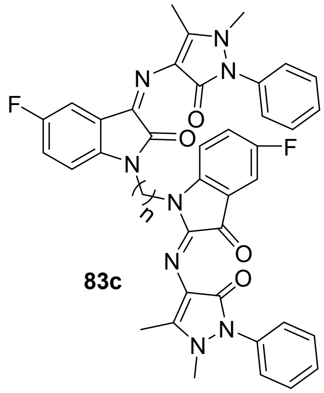

83c

n = 6; IC$_{50}$ = >50.000 ± 1.68,
>50.00 ± 1.16, 43.085 ± 1.41
against MCF7, HCT116
and PaCa2 cell lines, respectively

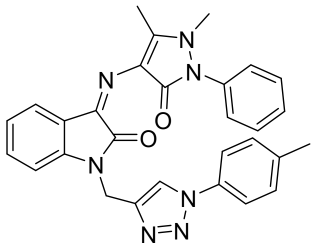

88a

IC$_{50}$ = 27.609 ± 1.37,
>50.00 ± 1.84, >50.000 ± 0.92
against MCF7, HCT116
and PaCa2 cell lines, respectively

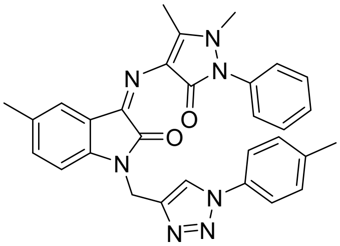

88b

IC$_{50}$ = 5.819 ± 0.23,
17.87 ± 1.11, 25.000 ± 1.31
against MCF7, HCT116
and PaCa2 cell lines, respectively;
% inhibition = 79.2 ± 3.9 gainst
VEGFR-2 utilizing IC$_{50}$
value observed against MCF7 cell line

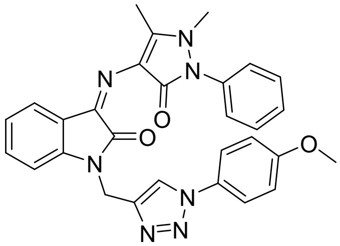

88c

IC$_{50}$ = 12.500 ± 0.96,
45.43 ± 2.02, 37.766 ± 1.66
against MCF7, HCT116
and PaCa2 cell lines, respectively

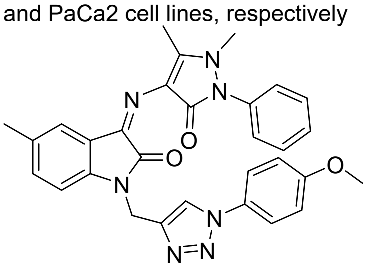

88d

IC$_{50}$ = 8.936 ± 0.19,
16.30 ± 0.99, 16.489 ± 0.83
against MCF7, HCT116
and PaCa2 cell lines, respectively;
% inhibition = 57.9 ± 5.0 gainst
VEGFR-2 utilizing IC$_{50}$
value observed against MCF7 cell line

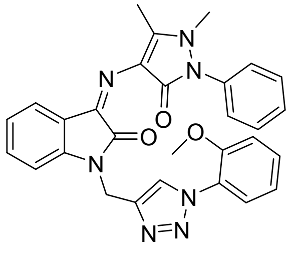

88e

IC$_{50}$ = 25.870 ± 1.47,
>50.00 ± 1.86, 50.000 ± 1.48
against MCF7, HCT116
and PaCa2 cell lines, respectively

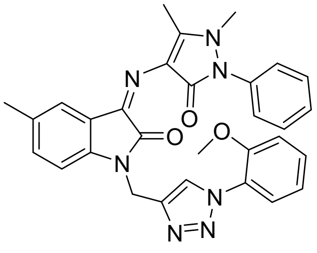

88f

IC$_{50}$ = 5.361 ± 0.31,
12.50 ± 0.88, 12.128 ± 0.79
against MCF7, HCT116
and PaCa2 cell lines, respectively;
% inhibition = 77.6 ± 4.4 gainst
VEGFR-2 utilizing IC$_{50}$
value observed against MCF7 cell line

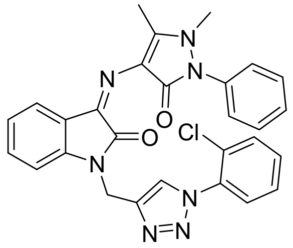

88g

IC$_{50}$ = 21.702 ± 1.64,
>50.00 ± 1.01, >50.000 ± 1.17
against MCF7, HCT116
and PaCa2 cell lines, respectively

Fig. S5 (continued). Antiproliferation properties (μM ± SEM) and % inhibition of VEGFR-2 for the synthesized agents and standard references "Sunitinib and 5-Fluorouracil".

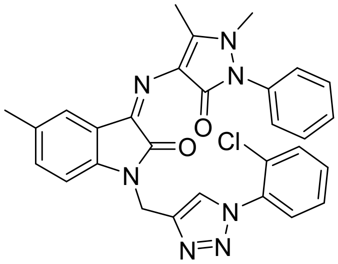

88h

IC_{50} = 7.660 ± 0.13,
22.13 ± 2.63, 17.553 ± 0.84
against MCF7, HCT116
and PaCa2 cell lines, respectively

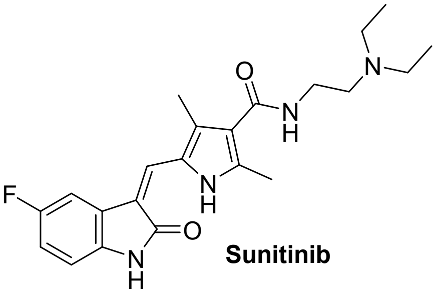

Sunitinib

IC_{50} = 11.304 ± 0.28,
9.67 ± 0.84, 6.596 ± 0.43
against MCF7, HCT116
and PaCa2 cell lines, respectively;
% inhibition = 67.1 ± 3.3 gainst
VEGFR-2 utilizing IC_{50}
value observed against MCF7 cell line

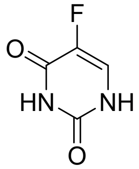

5- Fluorouracil

IC_{50} = 20.43 ± 1.99
against HCT116 cell line

Fig. S5 (continued). Antiproliferation properties (μM ± SEM) and % inhibition of VEGFR-2 for the synthesized agents and standard references "Sunitinib and 5-Fluorouracil".

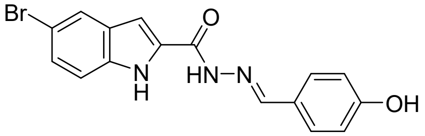

106a

IC_{50} = 56.6 ± 2.4, 61.8 ± 3.2, 90.1 ± 1.9 μM against HepG2, HeLa and PC3, respectively; EC_{50} = 195.0 ± 2.3 nM ± SEM against VEGFR-2

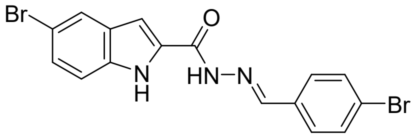

106b

IC_{50} = 68.5 ± 2.2, 40.1 ± 3.0, 69.8 ± 2.7 μM against HepG2, HeLa and PC3, respectively; EC_{50} = 200.3 ± 2.5 nM ± SEM against VEGFR-2

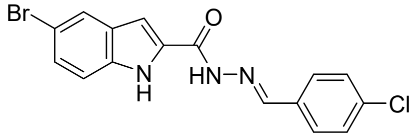

106c

IC_{50} = 92.9 ± 2.2, 63.4 ± 2.1, 95.5 ± 2.2 μM against HepG2, HeLa and PC3, respectively; EC_{50} = 309.3 ± 3.2 nM ± SEM against VEGFR-2

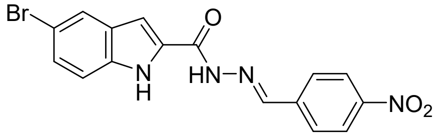

106d

IC_{50} = 37.6 ± 3.0, 41.6 ± 2.3, 119.5 ± 3.6 μM against HepG2, HeLa and PC3, respectively; EC_{50} = 321.5 ± 2.3 nM ± SEM against VEGFR-2

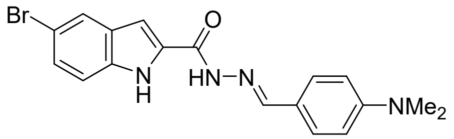

106e

IC_{50} = 14.3 ± 2.0, 22.2 ± 2.3, 36.2 ± 3.1, 25.9 ± 2.1 μM against HepG2, HeLa, PC3 and WI-38, respectively; EC_{50} = 102.6 ± 3.1 nM ± SEM against VEGFR-2

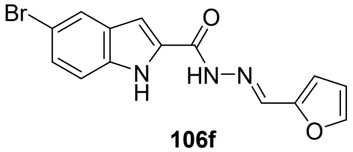

106f

IC_{50} = 60.7 ± 3.3, 67.4 ± 2.0, 109.3 ± 2.2 μM against HepG2, HeLa and PC3, respectively; EC_{50} = 192.6 ± 3.1 nM ± SEM against VEGFR-2

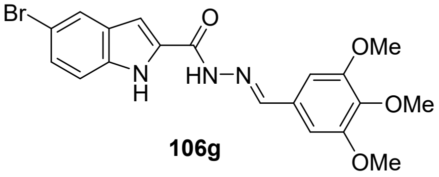

106g

IC_{50} = 56.4 ± 4.0, 47.0 ± 2.3, 70.3 ± 3.1 μM against HepG2, HeLa and PC3, respectively; EC_{50} = 329.5 ± 3.4 nM ± SEM against VEGFR-2

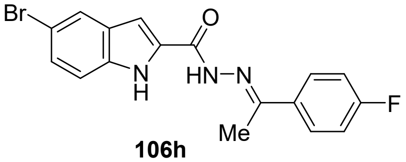

106h

IC_{50} = 32.6 ± 3.9, 35.0 ± 2.7, 37.6 ± 4.0 μM against HepG2, HeLa and PC3, respectively; EC_{50} = 190.9 ± 3.0 nM ± SEM against VEGFR-2

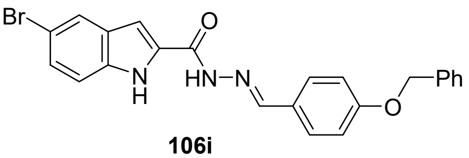

106i

IC_{50} = 70.1 ± 2.1, 71.9 ± 3.2, 113.1 ± 2.7 μM against HepG2, HeLa and PC3, respectively; EC_{50} = 320.0 ± 3.2 nM ± SEM against VEGFR-2

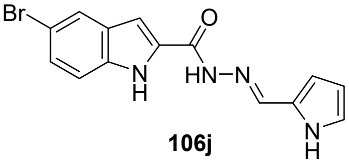

106j

IC_{50} = 38.55 ± 3.1, 39.15 ± 2.3, 43.5 ± 2.6 μM against HepG2, HeLa and PC3, respectively; EC_{50} = 136.1 ± 3.3 nM ± SEM against VEGFR-2

Fig. S6 . Antiproliferation and VEGFR-2 inhibitory properties of hydrazones **106a-k** and Sorafenib.

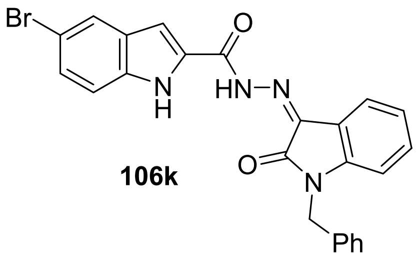

106k

IC$_{50}$ = 118.7 ± 4.3, 105.2 ± 4.2, 144.6 ± 3.4 μM against HepG2, HeLa and PC3, respectively; EC$_{50}$ = 253.8 ± 6.1 nM ± SEM against VEGFR-2

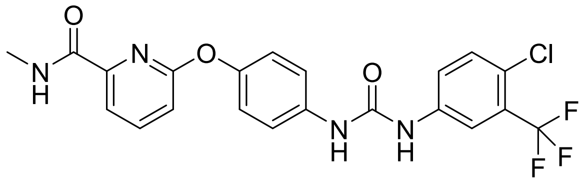

Sorafenib

IC$_{50}$ = 6.2 ± 1.1, 11.7 ± 1.3, 19.0 ± 1.2, 15.3 ± 1.8 μM against HepG2, HeLa, PC3 and WI-38 respectively; EC$_{50}$ = 57.1 ± 3.0 nM ± SEM against VEGFR-2

Fig. S6 (continued). Antiproliferation and VEGFR-2 inhibitory properties of hydrazones **106a**–**k** and Sorafenib.

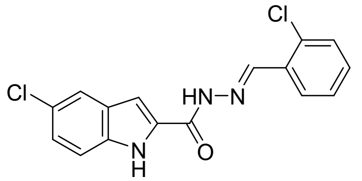

113a

GI_{50} = >100, 89.65 ± 8.13, >100 μM against HCT116, SW480 and MRC-5, respectively

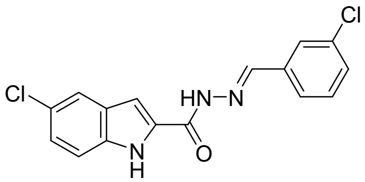

113b

GI_{50} = >100, >100, >100 μM against HCT116, SW480 and MRC-5, respectively

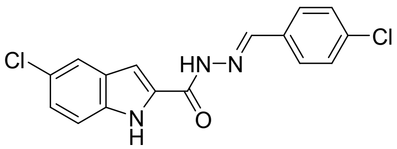

113c

GI_{50} = 8.02 ± 1.3, 7.70 ± 2.1, >100 μM against HCT116, SW480 and MRC-5, respectively

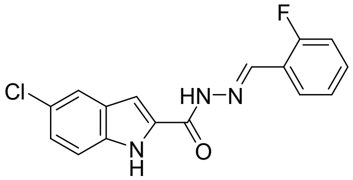

113d

GI_{50} = >100, >100, >100 μM against HCT116, SW480 and MRC-5, respectively

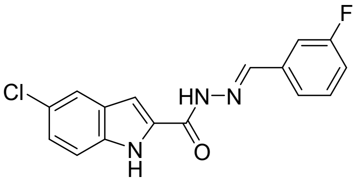

113e

GI_{50} = >100, >100, >100 μM against HCT116, SW480 and MRC-5, respectively

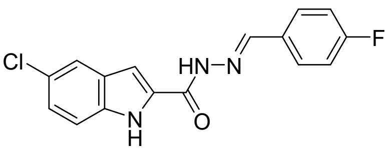

113f

GI_{50} = 54.85 ± 2.6, 65.87 ± 5.3, >100 μM against HCT116, SW480 and MRC-5, respectively

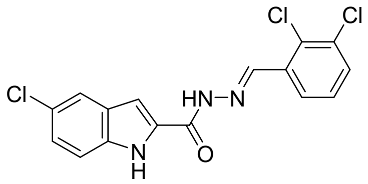

113g

GI_{50} = 26.85 ± 5.0, 31.00 ± 0.1, 42.32 ± 2.1 μM against HCT116, SW480 and MRC-5, respectively

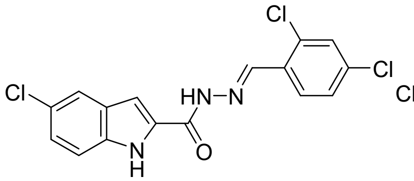

113h

GI_{50} = 15.4 ± 7.6, 14.1 ± 7.9, 41.4 ± 0.68 μM against HCT116, SW480 and MRC-5, respectively

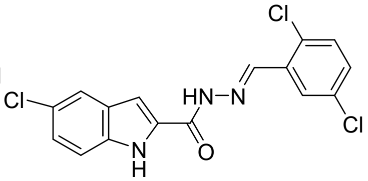

113i

GI_{50} = 26.75 ± 37.8, 39.97 ± 4.5, >100 μM against HCT116, SW480 and MRC-5, respectively

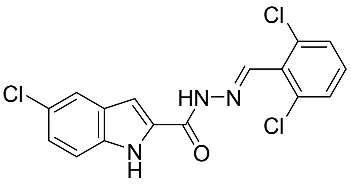

113j

GI_{50} = 5.53 ± 2.9, 7.40 ± 1.4, 21.8 ± 1.1 μM against HCT116, SW480 and MRC-5, respectively

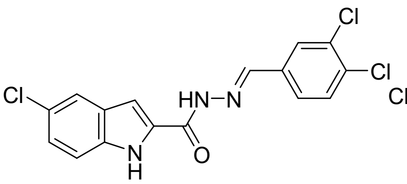

113k

GI_{50} = 40.30 ± 4.4, 45.15 ± 18, >100 μM against HCT116, SW480 and MRC-5, respectively

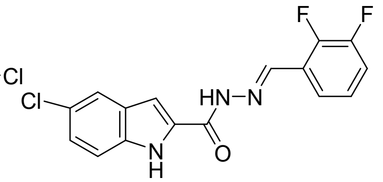

113l

GI_{50} = 47.65 ± 8.6, 59.50 ± 17.1, >100 μM against HCT116, SW480 and MRC-5, respectively

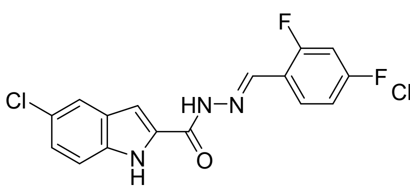

113m

GI_{50} = >100, >100, >100 μM against HCT116, SW480 and MRC-5, respectively

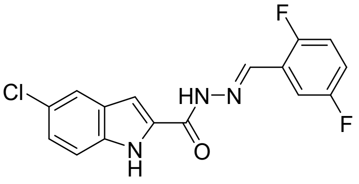

113n

GI_{50} = 8.15 ± 2.3, 8.90 ± 1.6, >100 μM against HCT116, SW480 and MRC-5, respectively

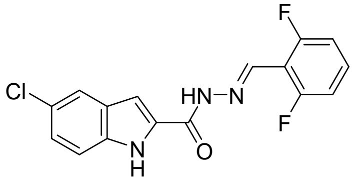

113o

GI_{50} = >100, >100, >100 μM against HCT116, SW480 and MRC-5, respectively

Fig. S7. Antiproliferation properties of indolyl hydrazones **113** and standard references (Cisplatin, Sorafenib and Sunitinib).

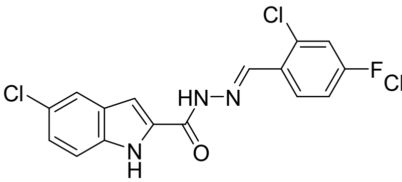

113p

GI_{50} = 37.50 ± 2.12, 59.50 ± 29.0, >100 μM against HCT116, SW480 and MRC-5, respectively

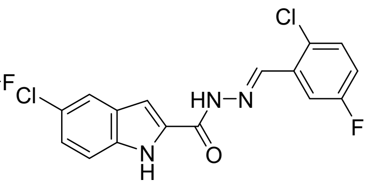

113q

GI_{50} = 4.23 ± 3.1, 0.91 ± 1.0, 10.9 ± 0.6 μM against HCT116, SW480 and MRC-5, respectively

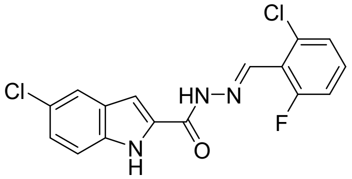

113r

GI_{50} = 9.03 ± 1.6, 9.90 ± 1.8, 50.9 ± 16.9 μM against HCT116, SW480 and MRC-5, respectively

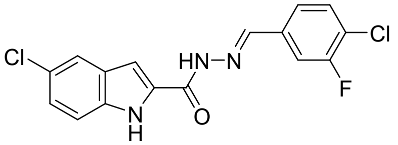

113s

GI_{50} = >100, >100, >100 μM against HCT116, SW480 and MRC-5, respectively

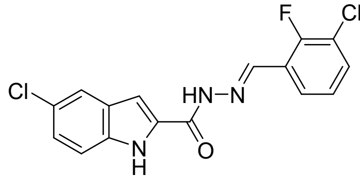

113t

GI_{50} = 0.21 ± 7.7, 40.02 ± 5.90, >100 μM against HCT116, SW480 and MRC-5, respectively

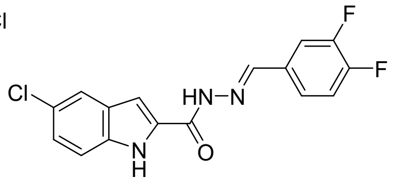

113u

GI_{50} = 84.85 ± 2.3, >100, >100 μM against HCT116, SW480 and MRC-5, respectively

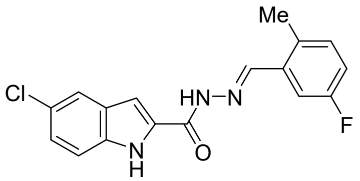

113v

GI_{50} = 8.73 ± 0.6, 8.55 ± 0.1, >100 μM against HCT116, SW480 and MRC-5, respectively

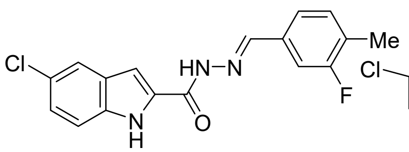

113w

GI_{50} = 25.90 ± 16.0, 8.75 ± 1.8, >100 μM against HCT116, SW480 and MRC-5, respectively

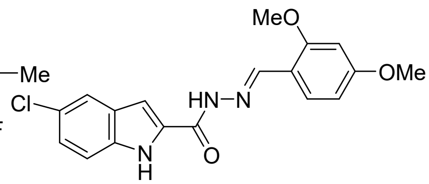

113x

GI_{50} = 8.10 ± 1.8, 7.90 ± 1.3, >100 μM against HCT116, SW480 and MRC-5, respectively

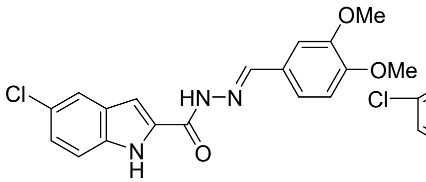

113y

GI_{50} = 63.21 ± 12.0, 72.43 ± 9.0, >100 μM against HCT116, SW480 and MRC-5, respectively

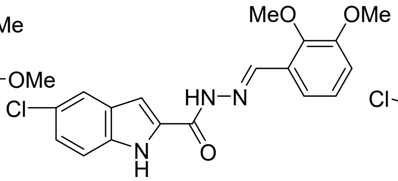

113z

GI_{50} = 52.70 ± 16.0, 63.95 ± 12.8, >100 μM against HCT116, SW480 and MRC-5, respectively

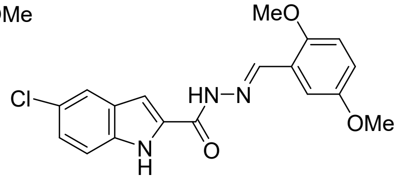

113aa

GI_{50} = 47.7 ± 0.4, 51.7 ± 2.3, 81.8 ± 11.6 μM against HCT116, SW480 and MRC-5, respectively

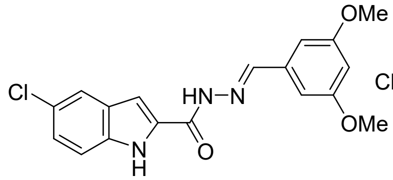

113ab

GI_{50} = 8.87 ± 2.3, 8.17 ± 1.6, 71.1 ± 1.6 μM against HCT116, SW480 and MRC-5, respectively

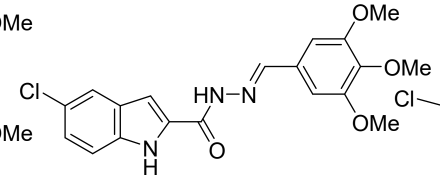

113ac

GI_{50} = > 100, > 100, > 100 μM against HCT116, SW480 and MRC-5, respectively

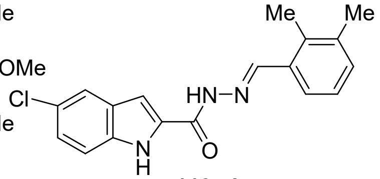

113ad

GI_{50} = 31.31 ± 5.8, 39.6 ± 5.1, 88.42 ± 11.4 μM against HCT116, SW480 and MRC-5, respectively

Fig. S7 (continued). Antiproliferation properties of indolyl hydrazones **113** and standard references (Cisplatin, Sorafenib and Sunitinib).

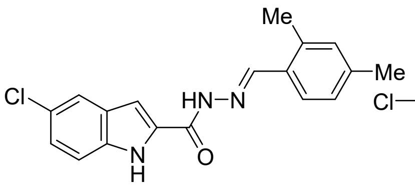

113ae

GI_{50} = 0.79 ± 0.1, 0.70 ± 0.1, 65.30 ± 0.3 μM against HCT116, SW480 and MRC-5, respectively

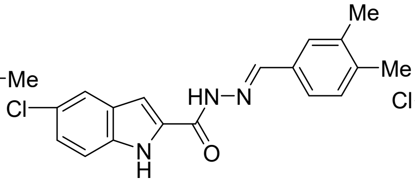

113af

GI_{50} = 11.6 ± 7.8, 7.40 ± 1.3, 10.02 ± 0.04 μM against HCT116, SW480 and MRC-5, respectively

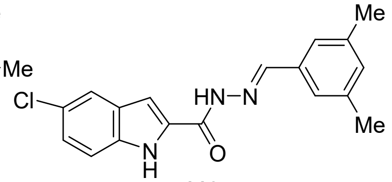

113ag

GI_{50} = 35.10 ± 1.8, 40.45 ± 9.3, > 100 μM against HCT116, SW480 and MRC-5, respectively

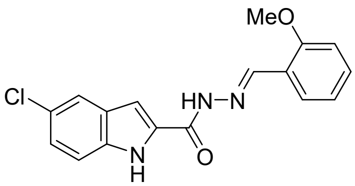

113ah

GI_{50} = 0.93 ± 0.3, 1.70 ± 1.6, 0.94 ± 0.04 μM against HCT116, SW480 and MRC-5, respectively

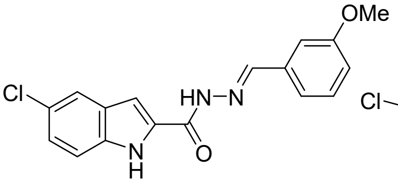

113ai

GI_{50} = 9.47 ± 3.0, 11.9 ± 6.5, > 100 μM against HCT116, SW480 and MRC-5, respectively

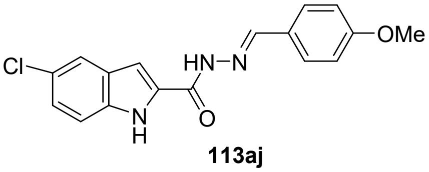

113aj

GI_{50} = > 100, > 100, > 100 μM against HCT116, SW480 and MRC-5, respectively

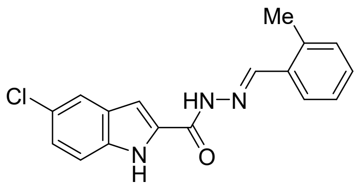

113ak

GI_{50} = 36.15 ± 9.7, 46.40 ± 20.9, > 100 μM against HCT116, SW480 and MRC-5, respectively

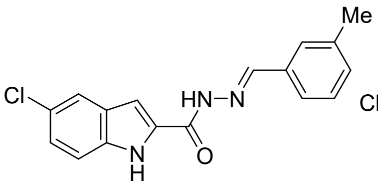

113al

GI_{50} = 44.10 ± 25.7, 44.10 ± 18.0, > 100 μM against HCT116, SW480 and MRC-5, respectively

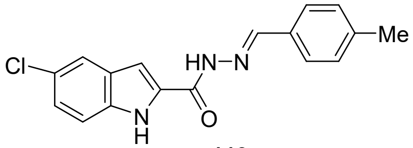

113am

GI_{50} = > 100, > 100, > 100 μM against HCT116, SW480 and MRC-5, respectively

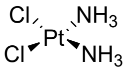

Cisplatin

GI_{50} = 7.67 ± 3.4, 4.43 ± 2.1, 3.82 ± 1.9 μM against HCT116, SW480 and MRC-5, respectively

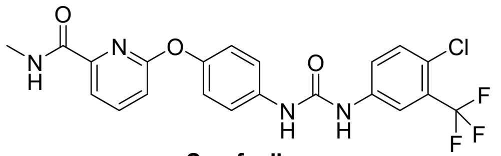

Sorafenib

GI_{50} = 4.17 ± 2.5, 2.02 ± 1.2, 30.81 ± 10.6 μM against HCT116, SW480 and MRC-5, respectively

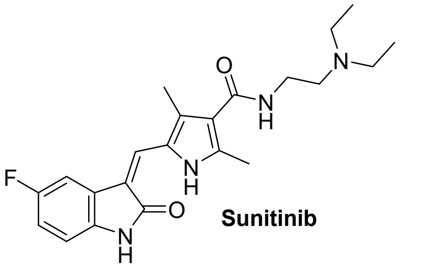

Sunitinib

GI_{50} = 15.84 ± 1.7, 1.09 ± 0.9, > 100 μM against HCT116, SW480 and MRC-5, respectively

Fig. S7 (continued). Antiproliferation properties of indolyl hydrazones **113** and standard references (Cisplatin, Sorafenib and Sunitinib).

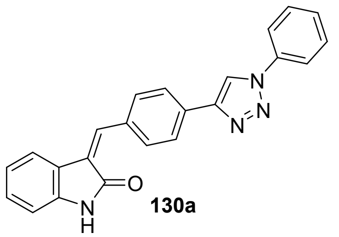

130a

IC_{50} = 7.07 ± 1.10, 11.59 ± 0.81, 12.68 ± 1.69 μM against HT-29, MKN-45 and HUVEC, respectively; IC_{50} = 93.11 ± 1.95 nM against VEGFR-2

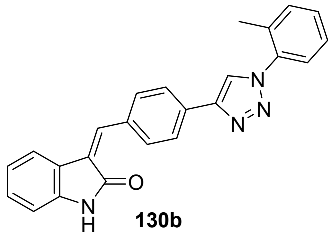

130b

IC_{50} = 8.71 ± 0.48, 9.77 ± 0.86, 14.69 ± 0.93 μM against HT-29, MKN-45 and HUVEC, respectively; IC_{50} = 110.60 ± 2.37 nM against VEGFR-2

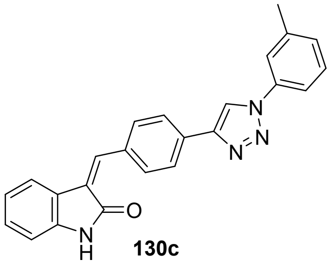

130c

IC_{50} = 3.94 ± 0.38, 4.69 ± 0.24, 13.70 ± 0.13 μM against HT-29, MKN-45 and HUVEC, respectively; IC_{50} = 44.67 ± 0.94 nM against VEGFR-2

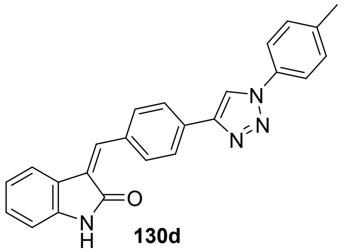

130d

IC_{50} = 1.61 ± 0.45, 1.92 ± 0.37, 7.94 ± 0.36 μM against HT-29, MKN-45 and HUVEC, respectively; IC_{50} = 26.38 ± 1.09 nM against VEGFR-2

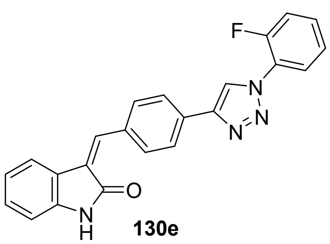

130e

IC_{50} = 10.53 ± 0.79, 12.54 ± 0.97, 19.26 ± 1.07 μM against HT-29, MKN-45 and HUVEC, respectively; IC_{50} = 115.42 ± 2.37 nM against VEGFR-2

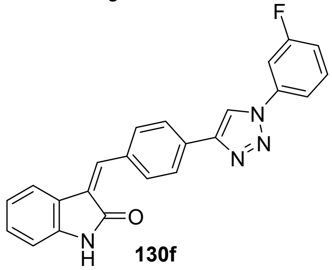

130f

IC_{50} = >20, >20, >20 μM against HT-29, MKN-45 and HUVEC, respectively; IC_{50} = >200 nM against VEGFR-2

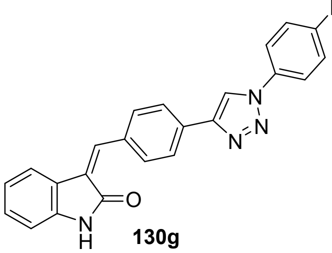

130g

IC_{50} = 17.73 ± 3.80, 16.70 ± 0.82, 14.71 ± 0.23 μM against HT-29, MKN-45 and HUVEC, respectively; IC_{50} = 198.54 ± 1.37 nM against VEGFR-2

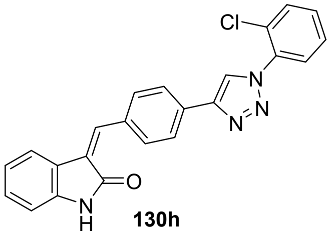

130h

IC_{50} = >20, >20, >20 μM against HT-29, MKN-45 and HUVEC, respectively; IC_{50} = >200 nM against VEGFR-2

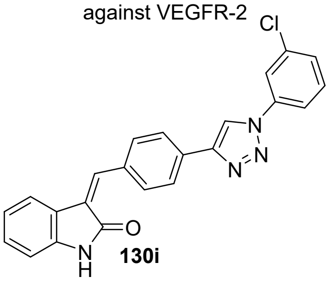

130i

IC_{50} = >20, >20, >20 μM against HT-29, MKN-45 and HUVEC, respectively; IC_{50} = >200 nM against VEGFR-2

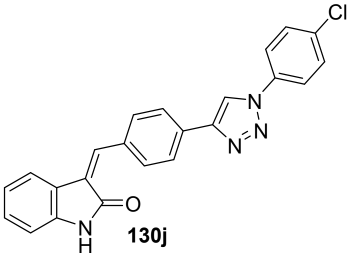

130j

IC_{50} = 13.99 ± 0.41, 16.21 ± 0.99, 18.24 ± 0.58 μM against HT-29, MKN-45 and HUVEC, respectively; IC_{50} = 162.81 ± 2.36 nM against VEGFR-2

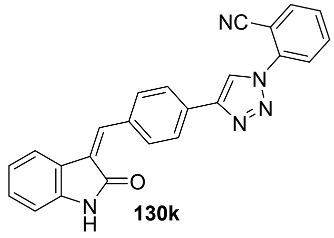

130k

IC_{50} = 14.39 ± 1.44, 14.63 ± 0.53, 19.72 ± 1.02 μM against HT-29, MKN-45 and HUVEC, respectively; IC_{50} = 169.42 ± 1.45 nM against VEGFR-2

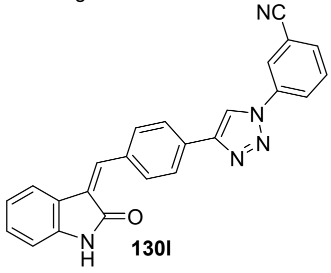

130l

IC_{50} = >20, >20, >20 μM against HT-29, MKN-45 and HUVEC, respectively; IC_{50} = >200 nM against VEGFR-2

Fig S8. Antiproliferation and VEGFR-2 inhibitory properties of 2-oxoindolin-3-ylidenes **130** and Sunitinib.

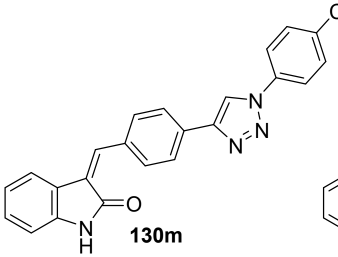

130m

IC_{50} = 15.78 ± 2.55, >20, >20 μM against HT-29, MKN-45 and HUVEC, respectively; IC_{50} = 178.91 ± 1.36 nM against VEGFR-2

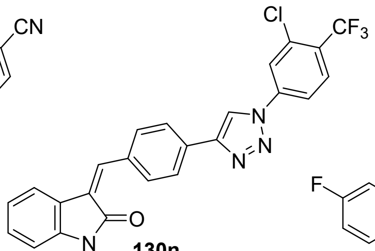

130n

IC_{50} = 9.67 ± 0.39, 8.89 ± 0.42, >20 μM against HT-29, MKN-45 and HUVEC, respectively; IC_{50} = 108.36 ± 0.88 nM against VEGFR-2

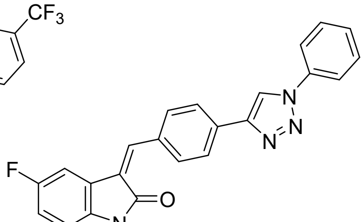

130o

IC_{50} = 9.67 ± 0.39, 8.89 ± 0.42, >20 μM against HT-29, MKN-45 and HUVEC, respectively; IC_{50} = 108.36 ± 0.88 nM against VEGFR-2

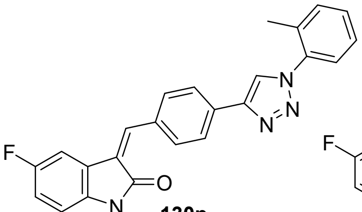

130p

IC_{50} = 5.87 ± 0.32, 7.21 ± 1.90, 8.63 ± 0.27 μM against HT-29, MKN-45 and HUVEC, respectively; IC_{50} = 71.55 ± 0.16 nM against VEGFR-2

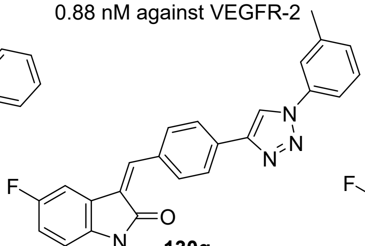

130q

IC_{50} = 7.32 ± 0.64, 6.81 ± 1.49, 9.88 ± 0.76 μM against HT-29, MKN-45 and HUVEC, respectively; IC_{50} = 89.16 ± 0.63 nM against VEGFR-2

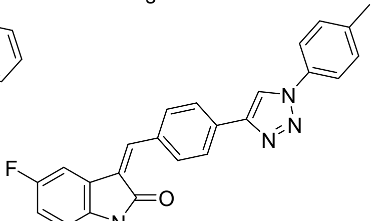

130r

IC_{50} = 3.98 ± 0.61, 34.62 ± 0.34, 13.83 ± 0.42 μM against HT-29, MKN-45 and HUVEC, respectively; IC_{50} = 36.90 ± 0.54 nM against VEGFR-2

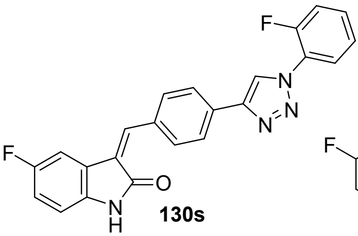

130s

IC_{50} = 8.98 ± 0.42, 8.51 ± 0.54, 14.36 ± 1.99 μM against HT-29, MKN-45 and HUVEC, respectively; IC_{50} = 139.50 ± 1.65 nM against VEGFR-2

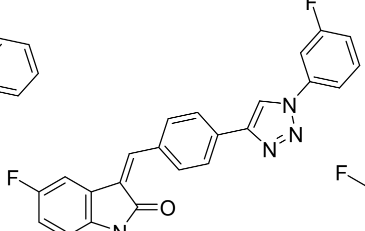

130t

IC_{50} = 12.97 ± 0.72, 8.19 ± 1.30, 17.25 ± 1.22 μM against HT-29, MKN-45 and HUVEC, respectively; IC_{50} = 171.12 ± 1.09 nM against VEGFR-2

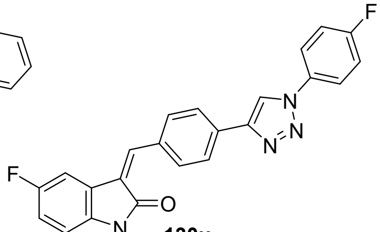

130u

IC_{50} = >20, >20, >20 μM against HT-29, MKN-45 and HUVEC, respectively; IC_{50} = 192.51 ± 0.47 nM against VEGFR-2

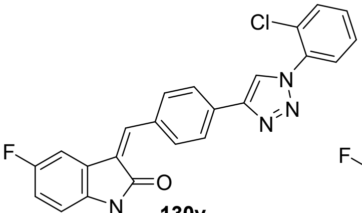

130v

IC_{50} = 5.95 ± 0.27, 7.54 ± 0.55, 18.24 ± 0.58 μM against HT-29, MKN-45 and HUVEC, respectively; IC_{50} = 119.93 ± 1.36 nM against VEGFR-2

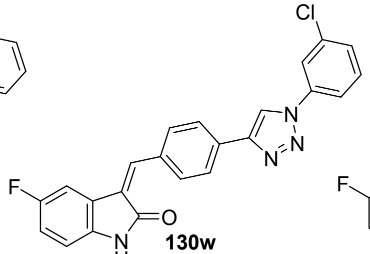

130w

IC_{50} = 10.46 ± 0.43, 14.08 ± 0.88, 19.42 ± 0.59 μM against HT-29, MKN-45 and HUVEC, respectively; IC_{50} = 186.44 ± 1.02 nM against VEGFR-2

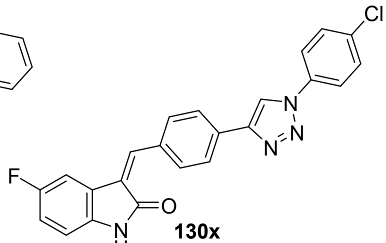

130x

IC_{50} = 11.24 ± 0.86, 15.81 ± 0.52, 19.58 ± 2.14 μM against HT-29, MKN-45 and HUVEC, respectively; IC_{50} = 170.18 ± 2.19 nM against VEGFR-2

Fig S8 (continued). Antiproliferation and VEGFR-2 inhibitory properties of 2-oxoindolin-3-ylidenes **130** and Sunitinib.

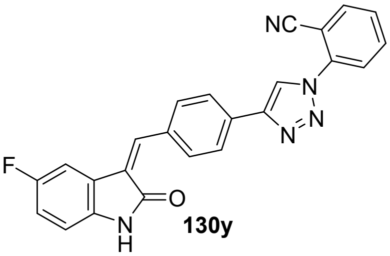

130y

IC$_{50}$ = 13.98 ± 0.43, 15.21 ± 0.73, >20 μM against HT-29, MKN-45 and HUVEC, respectively; IC$_{50}$ = 187.24 ± 2.88 nM against VEGFR-2

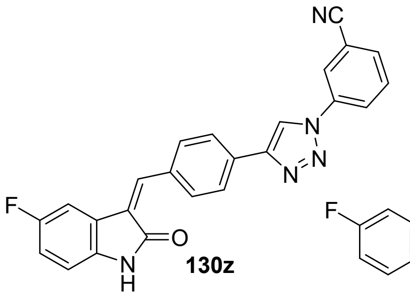

130z

IC$_{50}$ = >20, >20, >20 μM against HT-29, MKN-45 and HUVEC, respectively; IC$_{50}$ = 193.41 ± 0.97 nM against VEGFR-2

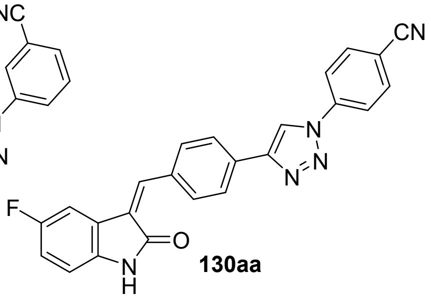

130aa

IC$_{50}$ = >20, >20, >20 μM against HT-29, MKN-45 and HUVEC, respectively; IC$_{50}$ = 190.07 ± 1.98 nM against VEGFR-2

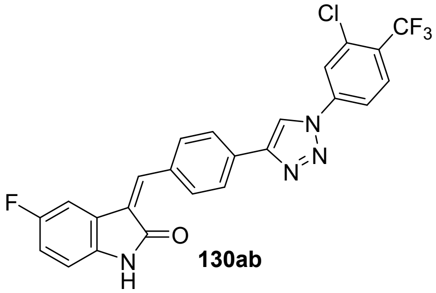

130ab

IC$_{50}$ = 16.52 ± 0.77, >20, >20 μM against HT-29, MKN-45 and HUVEC, respectively; IC$_{50}$ = 168.89 ± 2.34 nM against VEGFR-2

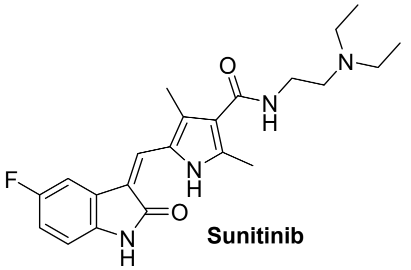

Sunitinib

IC$_{50}$ = 10.34 ± 0.96, 9.25 ± 0.77, 6.37 ± 0.59 μM against HT-29, MKN-45 and HUVEC, respectively; IC$_{50}$ = 83.20 ± 1.36 nM against VEGFR-2.

Fig S8 (continued). Antiproliferation and VEGFR-2 inhibitory properties of 2-oxoindolin-3-ylidenes **130** and Sunitinib.

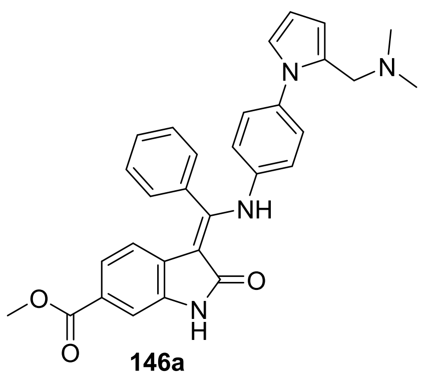

146a

IC_{50} = 51.7, 14.3 nM against VEGFR-2 and PDGFR-β, respectively; IC_{50} = 0.98 ± 0.11, 5.22 ± 0.36, 53.25 ± 1.20 μM against HT-29, SK-OV-3 and HeLa cells, respectively.

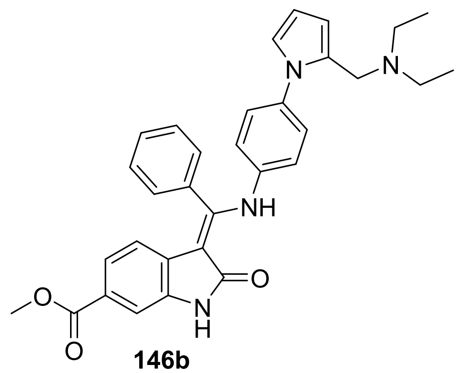

146b

IC_{50} = 164.62, 31.36 nM against VEGFR-2 and PDGFR-β, respectively

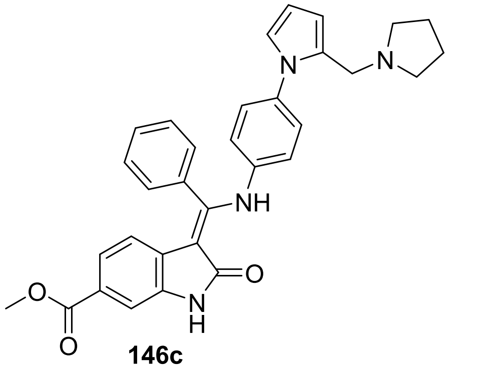

146c

IC_{50} = 152.71, 27.11 nM against VEGFR-2 and PDGFR-β, respectively

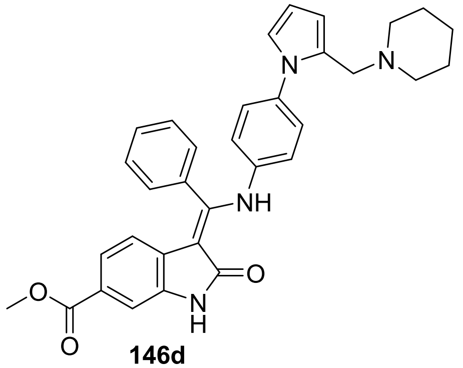

146d

IC_{50} = 137.51, 45.52 nM against VEGFR-2 and PDGFR-β, respectively

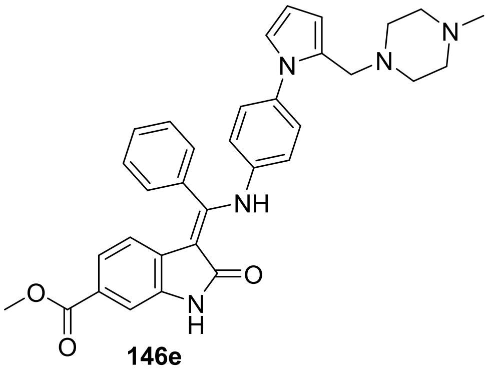

146e

IC_{50} = 38.0, 83.17 nM against VEGFR-2 and PDGFR-β, respectively; IC_{50} = 3.12 ± 0.27, 25.87 ± 1.32, 30.42 ± 1.98 μM against HT-29, SK-OV-3 and HeLa cells, respectively.

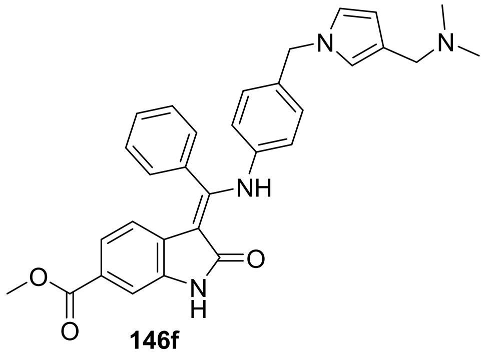

146f

IC_{50} = 167.51, 29.79 nM against VEGFR-2 and PDGFR-β, respectively

Fig. S9. Antiproliferation and enzymatic inhibitory properties of 2-oxoindolin-3-ylidene **146** and Nintedanib

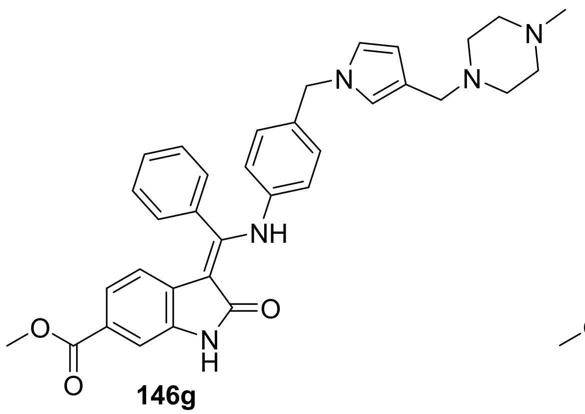

146g

IC_{50} = 164.54, 67.85 nM against VEGFR-2 and PDGFR-β, respectively

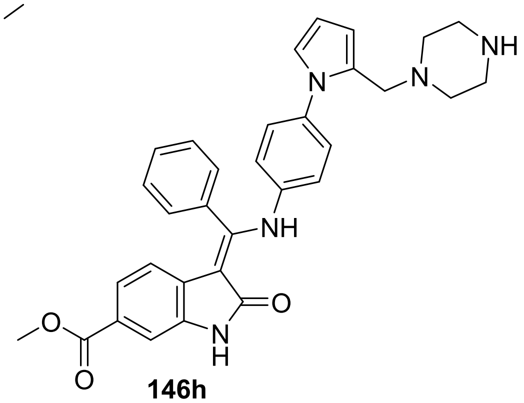

146h

IC_{50} = 96.22, 65.24 nM against VEGFR-2 and PDGFR-β, respectively

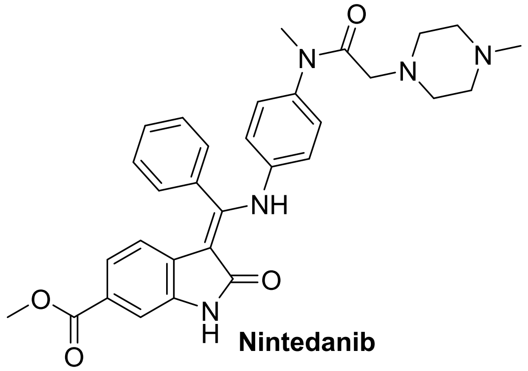

Nintedanib

IC_{50} = 3.3, 3.7 nM against VEGFR-2 and PDGFR-β, respectively; IC_{50} = 4.90 ± 0.65, 28.76 ± 2.13, 51.65 ± 2.68 μM against HT-29, SK-OV-3 and HeLa cells, respectively.

Fig. S9 (continued). Antiproliferation and enzymatic inhibitory properties of 2-oxoindolin-3-ylidene **146** and Nintedanib

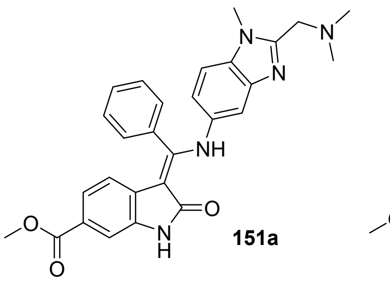

151a

IC_{50} = >100, >100, 89.87 ± 2.31 μM against A549, MCF7 and HT-29, respectively; IC_{50} = 384.7, 45.5 nM against VEGFR-2 and PDGFRβ, respectively

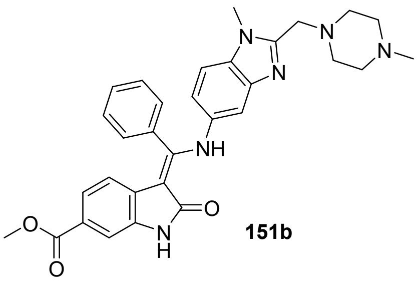

151b

IC_{50} = 40.52 ± 1.62, 22.54 ± 2.16, 0.70 ± 0.12 μM against A549, MCF7 and HT-29, respectively; IC_{50} = 257.2, 61.0 nM against VEGFR-2 and PDGFRβ, respectively

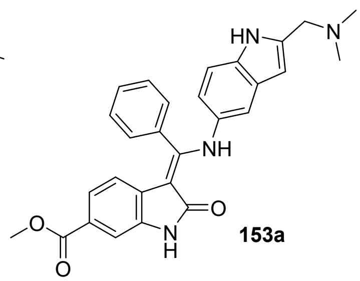

153a

IC_{50} = 16.45 ± 0.88, 2.32 ± 0.23, 0.98 ± 0.26 μM against A549, MCF7 and HT-29, respectively; IC_{50} = 138.3 nM against VEGFR-2

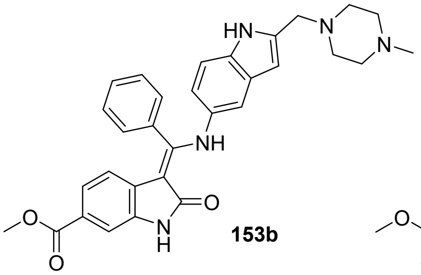

153b

IC_{50} = 39.14 ± 1.28, 3.27 ± 0.52, 0.32 ± 0.03 μM against A549, MCF7 and HT-29, respectively; IC_{50} = 106.1, 92.7 nM against VEGFR-2 and PDGFRβ, respectively

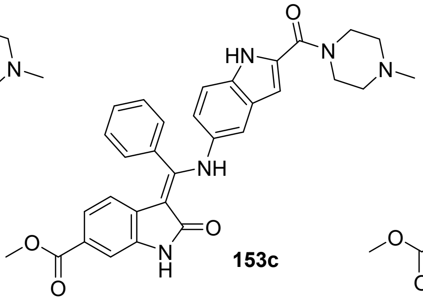

153c

IC_{50} = 2.99 ± 0.22, 1.56 ± 0.53, 1.17 ± 0.18 μM against A549, MCF7 and HT-29, respectively; IC_{50} = 69.1, 22.0 nM against VEGFR-2 and PDGFRβ, respectively

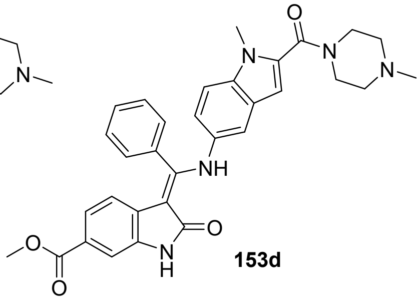

153d

IC_{50} = 75.19 ± 2.79, 1.10 ± 0.02 μM against A549 and HT-29, respectively; IC_{50} = 81.3, 44.2 nM against VEGFR-2 and PDGFRβ, respectively

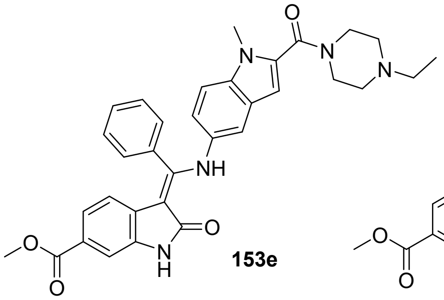

153e

IC_{50} = 11.9 ± 1.17, 2.19 ± 0.23 μM against A549 and HT-29, respectively; IC_{50} = 104.6, 39.0 nM against VEGFR-2 and PDGFRβ, respectively

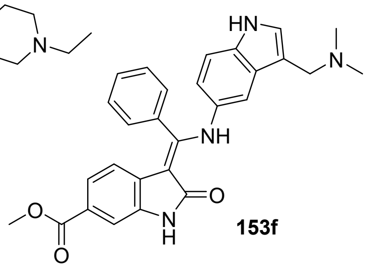

153f

IC_{50} = 2.27 ± 0.58, 3.94 ± 0.76, 0.13 ± 0.03 μM against A549, MCF7 and HT-29, respectively; IC_{50} = >1000, 60.7 nM against VEGFR-2 and PDGFRβ, respectively

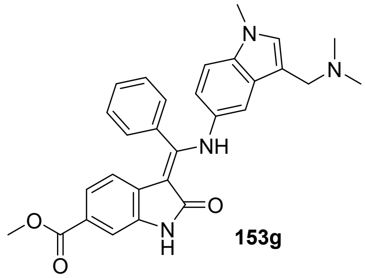

153g

IC_{50} = 6.01 ± 0.33, 2.28 ± 0.50, 0.46 ± 0.05 μM against A549, MCF7 and HT-29, respectively; IC_{50} = 808.5, 64.6 nM against VEGFR-2 and PDGFRβ, respectively

Fig. S10. Antiproliferation and enzymatic inhibitory properties of 2-oxoindolin-3-ylidenes **151**, **153** and Nintedanib.

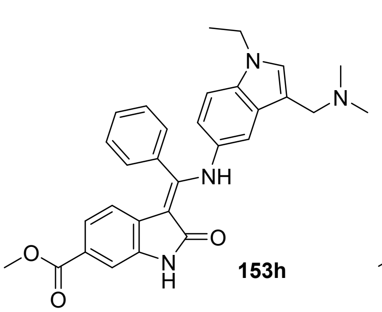

153h

IC_{50} = 7.63 ± 0.69, 5.53 ± 1.33, 6.79 ± 2.07 μM against A549, MCF7 and HT-29, respectively; IC_{50} = 431.2, 34.3 nM against VEGFR-2 and PDGFRβ, respectively

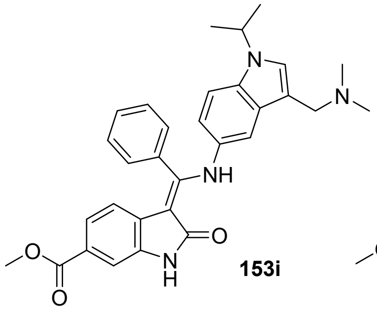

153i

IC_{50} = 4.32 ± 0.06, 5.67 ± 0.78, 0.25 ± 0.05 μM against A549, MCF7 and HT-29, respectively; IC_{50} = 646.5, 49.2 nM against VEGFR-2 and PDGFRβ, respectively

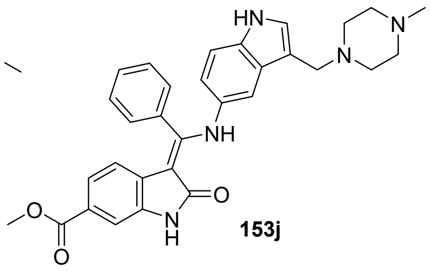

153j

IC_{50} = 7.40 ± 1.32 μM against A549; IC_{50} = 135.1 nM against PDGFRβ; % inhibition = 59 against VEGFR-2 at 1 μM

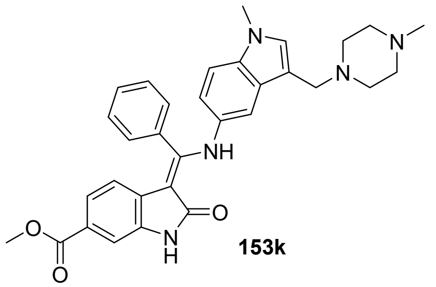

153k

IC_{50} = 6.15 ± 1.07, 4.01 ± 0.68, 0.87 ± 0.12 μM against A549, MCF7 and HT-29, respectively; IC_{50} = >1000, 91.7 nM against VEGFR-2 and PDGFRβ, respectively

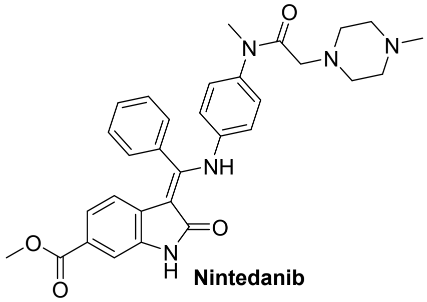

Nintedanib

IC_{50} = 22.62 ± 1.57, 8.28 ± 0.79, 0.83 ± 0.37 μM against A549, MCF7 and HT-29, respectively; IC_{50} = 8.5, 3.5 nM against VEGFR-2 and PDGFRβ, respectively

Fig. S10 (continued). Antiproliferation and enzymatic inhibitory properties of 2-oxoindolin-3-ylidenes **151**, **153** and Nintedanib.

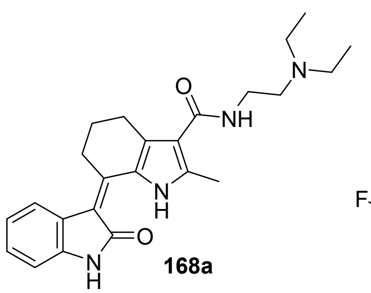

168a

IC_{50} = 9.4, 89.9 nM against VEGFR-2 and PDGFR-β, respectively; IC_{50} = 0.52, 3.56, 6.73, 7.73, 0.54 μM against BXPC-3, T24, BGC, HEPG2 and HT29, respectively

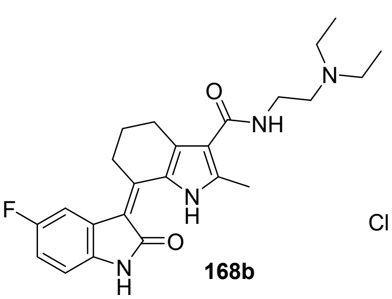

168b

IC_{50} = 2.3, 10.1, 2.4 nM against VEGFR-2, PDGFR-β and c-kit, respectively; IC_{50} = 0.52, 3.31, 6.11, 7.27, 1.2 μM against BXPC-3, T24, BGC, HEPG2 and HT29, respectively

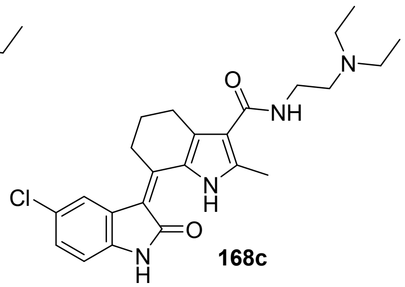

168c

IC_{50} = 2.6, 12.1, 2.7 nM against VEGFR-2, PDGFR-β and c-kit, respectively; IC_{50} = 1.95, 1.83, 2.03, 3.14, 6.48 μM against BXPC-3, T24, BGC, HEPG2 and HT29, respectively

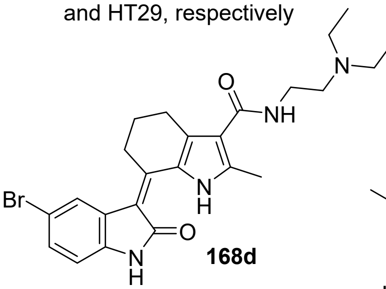

168d

IC_{50} = 3.9, 7.6, 2.3 nM against VEGFR-2, PDGFR-β and c-kit, respectively

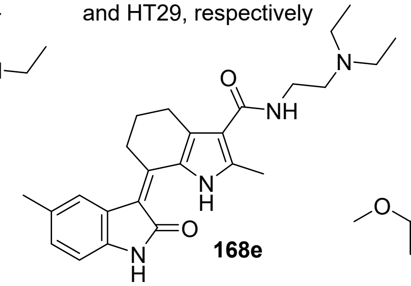

168e

IC_{50} = 2.3, 15.6, 4.1 nM against VEGFR-2, PDGFR-β and c-kit, respectively; IC_{50} = 2.43, 3.15, 1.73 μM against BXPC-3, BGC, and HT29, respectively

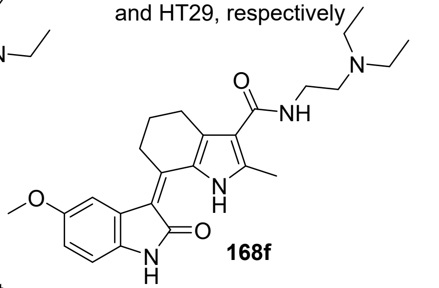

168f

IC_{50} = 24.6, 101.4, 35.5 nM against VEGFR-2, PDGFR-β and c-kit, respectively

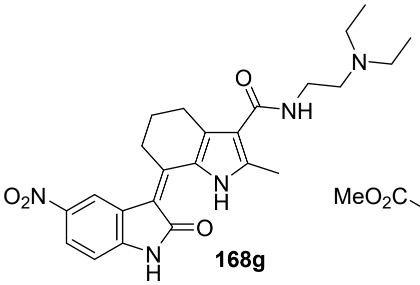

168g

IC_{50} = 25.7, 13.3, 6.9 nM against VEGFR-2, PDGFR-β and c-kit, respectively

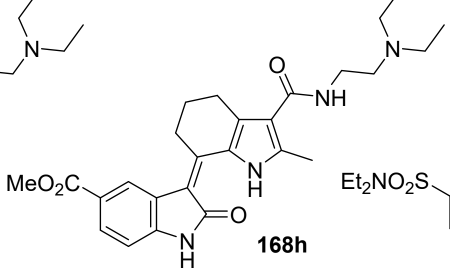

168h

IC_{50} = 167.2, 70.3, 255.7 nM against VEGFR-2, PDGFR-β and c-kit, respectively

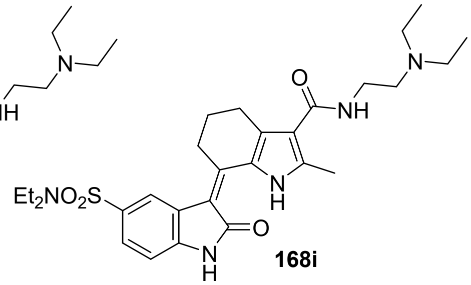

168i

IC_{50} = 330.3, 395.7, 397.6 nM against VEGFR-2, PDGFR-β and c-kit, respectively

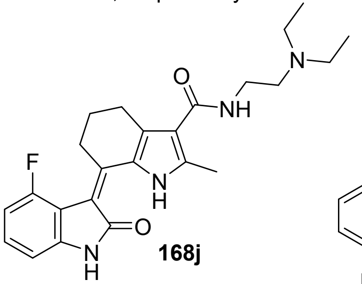

168j

IC_{50} = 15.0, 62.1, 43.1 nM against VEGFR-2, PDGFR-β and c-kit, respectively

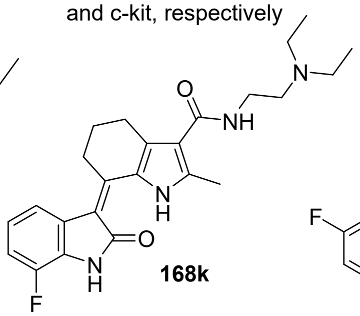

168k

IC_{50} = 112.4, 260.3, 180.8 nM against VEGFR-2, PDGFR-β and c-kit, respectively

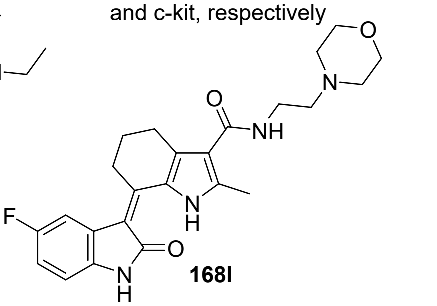

168l

IC_{50} = 1.6, 13.2, 2.0 nM against VEGFR-2, PDGFR-β and c-kit, respectively

Fig. S11. Antiproliferation and enzymatic inhibitory properties of 2-oxoindolin-3-ylidenes **168** and Sunitinib.

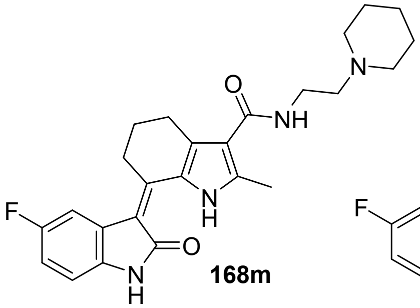

168m

IC_{50} = 4.3, 19.4, 5.8 nM
against VEGFR-2, PDGFR-β
and c-kit, respectively

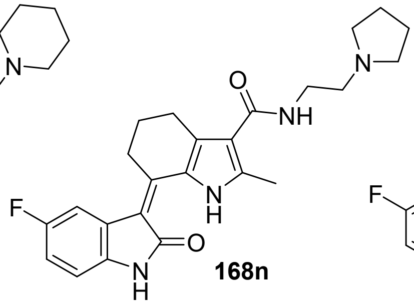

168n

IC_{50} = 9.7, 41.6, 7.4 nM
against VEGFR-2, PDGFR-β
and c-kit, respectively

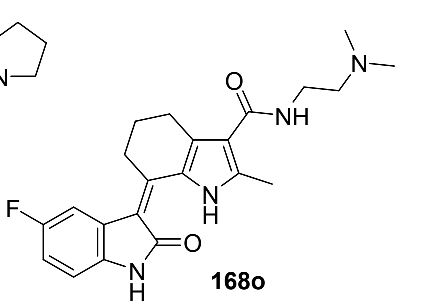

168o

IC_{50} = 3.0, 22.7, 6.6 nM
against VEGFR-2, PDGFR-β
and c-kit, respectively

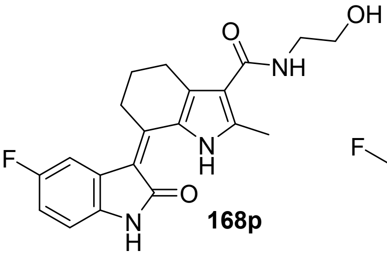

168p

IC_{50} = 1.1, 12.7, 1.4 nM
against VEGFR-2, PDGFR-β
and c-kit, respectively

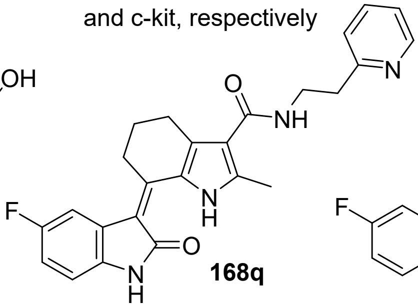

168q

IC_{50} = 11.4, 160.6, 37.7 nM
against VEGFR-2, PDGFR-β
and c-kit, respectively

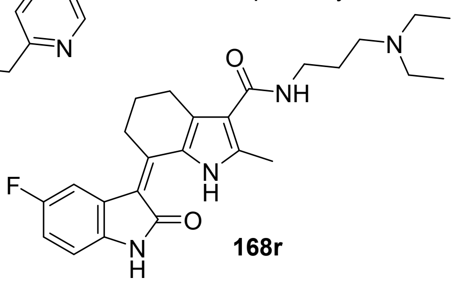

168r

IC_{50} = 2.7, 21.3, 9.9 nM
against VEGFR-2, PDGFR-β
and c-kit, respectively

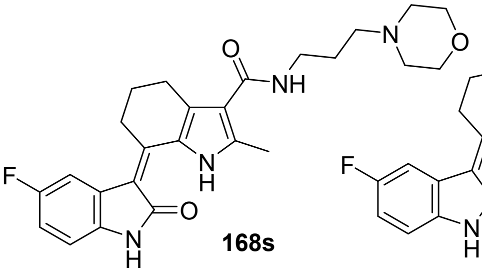

168s

IC_{50} = 1.0, 18.0, 5.7 nM
against VEGFR-2, PDGFR-β
and c-kit, respectively

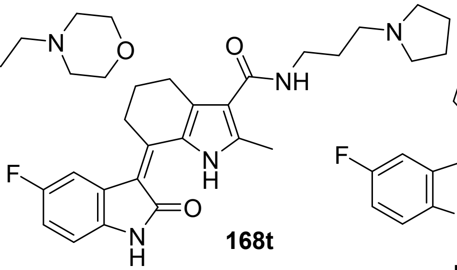

168t

IC_{50} = 3.8, 64.8, 10.7 nM
against VEGFR-2, PDGFR-β
and c-kit, respectively

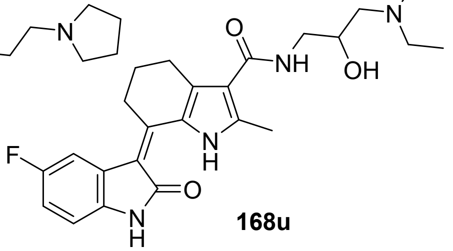

168u

IC_{50} = 2.5, 19.3, 4.1 nM
against VEGFR-2, PDGFR-β
and c-kit, respectively

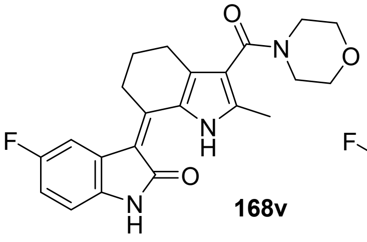

168v

IC_{50} = 1.1, 40.1, 2.1 nM
against VEGFR-2, PDGFR-β
and c-kit, respectively-

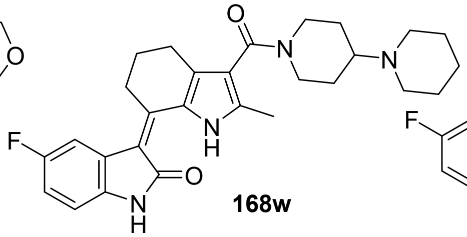

168w

IC_{50} = 8.0, 40.7, 7.8 nM
against VEGFR-2, PDGFR-β
and c-kit, respectively

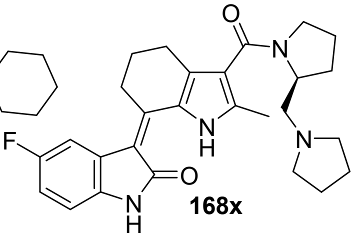

168x

IC_{50} = 11.8, 118.4, 8.5 nM
against VEGFR-2, PDGFR-β
and c-kit, respectively

Fig. S11 (continued). Antiproliferation and enzymatic inhibitory properties of 2-oxoindolin-3-ylidenes **168** and Sunitinib.

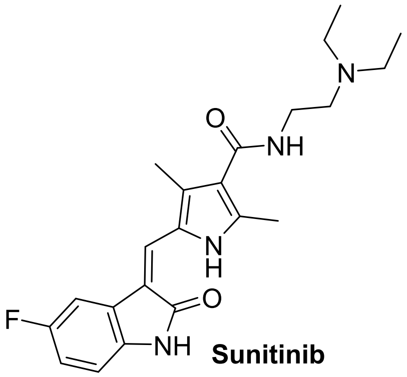

Sunitinib

IC_{50} = 4.0, 10.6, 8.9 nM against VEGFR-2, PDGFR-β and c-kit, respectively; IC_{50} = 3.63, 2.44, 4.78, 5.61, 1.47 μM against BXPC-3, T24, BGC, HEPG2 and HT29, respectively

Fig. S11 (continued). Antiproliferation and enzymatic inhibitory properties of 2-oxoindolin-3-ylidenes **168** and Sunitinib.

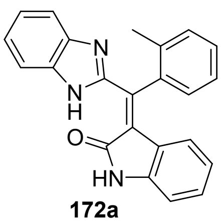

172a

IC_{50} = 618, 97, 205 nM against VEGFR-1, VEGFR-2 and FGFR-1, respectively

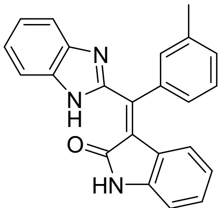

172b

IC_{50} = 681, 119, 814 nM against VEGFR-1, VEGFR-2 and FGFR-1, respectively

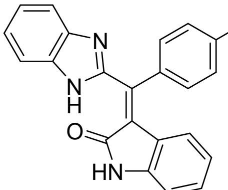

172c

IC_{50} = 599, 88, 187 nM against VEGFR-1, VEGFR-2 and FGFR-1, respectively

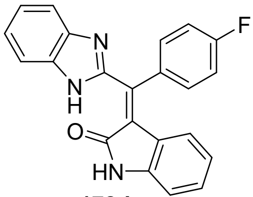

172d

IC_{50} = 2308, 633, 950 nM against VEGFR-1, VEGFR-2 and FGFR-1, respectively

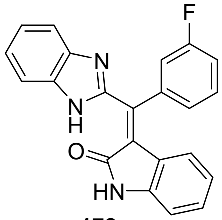

172e

IC_{50} = 522, 125, 79 nM against VEGFR-1, VEGFR-2 and FGFR-1, respectively

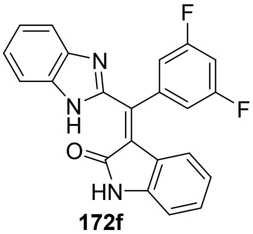

172f

IC_{50} = 2551, 106, 4000 nM against VEGFR-1, VEGFR-2 and FGFR-1, respectively

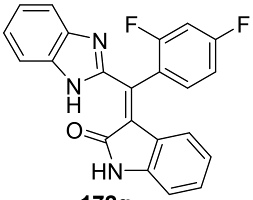

172g

IC_{50} = 1796, 290, 628 nM against VEGFR-1, VEGFR-2 and FGFR-1, respectively

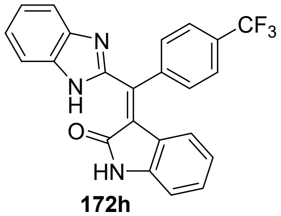

172h

IC_{50} = 24000, 19903, 4899 nM against VEGFR-1, VEGFR-2 and FGFR-1, respectively

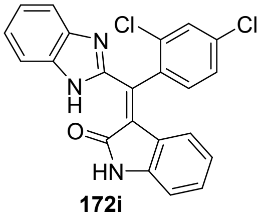

172i

IC_{50} = 24400, 2180, 725 nM against VEGFR-1, VEGFR-2 and FGFR-1, respectively

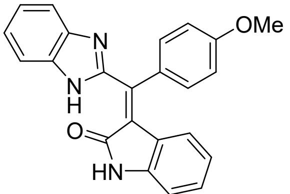

172j

IC_{50} = 984, 327, 86 nM against VEGFR-1, VEGFR-2 and FGFR-1, respectively

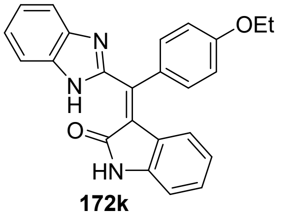

172k

IC_{50} = 10543, 9876, 6293 nM against VEGFR-1, VEGFR-2 and FGFR-1, respectively

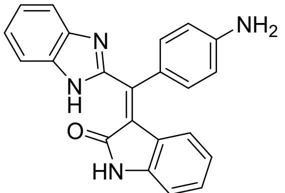

172l

IC_{50} = 371, 68, 57 nM against VEGFR-1, VEGFR-2 and FGFR-1, respectively

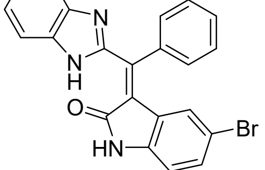

172m

IC_{50} = 118, 51, 446 nM against VEGFR-1, VEGFR-2 and FGFR-1, respectively

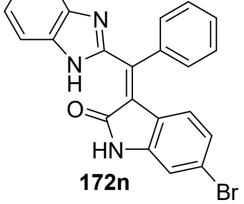

172n

IC_{50} = 4544, 4880, 4880 nM against VEGFR-1, VEGFR-2 and FGFR-1, respectively

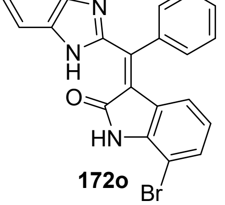

172o

IC_{50} = 4880, 4880, 4880 nM against VEGFR-1, VEGFR-2 and FGFR-1, respectively

Fig. S12. Enzymatic inhibitory properties of 2-oxoindolin-3-ylidenes **172**, **173** and standard references (Sunitinib and SU6668).

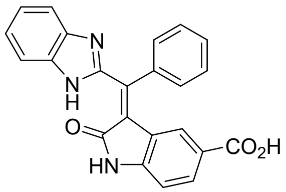

172p

IC_{50} = 39, 4, 75 nM against VEGFR-1, VEGFR-2 and FGFR-1, respectively

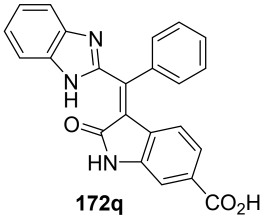

172q

IC_{50} = 582, 582, 1586 nM against VEGFR-1, VEGFR-2 and FGFR-1, respectively

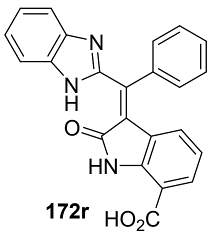

172r

IC_{50} = 20716, 23633, 24400 nM against VEGFR-1, VEGFR-2 and FGFR-1, respectively

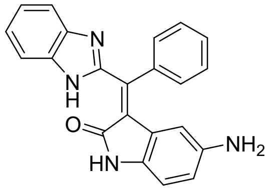

172s

IC_{50} = 201, 32, 13 nM against VEGFR-1, VEGFR-2 and FGFR-1, respectively

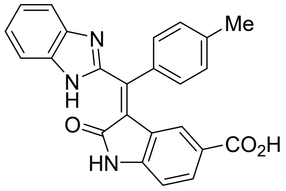

172t

IC_{50} = 45, 5, 67 nM against VEGFR-1, VEGFR-2 and FGFR-1, respectively

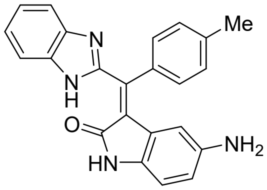

172u

IC_{50} = 473, 82, 51 nM against VEGFR-1, VEGFR-2 and FGFR-1, respectively

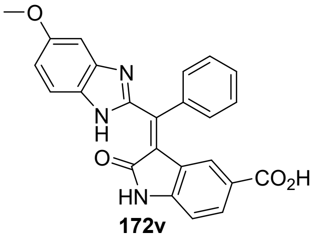

172v

IC_{50} = 77, 3, 39 nM against VEGFR-1, VEGFR-2 and FGFR-1, respectively

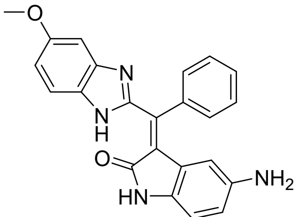

172w

IC_{50} = 377, 60, 42 nM against VEGFR-1, VEGFR-2 and FGFR-1, respectively

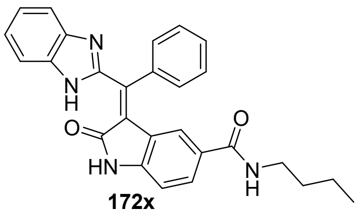

172x

IC_{50} = 374, 15, 326, 13 nM against VEGFR-1, VEGFR-2, FGFR-1 and PDGFRα, respectively

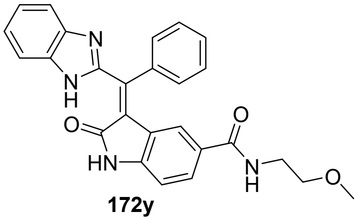

172y

IC_{50} = 381, 69, 199, 55 nM against VEGFR-1, VEGFR-2, FGFR-1 and PDGFRα, respectively

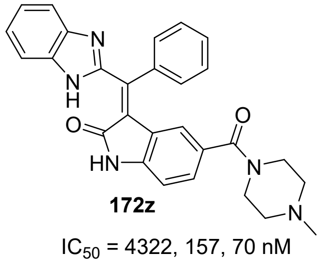

172z

IC_{50} = 4322, 157, 70 nM against VEGFR-1, VEGFR-2 and FGFR-1, respectively

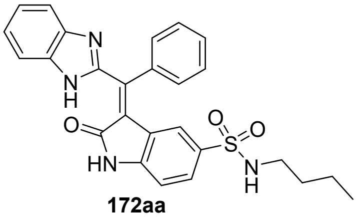

172aa

IC_{50} = 24400, 1134, 300, 844 nM against VEGFR-1, VEGFR-2, FGFR-1 and PDGFRα, respectively

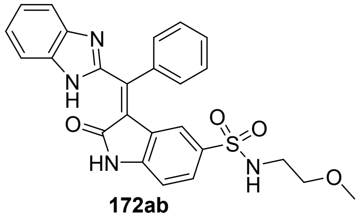

172ab

IC_{50} = 1950, 660, 287, 614 nM against VEGFR-1, VEGFR-2, FGFR-1 and PDGFRα, respectively

Fig. S12 (continued). Enzymatic inhibitory properties of 2-oxoindolin-3-ylidenes **172**, **173** and standard references (Sunitinib and SU6668).

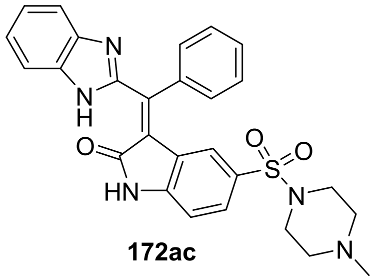

172ac

IC_{50} = 1950, 1950, 1950, 1950 nM
against VEGFR-1, VEGFR-2,
FGFR-1 and PDGFRα, respectively

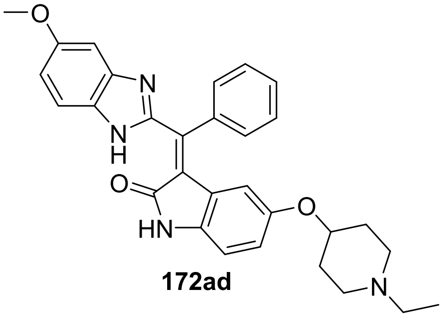

172ad

IC_{50} = 691, 22, 20, 17 nM
against VEGFR-1, VEGFR-2,
FGFR-1 and PDGFRα, respectively

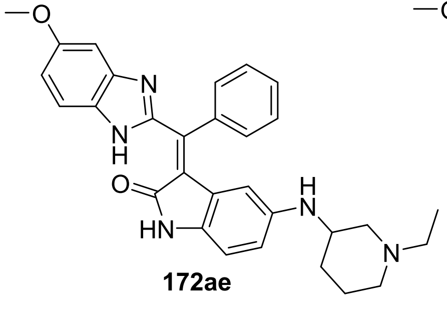

172ae

IC_{50} = >1950, 279, 216, 431 nM
against VEGFR-1, VEGFR-2,
FGFR-1 and PDGFRα, respectively

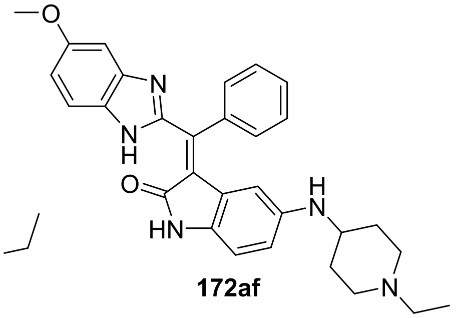

172af

IC_{50} = 163, 10, 11, 6 nM
against VEGFR-1, VEGFR-2,
FGFR-1 and PDGFRα, respectively

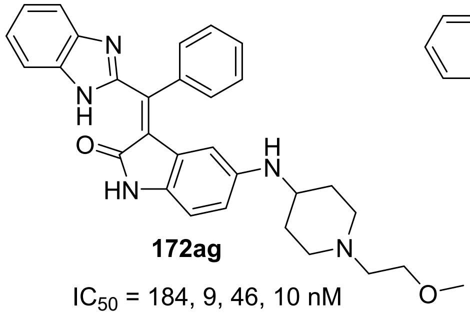

172ag

IC_{50} = 184, 9, 46, 10 nM
against VEGFR-1, VEGFR-2,
FGFR-1 and PDGFRα, respectively

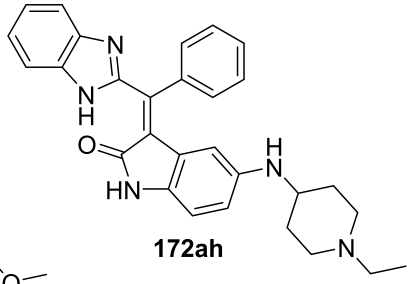

172ah

IC_{50} = 161, 6, 5, 3 nM
against VEGFR-1, VEGFR-2,
FGFR-1 and PDGFRα, respectively

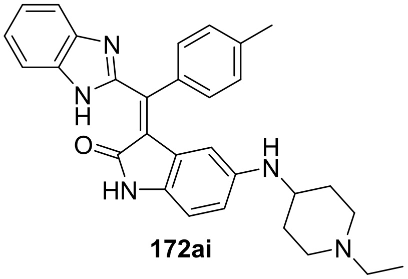

172ai

IC_{50} = 112, 8, 10, 5 nM
against VEGFR-1, VEGFR-2,
FGFR-1 and PDGFRα, respectively

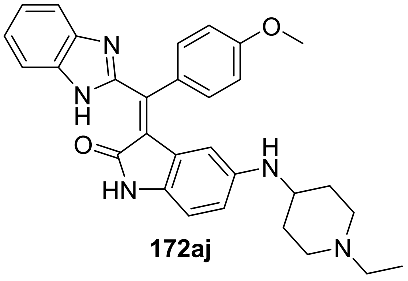

172aj

IC_{50} = 96, 13, 14, 7 nM
against VEGFR-1, VEGFR-2,
FGFR-1 and PDGFRα, respectively

Fig. S12 (continued). Enzymatic inhibitory properties of 2-oxoindolin-3-ylidenes **172**, **173** and standard references (Sunitinib and SU6668).

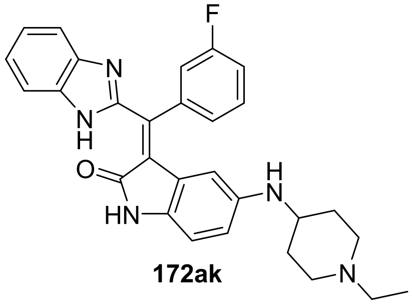

172ak

IC_{50} = 62, 3, 5, 7 nM
against VEGFR-1, VEGFR-2,
FGFR-1 and PDGFRα, respectively

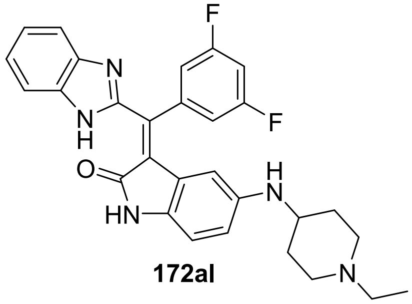

172al

IC_{50} = 66, 4, 8, 6 nM
against VEGFR-1, VEGFR-2,
FGFR-1 and PDGFRα, respectively

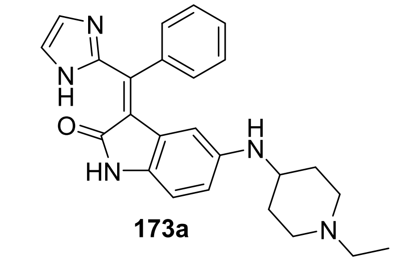

173a

IC_{50} = 166, 16, 12, 20 nM
against VEGFR-1, VEGFR-2,
FGFR-1 and PDGFRα, respectively

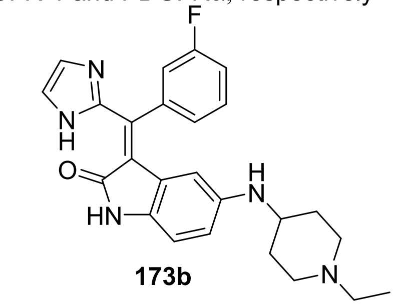

173b

IC_{50} = 25, 7, 13, 6 nM
against VEGFR-1, VEGFR-2,
FGFR-1 and PDGFRα, respectively

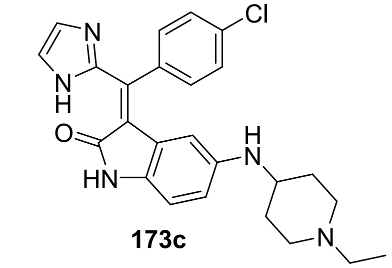

173c

IC_{50} = 7, 4, 6, 3 nM
against VEGFR-1, VEGFR-2,
FGFR-1 and PDGFRα, respectively

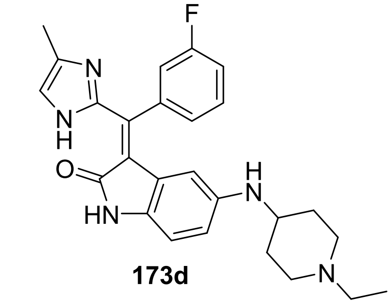

173d

IC_{50} = 20, 4, 4, 2 nM
against VEGFR-1, VEGFR-2,
FGFR-1 and PDGFRα, respectively

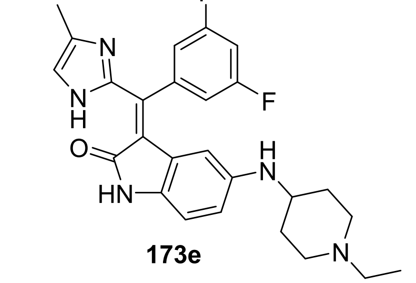

173e

IC_{50} = 11, 3, 7, 3 nM
against VEGFR-1, VEGFR-2,
FGFR-1 and PDGFRα, respectively

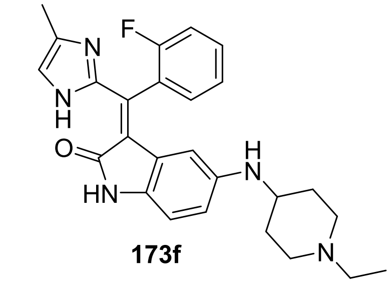

173f

IC_{50} = 29, 3, 8, 5 nM
against VEGFR-1, VEGFR-2,
FGFR-1 and PDGFRα, respectively

Fig. S12 (continued). Enzymatic inhibitory properties of 2-oxoindolin-3-ylidenes **172**, **173** and standard references (Sunitinib and SU6668).

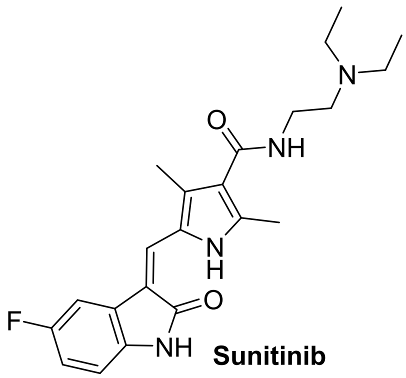

Sunitinib

IC_{50} = 1, 37, 1064 nM
against VEGFR-1, VEGFR-2
and FGFR-1, respectively

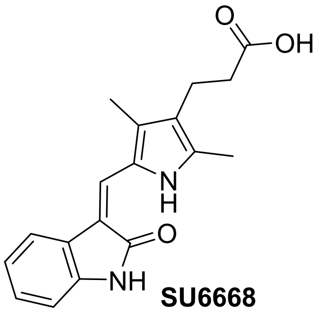

SU6668

IC_{50} = 28, 135, 1950 nM
against VEGFR-1, VEGFR-2
and FGFR-1, respectively

Fig. S12 (continued). Enzymatic inhibitory properties of 2-oxoindolin-3-ylidenes **172**, **173** and standard references (Sunitinib and SU6668).

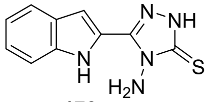

176

IC_{50} = 0.318 ± 0.009 μM against VEGFR-2

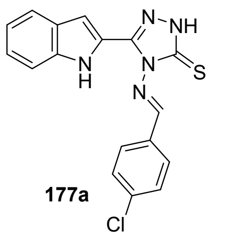

177a

IC_{50} = 0.072 ± 0.002 μM against VEGFR-2; IC_{50} = 9.55 ± 0.44, 5.8 ± 0.27 μM against CAKI-1 and A498 cell lines, respectively

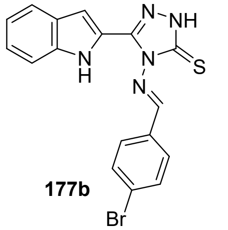

177b

IC_{50} = 0.085 ± 0.002 μM against VEGFR-2; IC_{50} = 0.93 ± 0.04, 26.4 ± 1.22 μM against CAKI-1 and A498 cell lines, respectively

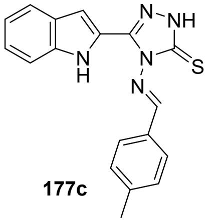

177c

IC_{50} = 0.034 ± 0.001 μM against VEGFR-2; IC_{50} = 6.28 ± 0.29, 7.36 ± 0.34 μM against CAKI-1 and A498 cell lines, respectively

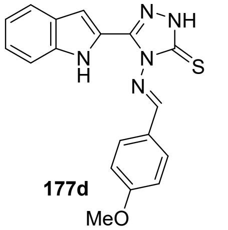

177d

IC_{50} = 0.064 ± 0.002 μM against VEGFR-2; IC_{50} = 1.63 ± 0.08, 20.6 ± 0.95 μM against CAKI-1 and A498 cell lines, respectively

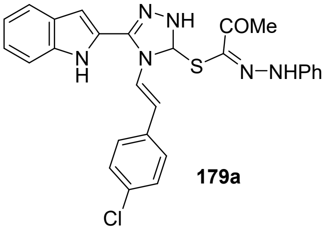

179a

IC_{50} = 0.148 ± 0.004 μM against VEGFR-2

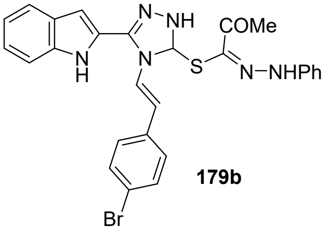

179b

IC_{50} = 0.175 ± 0.005 μM against VEGFR-2

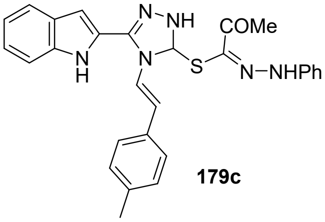

179c

IC_{50} = 0.075 ± 0.002 μM against VEGFR-2; IC_{50} = 3.23 ± 0.15, 2.05 ± 0.09 μM against CAKI-1 and A498 cell lines, respectively

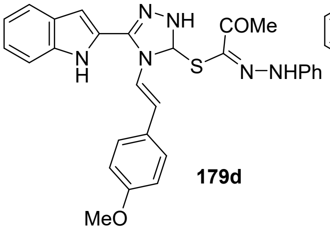

179d

IC_{50} = 0.199 ± 0.005 μM against VEGFR-2

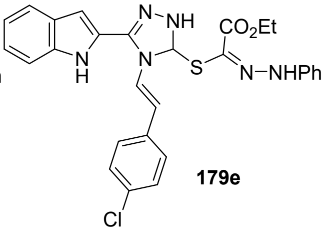

179e

IC_{50} = 0.137 ± 0.004 μM against VEGFR-2

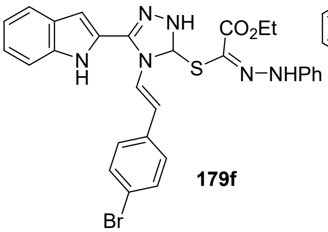

179f

IC_{50} = 0.046 ± 0.001 μM against VEGFR-2; IC_{50} = 32.1 ± 1.48, 4.9 ± 0.23 μM against CAKI-1 and A498 cell lines, respectively

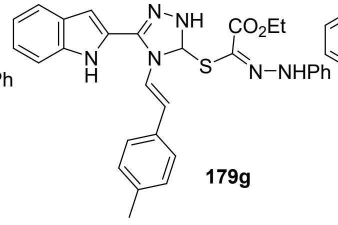

179g

IC_{50} = 0.157 ± 0.004 μM against VEGFR-2

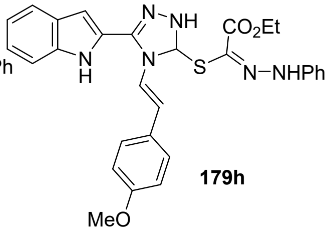

179h

IC_{50} = 0.077 ± 0.002 μM against VEGFR-2; IC_{50} = 8.46 ± 0.39, 0.64 ± 0.03 μM against CAKI-1 and A498 cell lines, respectively

Fig. S13. VEGFR-2 inhibitory and antiproliferation properties of indole triazole conjugates **176**, **177**, **179**, **181** and Sunitinib.

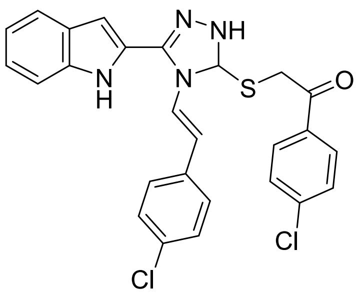

181a

IC_{50} = 0.248 ± 0.007 μM against VEGFR-2

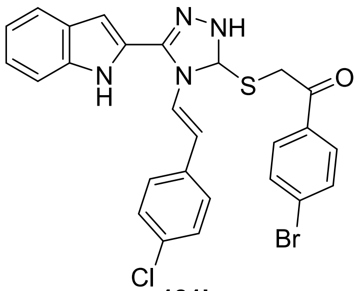

181b

IC_{50} = 0.152 ± 0.004 μM against VEGFR-2

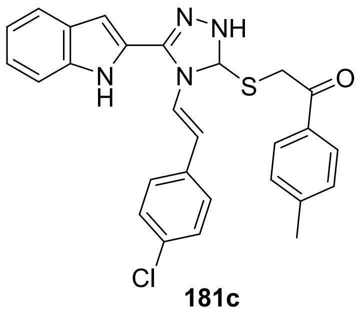

181c

IC_{50} = 0.108 ± 0.003 μM against VEGFR-2; IC_{50} = 2.03 ± 0.09, 5.27 ± 0.24 μM against CAKI-1 and A498 cell lines, respectively

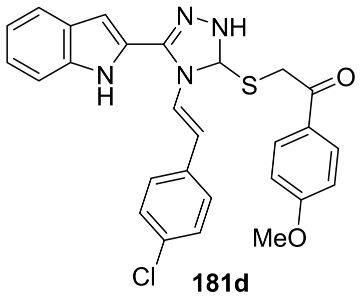

181d

IC_{50} = 0.256 ± 0.007 μM against VEGFR-2

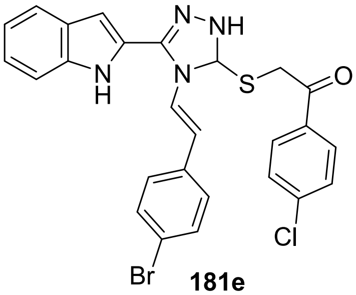

181e

IC_{50} = 0.131 ± 0.004 μM against VEGFR-2

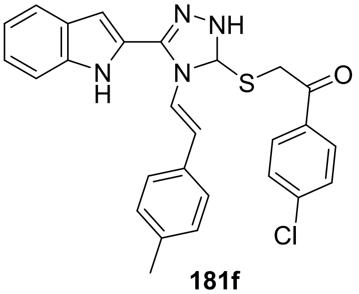

181f

IC_{50} = 0.258 ± 0.007 μM against VEGFR-2

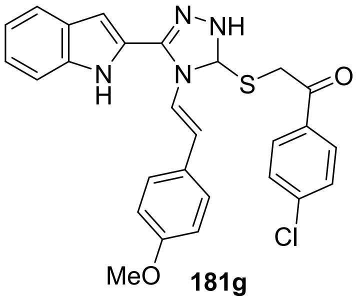

181g

IC_{50} = 0.071 ± 0.002 μM against VEGFR-2; IC_{50} = 0.89 ± 0.04, 2.2 ± 0.1 μM against CAKI-1 and A498 cell lines, respectively

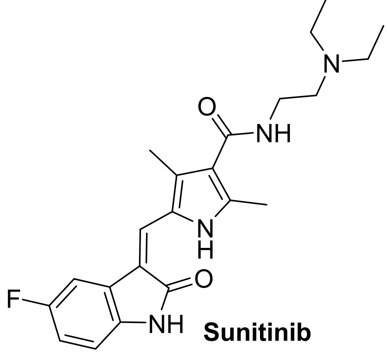

Sunitinib

IC_{50} = 0.075 ± 0.002 μM against VEGFR-2; IC_{50} = 4.93 ± 0.16, 1.25 ± 0.04 μM against CAKI-1 and A498 cell lines, respectively

Fig. S13 (continued). VEGFR-2 inhibitory and antiproliferation properties of indole triazole conjugates **176**, **177**, **179**, **181** and Sunitinib.

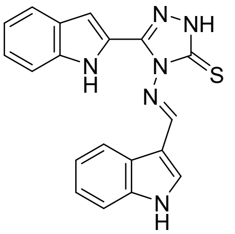

177a

IC_{50} = 3.06 ± 0.39, ≥100 μM againt MCF7 and HepG2 cell lines; IC_{50} = 103.35 ± 4.25 nM against VEGFR-2, respectively

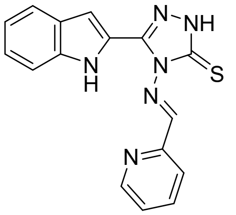

177b

IC_{50} = 18.9 ± 1.04, ≥50 μM againt MCF7 and HepG2 cell lines, respectively

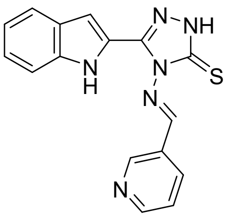

177c

IC_{50} = 20.8 ± 1.35, ≥50 μM againt MCF7 and HepG2 cell lines, respectively

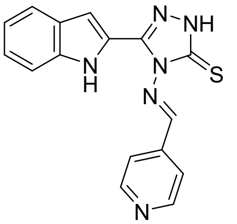

177d

IC_{50} = 17.97 ± 1.34, ≥50 μM againt MCF7 and HepG2 cell lines, respectively

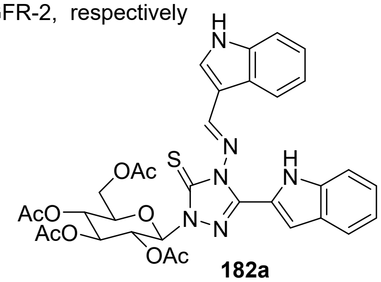

182a

IC_{50} = 45.8 ± 2.03, 43.8 ± 1.68 μM againt MCF7 and HepG2 cell lines, respectively

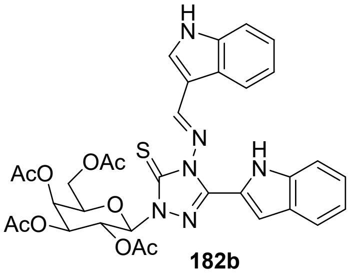

182b

IC_{50} = 35.8 ± 1.39, ≥100 μM againt MCF7 and HepG2 cell lines, respectively

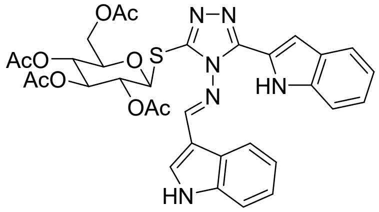

183a

IC_{50} = ≥50, 23.4 ± 0.97 μM againt MCF7 and HepG2 cell lines, respectively

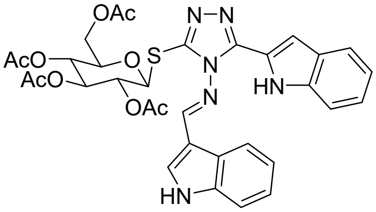

183b

IC_{50} = ≥100, 48.1 ± 1.95 μM againt MCF7 and HepG2 cell lines, respectively

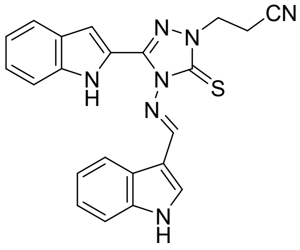

184

IC_{50} = ≥50, 20.76 ± 1.21 μM againt MCF7 and HepG2 cell lines, respectively

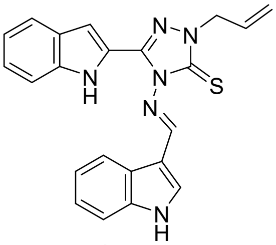

185

IC_{50} = 1.18 ± 0.15, 7.09 ± 0.67 μM againt MCF7 and HepG2 cell lines; IC_{50} = 19.8 ± 1.78 nM against VEGFR-2, respectively

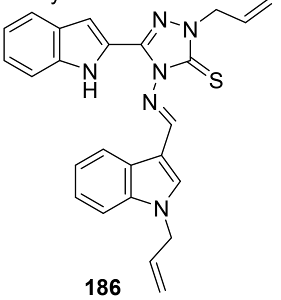

186

IC_{50} = 3.02 ± 0.22, ≥50 μM againt MCF7 and HepG2 cell lines; IC_{50} = 73.24 ± 2.04 nM against VEGFR-2, respectively

Fig. S14. Antiproliferation and VEGFR-2 inhibitory properties of indole triazole conjugates **177**, **183–186** and standard references.

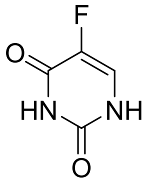

5- Fluorouracil

IC_{50} = 5.81 ± 0.65, 6.98 ± 0.26 μM againt MCF7 and HepG2 cell lines respectively

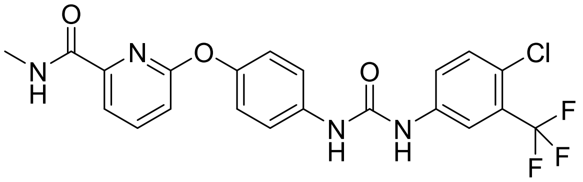

Sorafenib

IC_{50} = 2.13 ± 0.24, 3.24 ± 0.23 μM againt MCF7 and HepG2 cell lines; IC_{50} = 30.0 nM against VEGFR-2, respectively

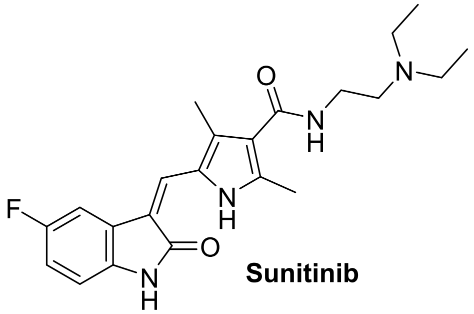

Sunitinib

IC_{50} = 75.0 nM against VEGFR-2

Fig. S14 (continued). Antiproliferation and VEGFR-2 inhibitory properties of indole triazole conjugates **177**, **183–186** and standard references.

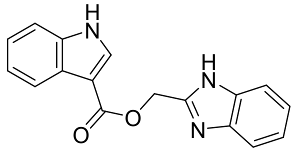

191a

% Inhibition of VEGFR-2 =
22.2 at 10 μM

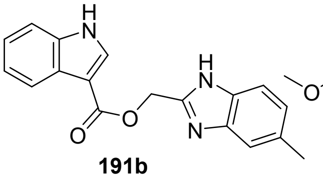

191b

% Inhibition of VEGFR-2 =
12.0 at 10 μM

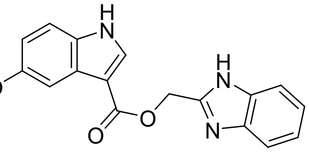

191c

% Inhibition of VEGFR-2 =
8.0 at 10 μM

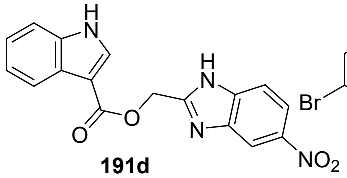

191d

% Inhibition of VEGFR-2 =
10.0 at 10 μM

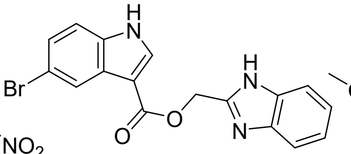

191e

% Inhibition of VEGFR-2 =
10.5 at 10 μM

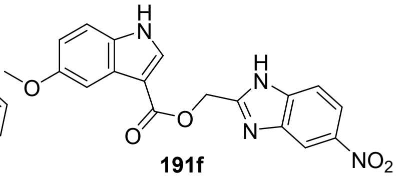

191f

% Inhibition of VEGFR-2 =
21.5 at 10 μM

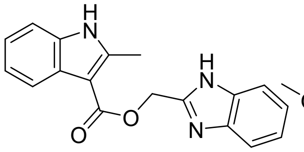

191g

% Inhibition of VEGFR-2 =
11.7 at 10 μM

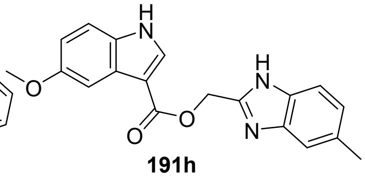

191h

% Inhibition of VEGFR-2 =
9.5 at 10 μM

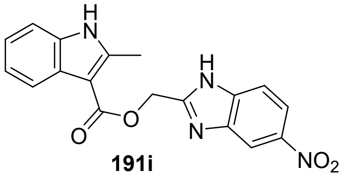

191i

% Inhibition of VEGFR-2 =
32.5 at 10 μM

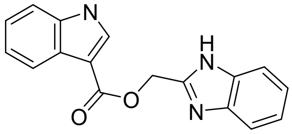

191j

% Inhibition of VEGFR-2 =
6.0 at 10 μM

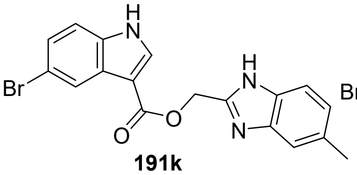

191k

% Inhibition of VEGFR-2 =
14.6 at 10 μM

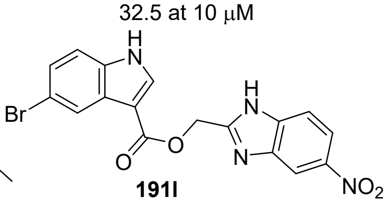

191l

% Inhibition of VEGFR-2 =
29.4 at 10 μM

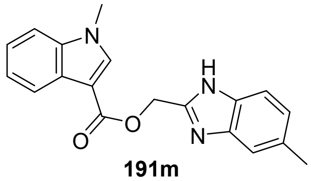

191m

% Inhibition of VEGFR-2 =
33.4 at 10 μM

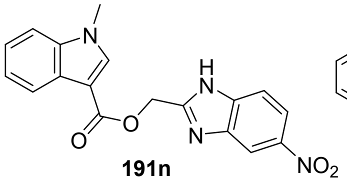

191n

% Inhibition of VEGFR-2 =
31.8 at 10 μM

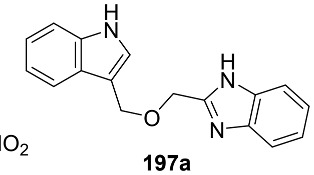

197a

% Inhibition of VEGFR-2 =
23.3 at 10 μM

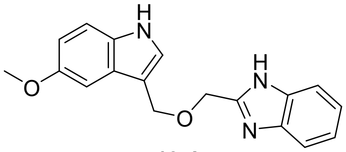

197b

% Inhibition of VEGFR-2 =
21.7 at 10 μM

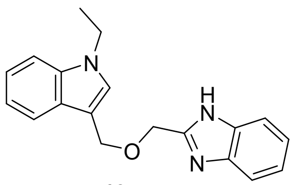

197c

% Inhibition of VEGFR-2 =
18.2 at 10 μM

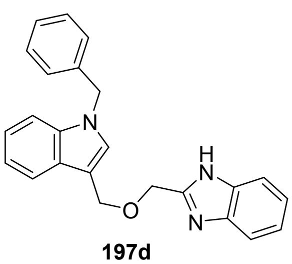

197d

% Inhibition of VEGFR-2 =
16.1 at 10 μM

Fig. S15. % Inhibitory properties of VEGFR-2 by indole benzimidazole conjugates **191** and **197** at 10 μM.

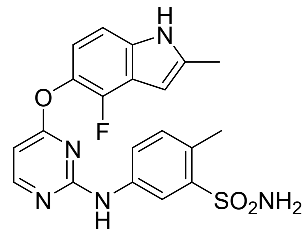

211a

IC_{50} = 0.039 ± 0.001 μM

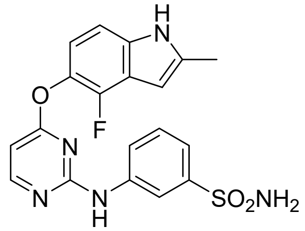

211b

IC_{50} = 0.009 ± 0.004 μM

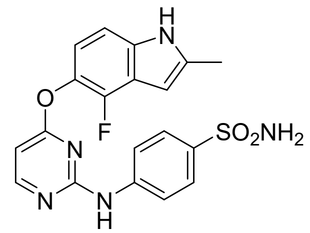

211c

IC_{50} = 0.031 ± 0.001 μM

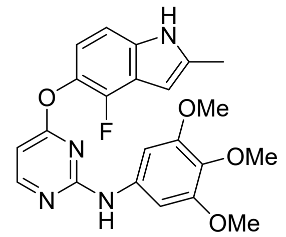

211d

IC_{50} = 0.009 ± 0.001 μM

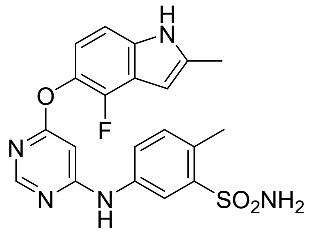

211e

IC_{50} = 0.0065 ± 0.0011 μM

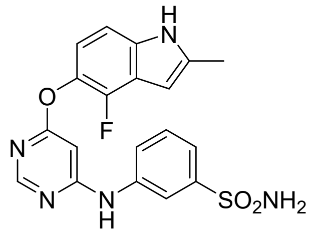

211f

IC_{50} = 0.005 ± 0.001 μM

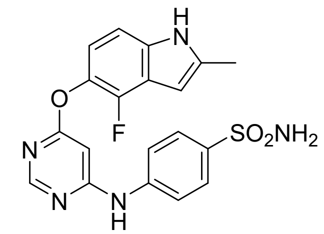

211g

IC_{50} = 0.0404 ± 0.0055 μM

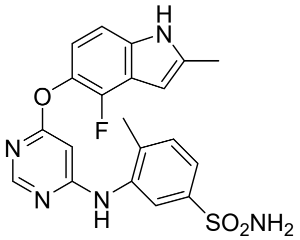

211h

IC_{50} = 0.0068 ± 0.0004 μM

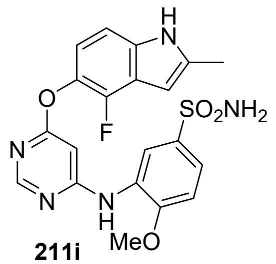

211i

IC_{50} = 0.0103 ± 0.0008 μM

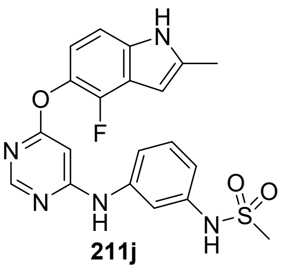

211j

IC_{50} = 0.0077 ± 0.0018 μM

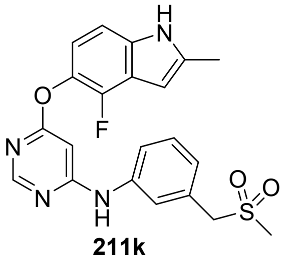

211k

IC_{50} = 0.0038 ± 0.0033 μM

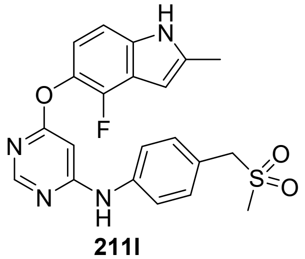

211l

IC_{50} = 0.0071 ± 0.0008 μM

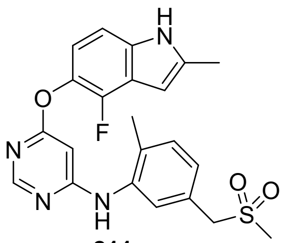

211m

IC_{50} = 0.0838 ± 0.0084 μM

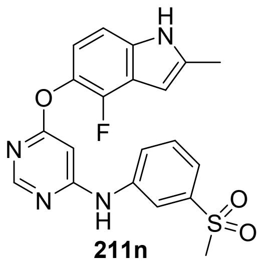

211n

IC_{50} = 0.0073 ± 0.0007 μM

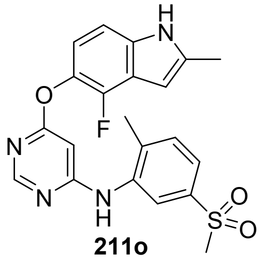

211o

IC_{50} = 0.0084 ± 0.0018 μM

Fig. S16. Inhibitory properties of VEGFR-2 by indole-pyrimidine conjugates **211** and Sunitinib.

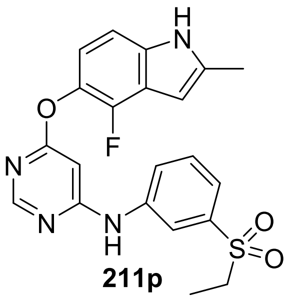

211p

IC$_{50}$ = 0.0060 ± 0.0004 μM

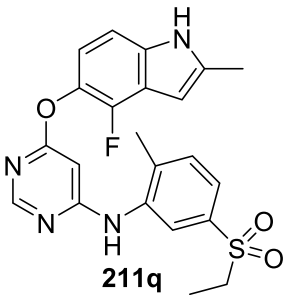

211q

IC$_{50}$ = 0.0046 ± 0.0023 μM

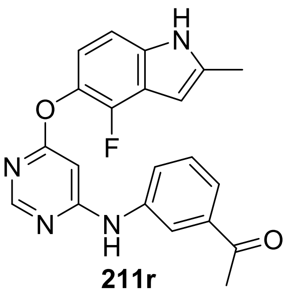

211r

IC$_{50}$ = 0.0107 ± 0.0062 μM

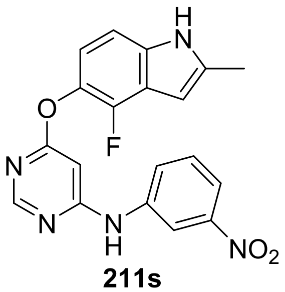

211s

IC$_{50}$ = 0.0213 ± 0.0033 μM

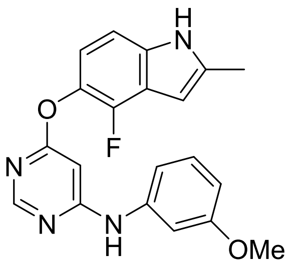

211t

IC$_{50}$ = 0.0088 ± 0.0019 μM

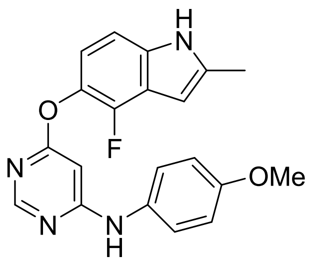

211u

IC$_{50}$ = 0.0221 ± 0.0077 μM

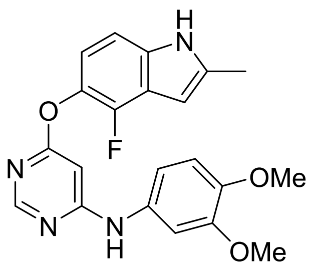

211v

IC$_{50}$ = 0.0049 ± 0.0006 μM

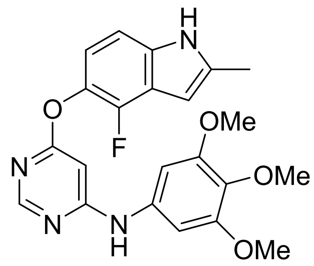

211w

IC$_{50}$ = 0.0065 ± 0.0011 μM

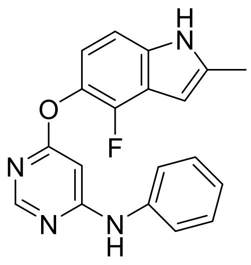

211x

IC$_{50}$ = 0.0121 ± 0.0017 μM

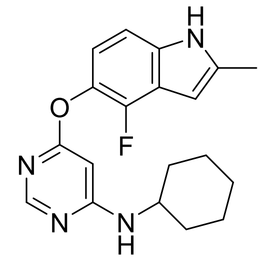

211y

IC$_{50}$ = 0.0351 ± 0.0027 μM

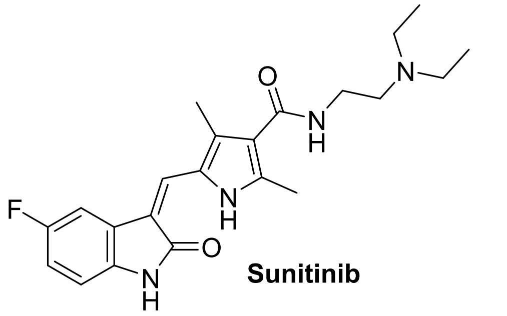

Sunitinib

IC$_{50}$ = 0.0022 ± 0.0005 μM

Fig. S16 (continued). Inhibitory properties of VEGFR-2 by indole-pyrimidine conjugates **211** and Sunitinib.

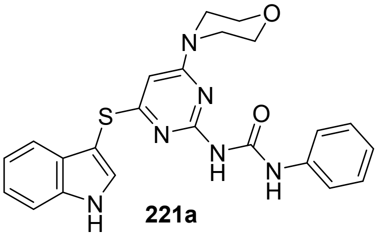

221a

IC_{50} = 26.12 ± 2.12, 14.13 ± 1.81, 12.14 ± 1.21, 7.14 ± 0.62 μM against A549, PC-3, MDAMB-231 and HepG2, respectively; % inhibition = 1.21 ± 0.14 of VEGFR-2 at 10 μM

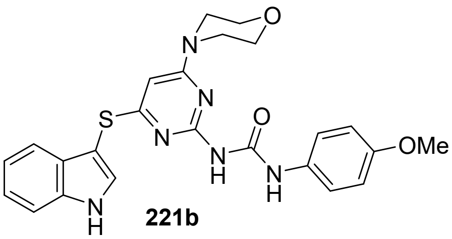

221b

IC_{50} = 33.40 ± 5.16, 12.46 ± 2.11, 7.94 ± 0.92, 10.26 ± 0.81 μM against A549, PC-3, MDAMB-231 and HepG2, respectively; % inhibition = 1.92 ± 0.15 of VEGFR-2 at 10 μM

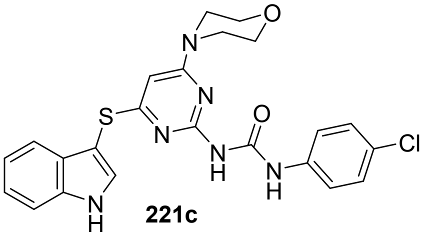

221c

IC_{50} = 21.83 ± 1.66, 28.44 ± 3.01, 6.93 ± 0.51, 22.47 ± 1.27 μM against A549, PC-3, MDAMB-231 and HepG2, respectively; % inhibition = 1.44 ± 0.21 of VEGFR-2 at 10 μM

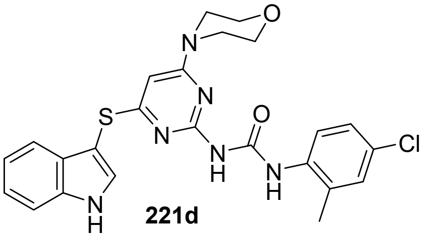

221d

IC_{50} = >50, 33.81 ± 2.51, 8.65 ± 0.62, 8.26 ± 0.97 μM against A549, PC-3, MDAMB-231 and HepG2, respectively; % inhibition = 0.35 ± 0.11 of VEGFR-2 at 10 μM

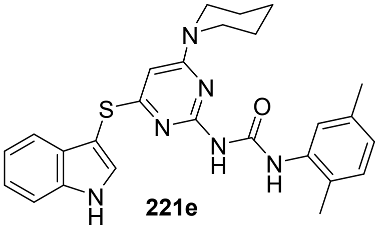

221e

IC_{50} = 25.40 ± 3.54, >50, 5.94 ± 0.81, 6.44 ± 0.44 μM against A549, PC-3, MDAMB-231 and HepG2, respectively; % inhibition = 0.31 ± 0.07 of VEGFR-2 at 10 μM

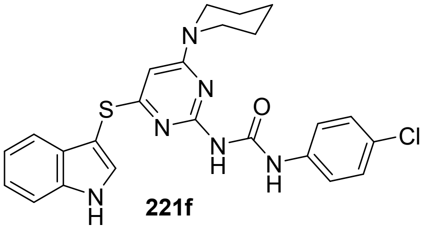

221f

IC_{50} = 18.62 ± 1.44, 42.54 ± 5.21, 10.13 ± 1.21, 14.81 ± 1.89 μM against A549, PC-3, MDAMB-231 and HepG2, respectively; % inhibition = 1.82 ± 0.21 of VEGFR-2 at 10 μM

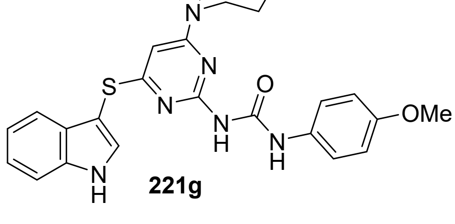

221g

IC_{50} = 13.47 ± 0.97, 17.40 ± 2.11, 7.26 ± 1.12, 17.12 ± 1.11 μM against A549, PC-3, MDAMB-231 and HepG2, respectively; % inhibition = 1.13 ± 0.24 of VEGFR-2 at 10 μM

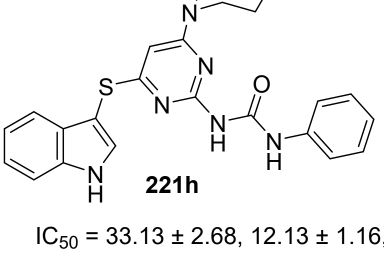

221h

IC_{50} = 33.13 ± 2.68, 12.13 ± 1.16, 8.13 ± 1.14, 20.86 ± 0.91 μM against A549, PC-3, MDAMB-231 and HepG2, respectively; % inhibition = 4.55 ± 0.32 of VEGFR-2 at 10 μM

Fig. S17. Antiproliferation and inhibitory properties of VEGFR-2 for indole pyrimidine conjugates **221** and Sorafenib.

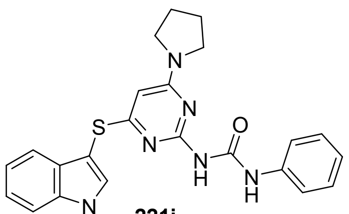

221i

IC_{50} = 21.24 ± 1.51, 14.93 ± 0.92, 7.89 ± 0.69, 11.44 ± 1.22 μM against A549, PC-3, MDAMB-231 and HepG2, respectively; % inhibition = 3.23 ± 0.28 of VEGFR-2 at 10 μM

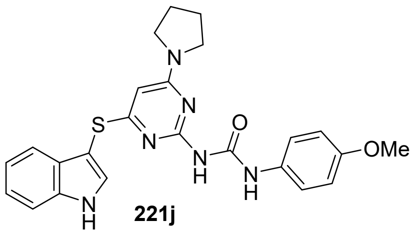

221j

IC_{50} = 11.82 ± 0.96, 21.48 ± 1.12, 13.10 ± 0.92, 20.26 ± 1.17 μM against A549, PC-3, MDAMB-231 and HepG2, respectively; % inhibition = 2.61 ± 0.33 of VEGFR-2 at 10 μM

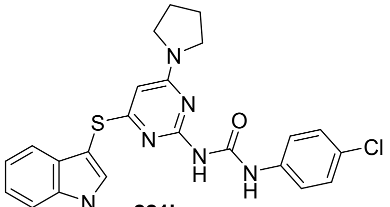

221k

IC_{50} = 6.41 ± 0.81, 10.42 ± 0.78, 5.85 ± 0.71, 7.87 ± 1.18 μM against A549, PC-3, MDAMB-231 and HepG2, respectively; % inhibition = 0.33 ± 0.04 of VEGFR-2 at 10 μM

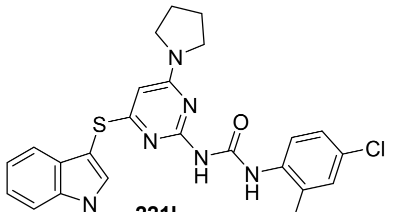

221l

IC_{50} = 8.93 ± 1.21, 12.86 ± 1.19, 9.44 ± 1.14, 7.15 ± 0.95 μM against A549, PC-3, MDAMB-231 and HepG2, respectively; % inhibition = 0.43 ± 0.05 of VEGFR-2 at 10 μM

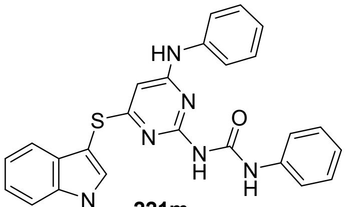

221m

IC_{50} = >50, 26.43 ± 2.12, 35.92 ± 1.43, 14.40 ± 0.92 μM against A549, PC-3, MDAMB-231 and HepG2, respectively; % inhibition = 2.74 ± 0.23 of VEGFR-2 at 10 μM

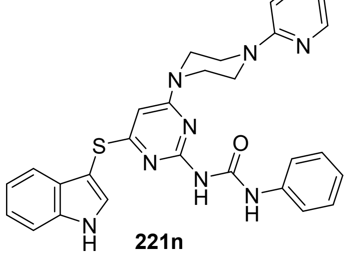

221n

IC_{50} = 30.25 ± 4.25, 21.64 ± 1.55, 19.51 ± 1.38, 8.93 ± 0.81 μM against A549, PC-3, MDAMB-231 and HepG2, respectively; % inhibition = 1.71 ± 0.21 of VEGFR-2 at 10 μM

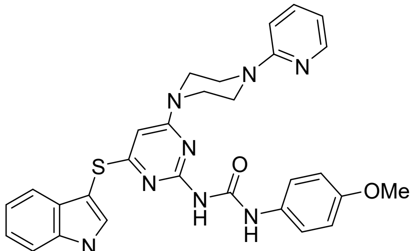

221o

IC_{50} = >50, 33.43 ± 2.86, 28.42 ± 2.01, 11.42 ± 1.61 μM against A549, PC-3, MDAMB-231 and HepG2, respectively; % inhibition = 4.15 ± 0.34 of VEGFR-2 at 10 μM

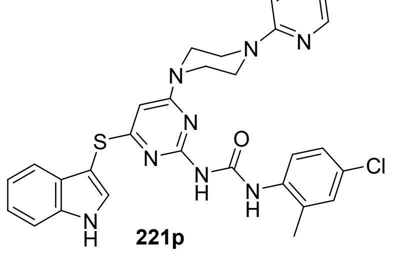

221p

IC_{50} = >50, 19.81 ± 1.32, 34.70 ± 2.31, 7.64 ± 0.64 μM against A549, PC-3, MDAMB-231 and HepG2, respectively; % inhibition = 0.92 ± 0.11 of VEGFR-2 at 10 μM

Fig. S17 (continued). Antiproliferation and inhibitory properties of VEGFR-2 for indole pyrimidine conjugates **221** and Sorafenib.

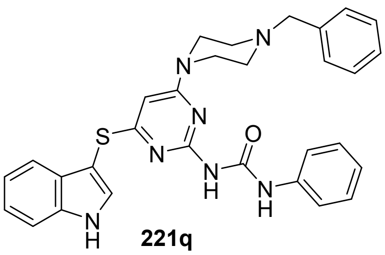

221q

IC_{50} = >50, 42.47 ± 3.31, 10.22 ± 0.82, 8.16 ± 1.31 μM against A549, PC-3, MDAMB-231 and HepG2, respectively; % inhibition = 0.67 ± 0.08 of VEGFR-2 at 10 μM

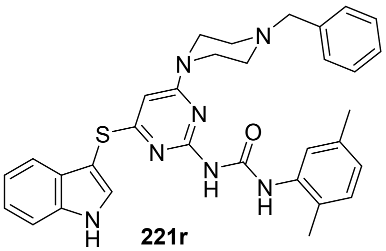

221r

IC_{50} = >50, 17.29 ± 0.92, 18.96 ± 1.43, 16.35 ± 1.23 μM against A549, PC-3, MDAMB-231 and HepG2, respectively; % inhibition = 2.14 ± 0.21 of VEGFR-2 at 10 μM

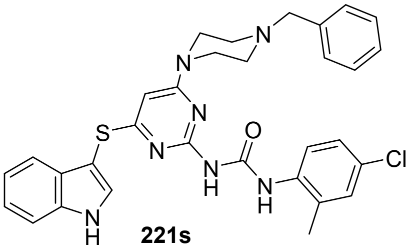

221s

IC_{50} = >50, 22.45 ± 1.42, 22.13 ± 1.52, 11.74 ± 1.61 μM against A549, PC-3, MDAMB-231 and HepG2, respectively; % inhibition = 4.86 ± 0.27 of VEGFR-2 at 10 μM

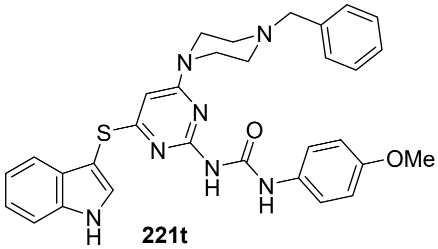

221t

IC_{50} = 42.31 ± 3.16, 16.73 ± 1.93, 8.92 ± 0.61, 21.42 ± 1.73 μM against A549, PC-3, MDAMB-231 and HepG2, respectively; % inhibition = 0.84 ± 0.13 of VEGFR-2 at 10 μM

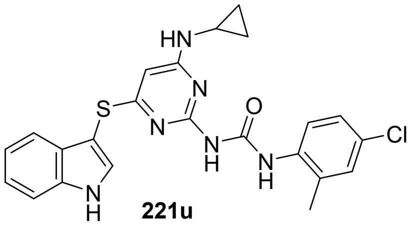

221u

IC_{50} = 31.49 ± 2.11, 11.12 ± 0.98, 14.61 ± 1.24, 9.74 ± 1.24 μM against A549, PC-3, MDAMB-231 and HepG2, respectively; % inhibition = 0.44 ± 0.07 of VEGFR-2 at 10 μM

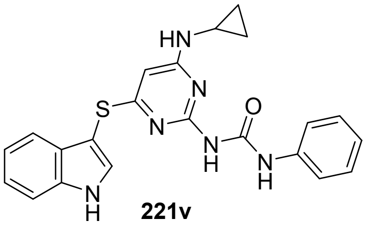

221v

IC_{50} = >50, 17.54 ± 1.21, 7.86 ± 1.11, 14.32 ± 1.97 μM against A549, PC-3, MDAMB-231 and HepG2, respectively; % inhibition = 5.22 ± 0.42 of VEGFR-2 at 10 μM

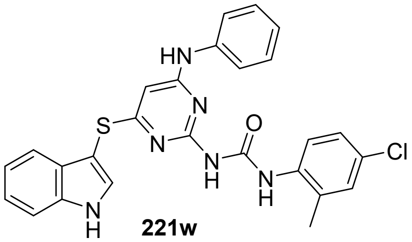

221w

IC_{50} = 23.45 ± 3.17, 18.45 ± 2.72, 13.42 ± 0.93, 14.90 ± 1.19 μM against A549, PC-3, MDAMB-231 and HepG2, respectively; % inhibition = 1.33 ± 0.18 of VEGFR-2 at 10 μM

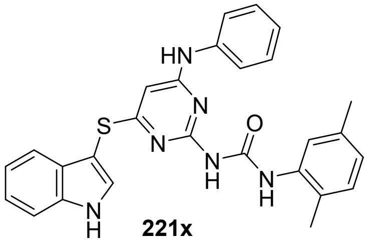

221x

IC_{50} = >50, 41.86 ± 5.92, 17.10 ± 1.45, 8.37 ± 0.58 μM against A549, PC-3, MDAMB-231 and HepG2, respectively; % inhibition = 2.82 ± 0.31 of VEGFR-2 at 10 μM

Fig. S17 (continued). Antiproliferation and inhibitory properties of VEGFR-2 for indole pyrimidine conjugates **221** and Sorafenib.

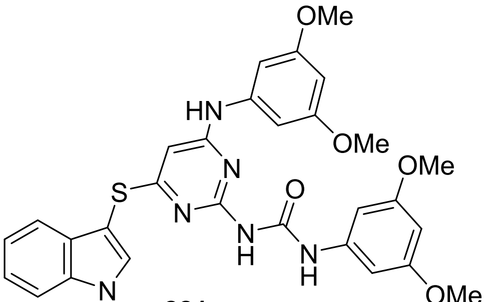

221y

IC_{50} = >50, 11.64 ± 2.17, >50, 24.82 ± 3.11 μM against A549, PC-3, MDAMB-231 and HepG2, respectively; % inhibition = 3.51 ± 0.34 of VEGFR-2 at 10 μM

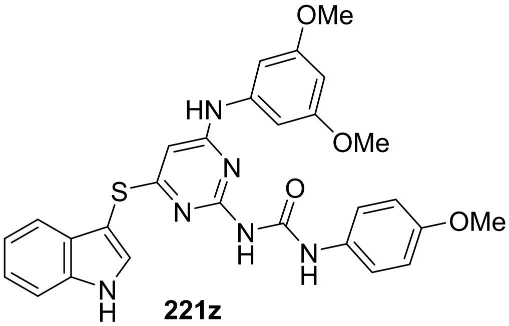

221z

IC_{50} = >50, 15.10 ± 0.93, 9.46 ± 1.21, 17.40 ± 2.02 μM against A549, PC-3, MDAMB-231 and HepG2, respectively; % inhibition = 1.43 ± 0.21 of VEGFR-2 at 10 μM

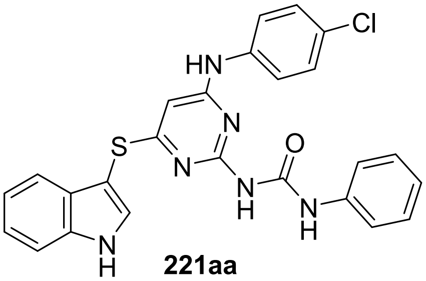

221aa

IC_{50} = 48.43 ± 6.91, 32..28 ± 4.11, 10.26 ± 1.46, 12.63 ± 0.93 μM against A549, PC-3, MDAMB-231 and HepG2, respectively; % inhibition = 1.81 ± 0.16 of VEGFR-2 at 10 μM

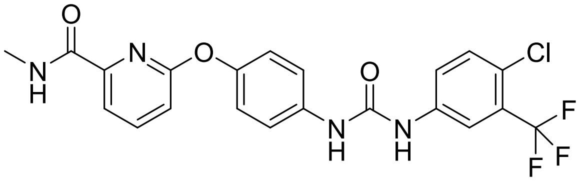

Sorafenib

IC_{50} = 7.43 ± 0.81, 9.77 ± 1.12, 11.84 ± 1.25, 5.78 ± 0.41 μM against A549, PC-3, MDAMB-231 and HepG2, respectively; % inhibition = 1.21 ± 0.02 of VEGFR-2 at 10 μM

Fig. S17 (continued). Antiproliferation and inhibitory properties of VEGFR-2 for indole pyrimidine conjugates **221** and Sorafenib.

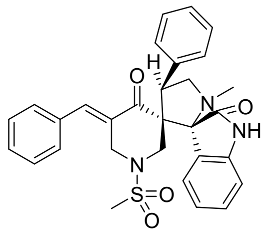

230a

IC_{50} = 19.787 ± 0.99, 15.957 ± 1.10, 32.340 ± 1.14, 48.404 ± 2.23, >50.000 ± 1.09 μM against MCF7, HCT116, A431, PaCa-2 and RPE1 cell lines, respectively; % inhibition = 60.9, 66.4 against VEGFR-2 and EGFR at IC_{50} value observed against MCF7, respectively.

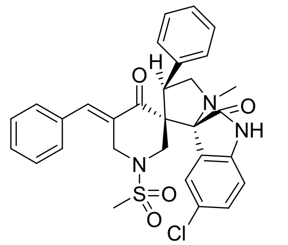

230b

IC_{50} = 7.660 ± 0.68, 6.915 ± 0.52, 9.149 ± 0.70, 20.638 ± 1.17, 24.681 ± 1.26 μM against MCF7, HCT116, A431, PaCa-2 and RPE1 cell lines, respectively; % inhibition = 64.2, 54.1 against VEGFR-2 and EGFR at IC_{50} value observed against MCF7, respectively.

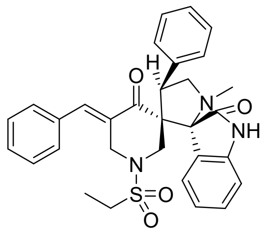

230c

IC_{50} = >50.000 ± 1.94, 6.125 ± 0.44, 33.191 ± 0.91, >50.000 ± 2.00, >50.000 ± 2.00 μM against MCF7, HCT116, A431, PaCa-2 and RPE1 cell lines, respectively; % inhibition = 63.8, 69.6 against VEGFR-2 and EGFR at IC_{50} value observed against MCF7, respectively.

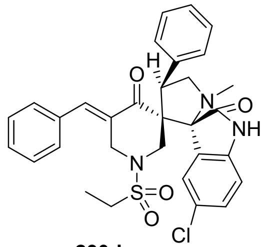

230d

IC_{50} = 6.915 ± 0.55, 5.181 ± 0.61, 4.958 ± 0.25, 13.085 ± 1.10, >50.000 ± 1.85 μM against MCF7, HCT116, A431, PaCa-2 and RPE1 cell lines, respectively; % inhibition = 63.8, 62.9 against VEGFR-2 and EGFR at IC_{50} value observed against MCF7, respectively.

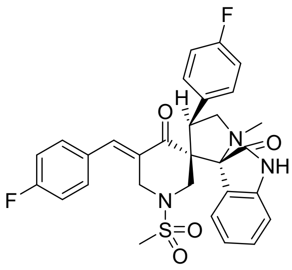

230e

IC_{50} = 15.532 ± 0.76, 9.894 ± 0.85, 16.064 ± 0.99, 39.894 ± 1.89, 49.043 ± 1.11 μM against MCF7, HCT116, A431, PaCa-2 and RPE1 cell lines, respectively; % inhibition = 64.5, 66.7 against VEGFR-2 and EGFR at IC_{50} value observed against MCF7, respectively.

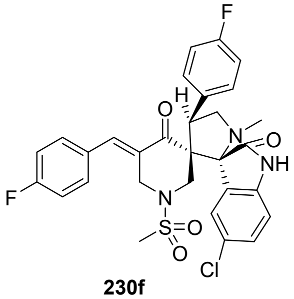

230f

IC_{50} = 5.000 ± 0.39, 5.431 ± 0.46, 4.764 ± 0.37, 11.702 ± 0.94, 17.766 ± 0.87 μM against MCF7, HCT116, A431, PaCa-2 and RPE1 cell lines, respectively; % inhibition = 64.7, 69.3 against VEGFR-2 and EGFR at IC_{50} value observed against MCF7, respectively.

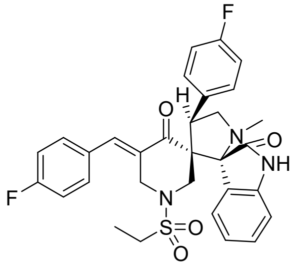

230g

IC_{50} = 10.319 ± 0.86, 4.944 ± 0.25, 6.167 ± 0.44, 28.404 ± 0.85, 33.404 ± 1.22 μM against MCF7, HCT116, A431, PaCa-2 and RPE1 cell lines, respectively; % inhibition = 63.7, 62.8 against VEGFR-2 and EGFR at IC_{50} value observed against MCF7, respectively.

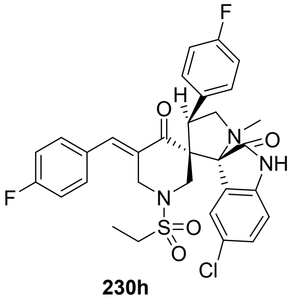

230h

IC_{50} = 4.694 ± 0.44, 4.597 ± 0.18, 6.042 ± 0.26, 14.043 ± 0.73, >50.000 ± 2.38 μM against MCF7, HCT116, A431, PaCa-2 and RPE1 cell lines, respectively; % inhibition = 66.2, 67.2 against VEGFR-2 and EGFR at IC_{50} value observed against MCF7, respectively.

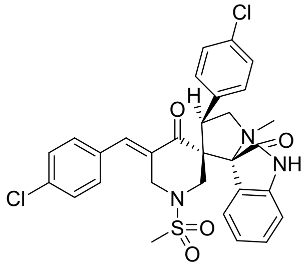

230i

IC_{50} = 5.014 ± 0.29, 5.472 ± 0.32, 4.403 ± 0.49, 9.043 ± 0.62, 14.787 ± 1.57 μM against MCF7, HCT116, A431, PaCa-2 and RPE1 cell lines, respectively; % inhibition = 64.2, 68.7 against VEGFR-2 and EGFR at IC_{50} value observed against MCF7, respectively.

Fig. S18. Antiproliferation and enzymatc inhibitory (VEGFR-2 and EGFR) properties of spiroindoles **230** and reference standards (Sunitinib and 5-Fluorouracil).

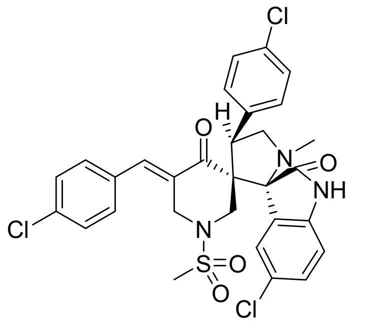

230j

IC_{50} = 4.514 ± 0.39, 4.722 ± 0.25, 4.083 ± 0.21, 8.830 ± 0.51, 12.500 ± 0.86 μM against MCF7, HCT116, A431, PaCa-2 and RPE1 cell lines, respectively; % inhibition = 57.9, 63.3 against VEGFR-2 and EGFR at IC_{50} value observed against MCF7, respectively.

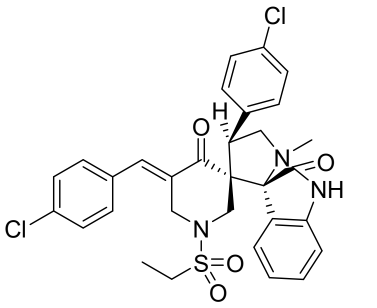

230k

IC_{50} = 4.375 ± 0.26, 4.167 ± 0.38, 2.966 ± 0.29, 8.830 ± 0.70, 14.792 ± 0.99 μM against MCF7, HCT116, A431, PaCa-2 and RPE1 cell lines, respectively; % inhibition = 66.2, 66.2 against VEGFR-2 and EGFR at IC_{50} value observed against MCF7, respectively.

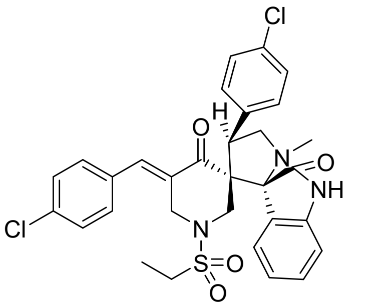

230l

IC_{50} = 4.375 ± 0.26, 4.167 ± 0.38, 2.966 ± 0.29, 8.830 ± 0.70, 14.792 ± 0.99 μM against MCF7, HCT116, A431, PaCa-2 and RPE1 cell lines, respectively; % inhibition = 66.2, 66.2 against VEGFR-2 and EGFR at IC_{50} value observed against MCF7, respectively.

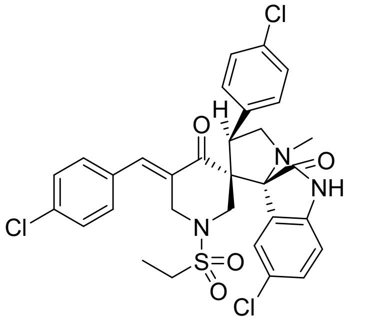

230m

IC_{50} = 3.986 ± 0.31, 4.111 ± 0.41, 3.694 ± 0.33, 11.915 ± 0.83, >50.000 ± 2.32 μM against MCF7, HCT116, A431, PaCa-2 and RPE1 cell lines, respectively; % inhibition = 60.2, 65.7 against VEGFR-2 and EGFR at IC_{50} value observed against MCF7, respectively.

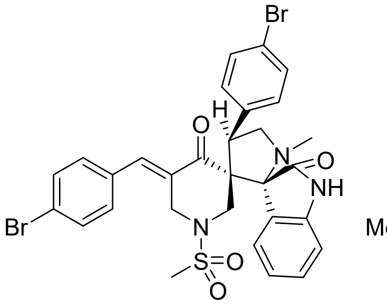

230n

IC_{50} = 3.597 ± 0.19, 3.236 ± 0.27, 2.434 ± 0.18, 12.500 ± 0.67, 14.894 ± 1.61 μM against MCF7, HCT116, A431, PaCa-2 and RPE1 cell lines, respectively; % inhibition = 61.3, 65.6 against VEGFR-2 and EGFR at IC_{50} value observed against MCF7, respectively.

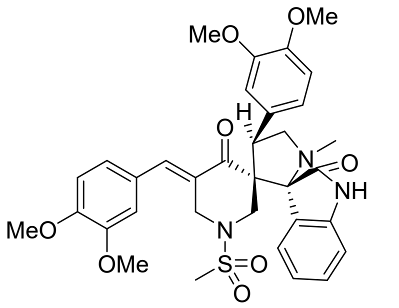

230o

IC_{50} = 40.213 ± 1.10, 15.426 ± 0.52, 34.894 ± 1.36, 32.766 ± 1.21, >50.000 ± 2.21 μM against MCF7, HCT116, A431, PaCa-2 and RPE1 cell lines, respectively; % inhibition = 64.2, 69.6 against VEGFR-2 and EGFR at IC_{50} value observed against MCF7, respectively.

Fig. S18 (continued). Antiproliferation and enzymatc inhibitory (VEGFR-2 and EGFR) properties of spiroindoles **230** and reference standards (Sunitinib and 5-Fluorouracil).

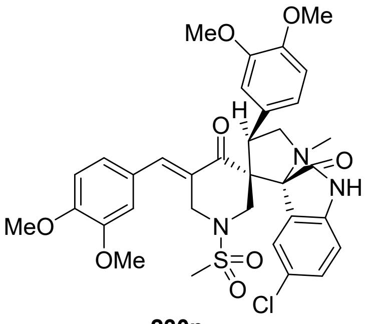

230p

IC_{50} = 48.936 ± 1.84, 28.511 ± 0.75, 45.417 ± 1.84, >50.000 ± 2.31, >50.000 ± 2.61 μM against MCF7, HCT116, A431, PaCa-2 and RPE1 cell lines, respectively; % inhibition = 61.8, 65.9 against VEGFR-2 and EGFR at IC_{50} value observed against MCF7, respectively.

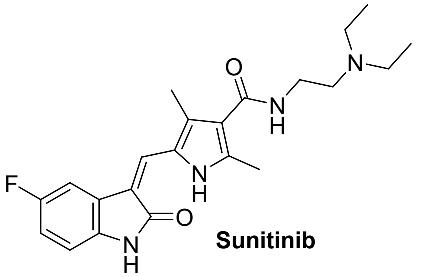

Sunitinib

IC_{50} = 3.97 ± 0.32, 9.67 ± 0.22, 16.91 ± 0.95 μM against MCF7, HCT116, and PaCa-2 cell lines, respectively; % inhibition = 74.7, 81.4 against VEGFR-2 and EGFR at IC_{50} value observed against MCF7, respectively.

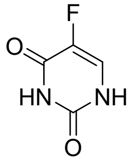

5- Fluorouracil

IC_{50} = 3.15 ± 0.44, 20.43 ± 1.99, 23.44 ± 2.09 μM against MCF7, HCT116, and A431 cell lines, respectively.

Fig. S18 (continued). Antiproliferation and enzymatc inhibitory (VEGFR-2 and EGFR) properties of spiroindoles **230** and reference standards (Sunitinib and 5-Fluorouracil).
